# *Arid1a* Deficiency Drives Aristolochic Acid‐Induced Liver Tumorigenesis through *Ctnnb1* Mutation and Defective Nucleotide Excision Repair

**DOI:** 10.1002/advs.202513981

**Published:** 2025-10-24

**Authors:** Lan Wang, Shi‐Hao Bai, Shu‐Jin Song, Xiao‐Li Zhang, Xue‐Ying Shang, Zhao‐Ning Lu, Xiao‐Fang Cui, Xin‐Le Zhu, Ze‐Guang Han

**Affiliations:** ^1^ Key Laboratory of Systems Biomedicine (Ministry of Education) and State Key Laboratory of Medical Genomics Shanghai Center for Systems Biomedicine Shanghai Jiao Tong University Shanghai 200240 China

**Keywords:** ARID1A, aristolochic acid, liver cancer, Nqo1, nucleotide excision repair, SWI/SNF complex

## Abstract

*ARID1A*, which encodes an important subunit of SWI/SNF complex, is frequently mutated in non‐malignant tissues and tumors. However, how *ARID1A* loss enables environmental carcinogens to initiate tumorigenesis remains unknown. Here, liver‐specific *Arid1a*‐deficient (*Arid1a^LKO^*) mice are exposed to aristolochic acid I (AAI), a potent herbal carcinogen. Notably, AAI dramatically accelerated hepatocarcinogenesis in *Arid1a*‐deficient livers, accompanied by a specific 3′ splice‐site mutation in *Ctnnb1* in most tumors and adjacent non‐tumorous tissues. This mutation results in exon 3 skipping and subsequent β‐catenin activation. Single‐nucleus RNA‐seq coupled with phylogenetic analyses reveals AAI‐induced tumor microenvironment alteration and clonal expansion of β‐catenin‐activated cells. Conversely, inhibition of β‐catenin signaling significantly suppresses AAI‐induced tumors in the context of *Arid1a* loss. Mechanistically, *Arid1a* deficiency transcriptionally represses the expression of critical genes related to nucleotide excision repair, which removes AAI‐derived DNA adducts, due to SWI/SNF complex dysfunction. Simultaneously, it upregulates Nqo1, a key enzyme enhancing AAI bioactivation and AAI‐DNA adduct formation. This dual‐hit mechanism, characterized by impaired DNA repair and heightened genotoxicity, explains synergistic carcinogenesis. The study unveils ARID1A as a guardian against environmental carcinogens and proposes β‐catenin blockade for precision prevention in high‐risk patients with *ARID1A*‐mutant benign liver diseases.

## Graphical abstract

*ARID1A* is frequently mutated in both non‐malignant tissues and cancers, but its role in tumor development after exposure to genotoxic carcinogen remains unclear. It is found that aristolochic acid I accelerated liver tumorigenesis in *ARID1A*‐deficient context by impairing nucleotide excision repair and enhancing carcinogen bioactivation, revealing key mechanisms driving cancer development.

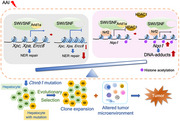

## 1 Introduction

SWI/SNF chromatin remodeling complexes regulate gene transcription by repositioning nucleosomes through ATP hydrolysis. Somatic mutations in SWI/SNF subunit genes occur in over 20% of all human cancers,^[^ ^1^ ^]^ with *ARID1A* being the most frequently mutated subunit across all cancer types.^[^ ^2^ ^]^ Notably, deleterious *ARID1A* mutations are frequently observed in morphologically normal tissues and non‐malignant cells of older individuals.^[^ ^3^, ^4^, ^5^, ^6^, ^7^ ^]^ This raises a critical question: can the loss of a single tumor suppressor gene like *ARID1A* alone transform healthy cells into cancer cells?

In cirrhotic livers, somatic *ARID1A* mutations promote clonal expansion, potentially independently of carcinogenesis,^[^ ^8^ ^]^ and may even confer protection against liver injury.^[^ ^9^ ^]^ These same *ARID1A*‐inactivating mutations are also found in liver cancer, suggesting positive selection during disease progression. Such clonal expansion in phenotypically normal or non‐tumorous tissues is often associated with aging, environmental insults, or chronic inflammation.^[^ ^4^ ^]^

To investigate the role of *ARID1A* loss in liver tumorigenesis, we and others developed a genetically engineered mouse model. This model revealed that liver‐specific *Arid1a* deficiency induced nonalcoholic steatohepatitis^[^ ^10^, ^11^ ^]^ and promoted hepatocellular carcinoma (HCC) in mice.^[^ ^12^, ^13^ ^]^ Paradoxically, *Arid1a* deletion has also been reported to enhance liver regeneration^[^ ^14^ ^]^ while inhibiting tumor initiation yet accelerating HCC progression and metastasis,^[^ ^15^ ^]^ indicating context‐dependent roles for Arid1a in liver cancer.^[^ ^16^ ^]^ These findings suggest that *Arid1a* mutations in chronic liver diseases may drive liver tumorigenesis under specific environmental exposures.

ARID1A is critical for DNA mismatch repair^[^ ^17^ ^]^ and double‐strand break repair.^[^ ^18^, ^19^ ^]^ We therefore hypothesize that environmental genotoxins could exacerbate DNA damage, promoting liver tumorigenesis in an *ARID1A*‐deficient contexts. Clinically relevant to this hypothesis, patients with chronic liver diseases and older individuals, particularly in China, are often exposed to traditional herbs containing aristolochic acids (AAs), classified as a Group 1 human carcinogens by the International Agency for Research on Cancer (IARC).^[^ ^20^ ^]^ The major AA derivatives, aristolochic acid I (AAI) and II (AAII), form bulky aristolactam (AL)‐DNA adducts after metabolic activation in vivo.^[^ ^21^ ^]^ AA‐induced mutations, characterized by A:T‐to‐T:A transversions (Catalogue of Somatic Mutations in Cancer, COSMIC, SBS22a), are found in morphologically normal human livers and other tissues,^[^ ^7^ ^]^ as well as in human and mouse liver cancers.^[^ ^22^, ^23^ ^]^ Based on this evidence, we proposed that AAs could rapidly induce liver tumorigenesis in the context of *ARID1A* mutations.

Here, we demonstrate that AAI rapidly initiates liver cancer in liver‐specific *Arid1a*‐deficient mice. This carcinogenesis is accompanied by a characteristic *Ctnnb1* mutation and alterations in liver microenvironment. Mechanistically, *Arid1a* loss downregulates key nucleotide excision repair (NER) genes and upregulates Nqo1, a critical enzyme for AA bioactivation in vivo. These findings elucidate the molecular and cellular mechanisms by which the exogenous carcinogen AAI drives liver tumorigenesis in an *Arid1a*‐deficient context.

## 2 Results

### 2.1 AA Promotes Liver Tumorigenesis in *Arid1a*‐Deficient Mice

**Figure 1:**
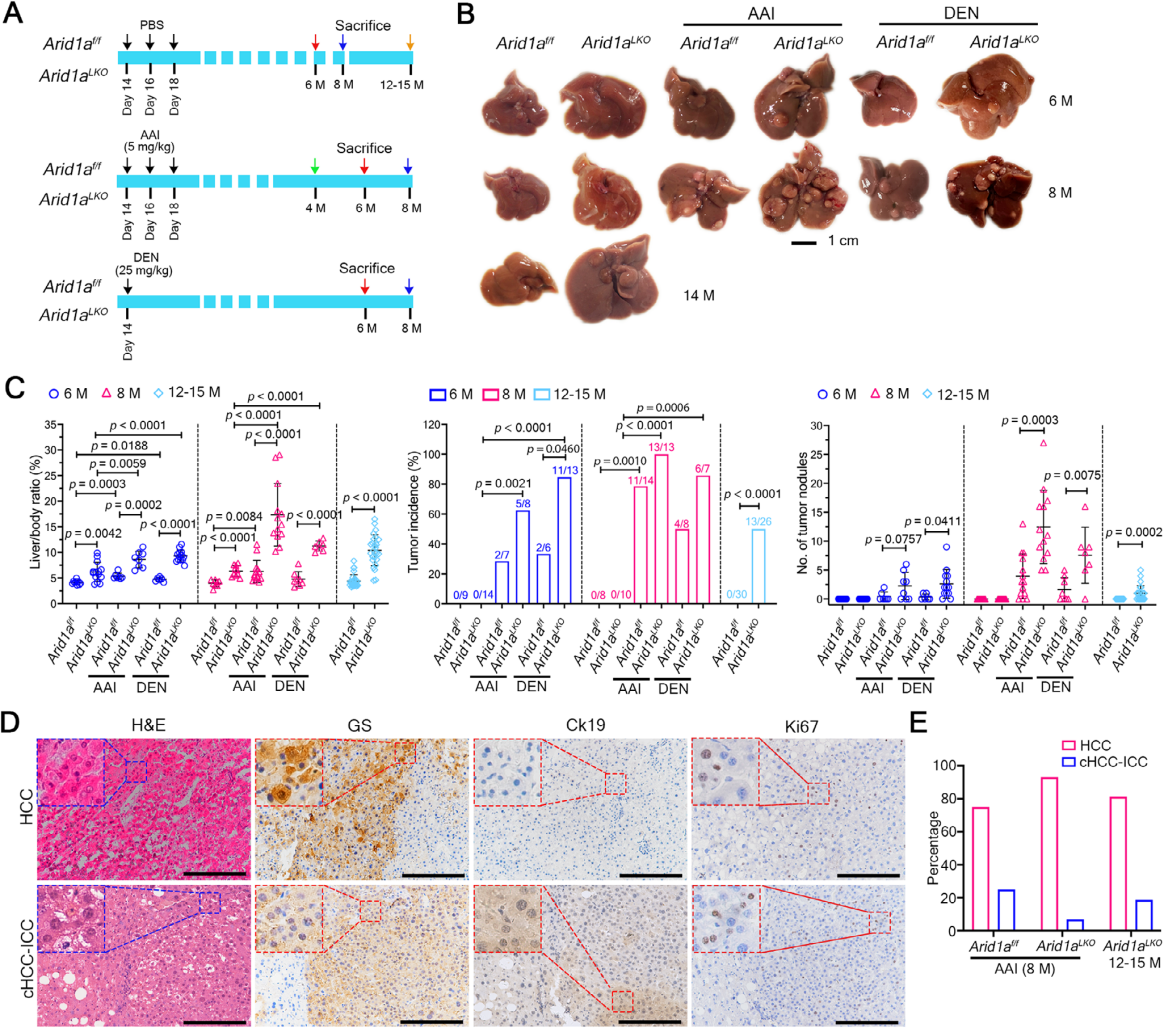
Chemical carcinogens AAI and DEN promote liver tumorigenesis in *Arid1a*‐deficient mice. A) Experiment design for AAI or DEN treatment in mouse models. B) Representative liver images at indicated time points. C) Liver/body weight ratios, tumor incidence, and tumor nodule counts across groups. Sample sizes: *Arid1a^f/f^* (6 months), n = 9; *Arid1a^LKO^* (6 months), n = 14; *Arid1a^f/f^* (8 months), n = 8; *Arid1a^LKO^* (8 months), n = 10; *Arid1a^f/f^* (12–15 months), n = 30; *Arid1a^LKO^* (12–15 months), n = 26; AAI‐treated *Arid1a^f/f^* (6 months), n = 7; AAI‐ treated *Arid1a^LKO^* (6 months), n = 8; AAI‐ treated *Arid1a^f/f^* (8 months), n = 14; AAI‐treated *Arid1a^LKO^* (8 months), n = 13; DEN‐treated *Arid1a^f/f^* (6 months), n = 6; DEN‐treated *Arid1a^LKO^* (6 months), n = 13; DEN‐treated *Arid1a^f/f^* (8 months), n = 8; DEN‐treated *Arid1a^LKO^* (8 months), n = 7. Tumor‐bearing mice/total mice per group are shown in the above histograms. Data are represented as means ± s.d. (left and right panels). *P* values: two‐tailed Student's *t*‐test (left/right panels); Fisher's exact test (middle panel). D) Representative hematoxylin and eosin (H&E) staining and immunohistochemistry images (n ≥ 5) of liver tissue sections from 8‐month‐old AAI‐treated *Arid1a^LKO^* mice. Scale bar, 200 µm. E) Classification of liver tumor types induced by AAI, based on tumor nodule counts: AAI‐treated *Arid1a^f/f^* (8 months), n = 4; AAI‐treated *Arid1a^LKO^* (8 months), n = 44; *Arid1a^LKO^* (12 to 15 months), n = 16.

We hypothesized that exposure to exogenous genotoxic agents, such as AAs, might drive tumorigenesis in the context of *ARID1A* deficiency. To test this, we generated liver‐specific *Arid1a* knockout mice (*Arid1a^LKO^*) by crossing Albumin‐Cre mice with LoxP‐flanked *Arid1a* conditional knockout mice (*Arid1a^f/f^*). We then treated them with AAI^[^ ^23^ ^]^ or diethylnitrosamine (DEN)^[^ ^24^ ^]^ to induce liver cancer (**Figure** **1**A). Notably, AAI or DEN stimulation significantly increased the liver/body weight ratio, tumor incidence, and the number of neoplastic nodules in 6‐ to 8‐month‐old *Arid1a^LKO^* mice compared *Arid1a^f/f^* or PBS‐treated *Arid1a^LKO^* mice. Additionally, *Arid1a^LKO^* mice spontaneously developed an elevated liver/body weight ratio and visible neoplastic nodules by 12–15 months of age (Figure 1B,C).

Histological analysis revealed that these neoplastic nodules lost hepatic lobule structures and exhibited prominent cell proliferation, as shown by Ki67 staining (Figure 1D; Figure S1A,B, Supporting Information). AAI‐induced neoplastic nodules in *Arid1a^f/f^* and *Arid1a^LKO^* mice were primarily HCC (n = 44), with a small number of combined hepatocellular and intrahepatic cholangiocarcinoma (cHCC‐ICC) (n = 4), identified by immunohistochemistry using glutamine synthetase (GS) and cytokeratin (Ck19) as markers of HCC and ICC, respectively (Figure 1D,E). Spontaneous neoplastic nodules in *Arid1a^LKO^* mice were also mainly HCC (n = 13) with a few cHCC‐ICC (n = 3) (Figure S1A, Supporting Information). As expected, the DEN‐induced tumor nodules in all groups were predominantly HCC (n = 20) (Figure S1B, Supporting Information), consistent with our previous report.^[^ ^12^ ^]^ However, no significant differences in liver cancer types were observed among the experimental groups (Figure 1E). These data suggest that exposure to the chemical genotoxic carcinogens AAI and DEN promotes liver cancer development in *Arid1a^LKO^* mice, indicating that genomic DNA damage contributes to tumorigenesis in the context of *Arid1a*‐deficiency.

### 2.2 Featured Mutational Signatures in *Arid1a*‐Deficient Mice

To further investigate how AAI and DEN promote tumorigenesis in *Arid1a*‐deficient mice, we performed low‐coverage whole‐genome sequencing (WGS) on 14 liver tumor samples (A1T, A2T, A3T, SH12T1, SH12T2, SH12T3, SH12T4, SH30T1, SH30T2, SH30T3, SH44T1, SH44T3, SH49T1, and SH49T2), whole‐exome sequencing (WES) on 17 liver tumor samples (A1T, A2T, A3T, SH12T1, SH12T2, SH12T3, SH12T4, SH30T1, SH30T2, SH30T3, SH44T1, SH44T3, SH49T1, SH49T2, SD19T1, SD54T1, and SD56T1) and 9 non‐tumorous liver tissues (A1N, A2N, A3N, SH12N, SH30N, SH44N, SH49N, SH55N, and SH56N) from 12 *Arid1a^LKO^* mice (4‐15 months old). These included PBS‐treated (A1, A2 and A3), AAI‐treated (SH12, SH30, SH44, SH49, SH55, and SH56), and DEN‐treated (SD19, SD54, and SD56) *Arid1a^LKO^* mice. WGS and WES were also performed using spleen or tail tissues from these mice as references to identify somatic mutations or DNA copy number variations (CNVs) in adjacent non‐tumorous or tumor liver tissues. Additionally, we re‐analyzed WES data from three liver tumors and three non‐tumorous liver tissues from the AAI‐treated wild‐type mice from our previous study^[^ ^23^ ^]^ (Table S1, Supporting Information). Immunohistochemistry using GS as an HCC marker was performed on the adjacent non‐tumorous tissues and tumors sequenced by WGS and WES (Figure S2, Supporting Information).

**Figure 2:**
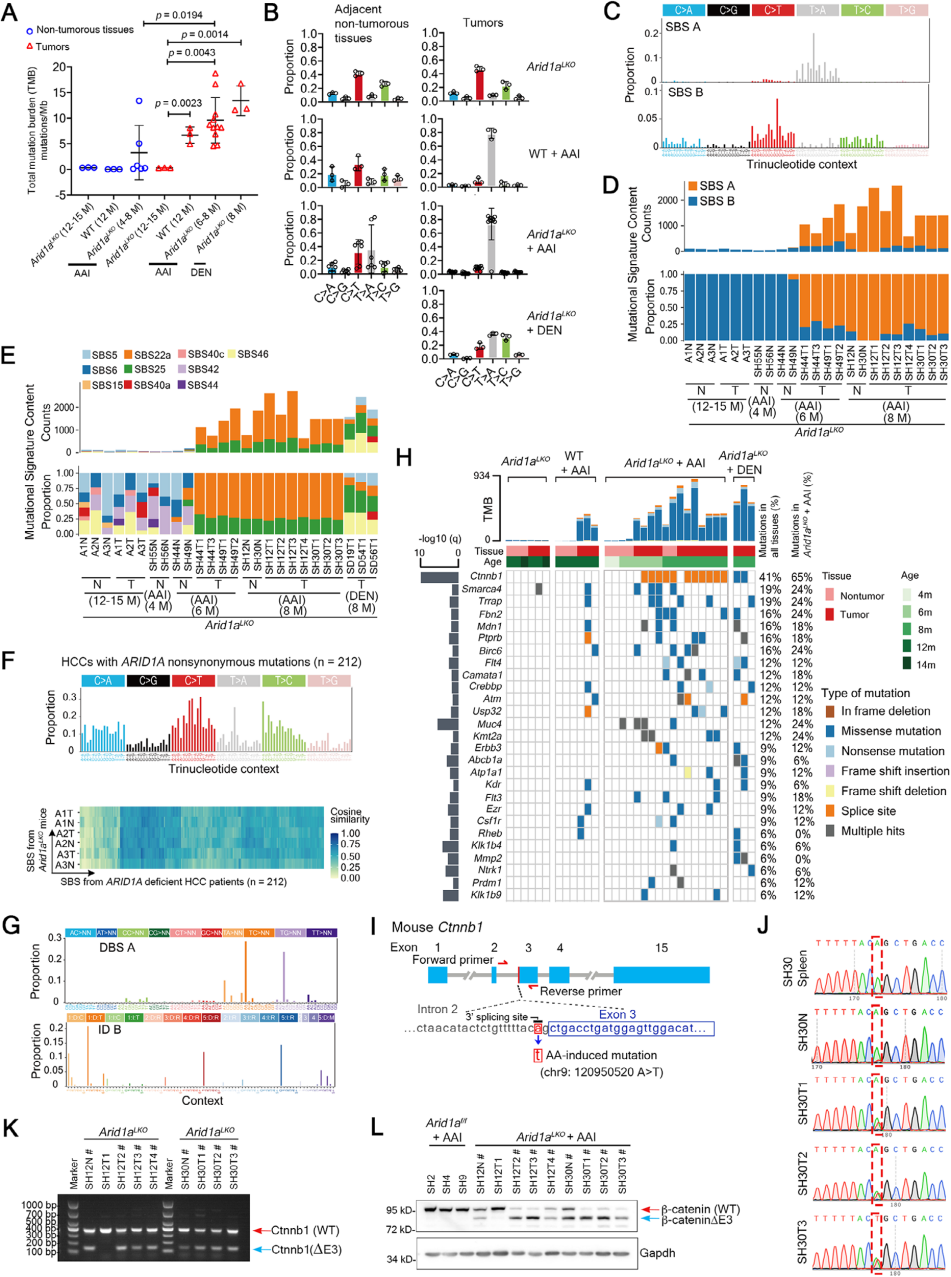
AAI‐induced mutational patterns and the featured *Ctnnb1* mutation in *Arid1a*‐deficient mice. A) Total mutational burden (TMB) in non‐tumorous livers and tumors from *Arid1a^LKO^* and WT mice treated with AAI or DEN, sacrificed at indicated time points. Sample size: *Arid1a^LKO^* (12–15 months), non‐tumors, n = 3; WT (12 months) + AAI, non‐tumors, n = 3; *Arid1a^LKO^* (4–8 months) + AAI, non‐tumors, n = 6; *Arid1a^LKO^* (12‐15 months), tumors, n = 3; WT (12 months) + AAI, tumors, n = 3; *Arid1a^LKO^* (6–8 months) + AAI, tumors, n = 11; *Arid1a^LKO^* (8 months) + DEN, tumors, n = 3. Data are represented as means ± s.d. *P* values by two‐tailed Student's *t*‐test. B) Average SBS proportions in adjacent non‐tumorous liver tissues and tumors from *Arid1a^LKO^* (12‐15 months), AAI‐treated WT (12 months), AAI‐treated *Arid1a^LKO^* (4–8 months), and DEN‐treated *Arid1a^LKO^* mice (8 months). Sample size was the same as in (A). Data are represented as means ± s.d. C) *De novo* SBS signature analysis of 23 WES datasets of adjacent non‐tumorous liver tissues and tumors from *Arid1a^LKO^* and AAI‐treated *Arid1a^LKO^* mice. D) *De novo* SBS signature counts/proportions in adjacent non‐tumorous liver tissues (N) and tumors (T) from *Arid1a^LKO^* and AAI‐treated *Arid1a^LKO^* mice. E) COSMIC SBS signature similarities in N and T from *Arid1a^LKO^*, AAI‐treated, and DEN‐treated *Arid1a^LKO^* mice. F) Average *de novo* SBS signatures from human HCCs (n = 212) with *ARID1A* mutations (top). Cosine similarity between human HCC mutational signatures and *Arid1a^LKO^* murine signatures (bottom). G) Representative *de novo* DBS A (top) and ID B (bottom) signatures in N/T from AAI‐treated WT, *Arid1a^LKO^*, and AAI‐treated *Arid1a^LKO^* mice. H) Mutation frequencies of driver genes in N/T tissues from *Arid1a^LKO^*, AAI‐treated WT, AAI‐treated *Arid1a^LKO^*, and DEN‐treated *Arid1a^LKO^* mice (*q* < 0.1, Pearson's Chi‐squared test). I) Schematic of *Ctnnb1* gene showing the AAI‐induced splicing site mutation. Red arrows: primer locations amplified genomic DNA for Sanger sequencing. J) Sanger sequencing of amplified DNA from spleen (SH30 spleen), non‐tumorous liver (SH30N), and tumors (SH30T1, SH30T2, and SH30T3) of AAI‐treated *Arid1a^LKO^* mice using the primers in (I). Red rectangles: AAI‐induced mutation site. K) RT‐qPCR products amplified with primers in *Ctnnb1* exons 2 and 4. Red arrow, wild‐type Ctnnb1. Blue arrow, mutated Ctnnb1 without exon 3. L) Western blotting of N/T from AAI‐treated *Arid1a^f/f^* and *Arid1a^LKO^* mice. Gapdh: loading control. #: Samples with *Ctnnb1* splicing site mutation identified by WES. Red arrow, wild‐type β‐catenin. Blue arrow, mutated β‐catenin without exon 3.

Low‐coverage WGS analysis revealed genomic CNVs affecting whole chromosomes or large DNA fragments in AAI‐induced and control *Arid1a^LKO^* tumors (Figure S3A, Supporting Information). WES analysis identified 30802 somatic alterations, including 29212 single nucleotide variations (SNVs) and 1590 small insertion‐deletion mutations (INDELs), with 8751 non‐synonymous SNVs, 309 exonic INDELs, and 393 splice site mutations (Tables S1 and S2, Supporting Information). As expected, AAI‐induced tumors in *Arid1a^LKO^* mice had a significantly higher total mutational burden (TMB) (mean 9.59/Mb) compared to adjacent non‐tumorous tissues (mean 3.28/Mb) and control groups. DEN‐treated *Arid1a^LKO^* mice also showed higher TMB than controls (**Figure** **2**A; Table S1, Supporting Information).

Liver tumors from AAI‐treated wild‐type and *Arid1a^LKO^* mice exhibited high T>A transversion frequencies (average 78.4% and 73.5%, respectively). In contrast, the average T>A transversion frequencies in tumors from DEN‐treated and control *Arid1a^LKO^* groups were 36.5% and 8.9%, respectively (Figure 2B; Table S1, Supporting Information). Two representative single base substitution (SBS) signatures, SBS A (0.948 cosine similarity with COSMIC SBS22a) and SBS B, emerged in *Arid1a^LKO^* and AAI‐treated *Arid1a^LKO^* groups (Figure 2C,E). SBS22a dominated in AAI‐treated tumors and adjacent non‐tumorous liver tissues. SBS B has cosine similarities of 0.752, 0.727, 0.722, 0.718, 0.684, 0.654, and 0.633 with COSMIC SBS5, SBS6, SBS42, SBS40c, SBS40a, SBS44, and SBS15, respectively. SBS B was enriched in older *Arid1a^LKO^* mice and some non‐tumorous tissues (SH55N, SH56N, SH44N, and SH49N) (Figure 2D,E), suggesting that mutational signature SBS B could be related to *Arid1a* deficiency. Furthermore, analysis of WES data from HCC samples in the Cancer Genome Atlas Program (TCGA), the International Cancer Genome Consortium (ICGC), cBioPortal, NODE database, and published reports^[^ ^25^, ^26^, ^27^, ^28^, ^29^, ^30^, ^31^, ^32^, ^33^ ^]^ revealed that 212 HCC patients with non‐synonymous *ARID1A* mutations showed a 0.824 cosine similarity with SBS B, characterized by C>T substitutions (31.0%) (Figure 2F).

Doublet base substitution (DBS) and insertion/deletion (ID) signature analysis identified DBS A (0.801 cosine similarity with COSMIC DBS20) and ID B (0.626 cosine similarity with COSMIC ID23), associated with AAI exposure.^[^ ^34^ ^]^ DBS B and ID A were linked to *Arid1a* deficiency (Figure 2G; Figure S3B–E, Supporting Information). DEN‐induced SBS, DBS, and ID signatures differed significantly from those of the *Arid1a^LKO^* group and AAI‐treated wild‐type and *Arid1a^LKO^* groups (Figure 2E; Figure S3F–H, Supporting Information). Additionally, we used the HCC cell line HuH‐7, in which we had constructed *ARID1A* knockout cell line using CRISPR/Cas9 (Figure S4A, Supporting Information). We treated the *ARID1A*‐knockout cells with 25 µm aflatoxin B1 (AFB1) or 10 µm cisplatin for 24 h and then detected DNA damage using the alkaline comet assay, where parent cells were used as control. Interestingly, we found that AFB1 significantly increased DNA damage in *ARID1A*‐deficient cells, while cisplatin induced comparable DNA damage levels in both control and *ARID1A*‐knockout cells (Figure S4B,C, Supporting Information).

In summary, AAI stimulation induced a higher total mutational burden in *Arid1a*‐deficient mice.

### 2.3 AAI Induces *Ctnnb1* Exon 3 Skipping in *Arid1a*‐Deficient Mice

WES analysis identified somatic mutations in key driver genes, including a unique A‐to‐T substitution (chr9:120950520A>T, mm10) at the *Ctnnb1* intron 2/exon 3 boundary in 64.7% (11/17) of AAI‐treated *Arid1a^LKO^* tissues (Figure 2H). This mutation altered the *Ctnnb1* pre‐mRNA 3′ splicing acceptor site, potentially causing exon 3 skipping during mRNA splicing (Figure 2I). We amplified DNA from AAI‐treated *Arid1a^LKO^* liver tissues using primers adjacent to the *Ctnnb1* mutation site and verified the mutation by Sanger sequencing (Figure S5A,B, Supporting Information; Figure 2J). RT‐PCR using mRNAs from these tissues confirmed the loss of the exon 3 coding sequence (Figure 2K; Figure S5C, Supporting Information), corresponding to truncated β‐catenin protein (Figure 2L) in tissues harboring this specific *Ctnnb1* mutation.

Moreover, Sanger sequencing was used to verify DEN‐induced mutations in *Ctnnb1* (Figure S6A, Supporting Information). We found DEN induced non‐synonymous single nucleotide mutations in exon 3 of *Ctnnb1* (p.S33P, p.I35S, p.I35N, p.T41A, and p.S45F) in some *Arid1a^LKO^* liver tumors (Figure S6B, Supporting Information), consistent with previous reports of DEN‐induced mutations in wild‐type mouse livers.^[^ ^24^ ^]^ However, DEN stimulation did not induce mutation at the same nucleotide as AAI‐induced *Ctnnb1* mutation (Figure S6C, Supporting Information). Compared to adjacent non‐tumorous tissues, DEN‐induced *Ctnnb1* mutation resulted in elevated β‐catenin protein levels (Figure S6D, Supporting Information).

RNA‐seq analysis of seven tumors and two adjacent non‐tumorous tissues from 8‐month‐old AAI‐treated *Arid1a^LKO^* mice revealed 327 upregulated and 425 downregulated genes in tumors (Figure S7A and Table S4, Supporting Information). Gene ontology (GO) analysis showed that pathways related to WNT signaling, cell growth, proliferation, and migration were enriched in tumor tissues (Figure S7B, top, Supporting Information), while pathways related to metabolic process were enriched in adjacent non‐tumorous tissues (Figure S7B, bottom, Supporting Information). Gene set enrichment analysis (GSEA) using Hallmark gene sets revealed the significant enrichment of mitotic spindle, G2M checkpoint, and E2F targets in tumors (Figure S7C, Supporting Information).

In summary, we identified a unique *Ctnnb1* mutation causing exon 3 skipping in AAI‐treated *Arid1a^LKO^* mice.

### 2.4 Malignant Clonal Evolution During AAI‐Induced Liver Tumorigenesis

To explore the origins and evolutionary trajectories of malignant clones, we analyzed mutational patterns in multiple tumor nodules and adjacent non‐tumorous tissues from four AAI‐treated *Arid1a^LKO^* mice (SH12, SH30, SH49, and SH44). While the SBS A signature predominated in tumors (Figure 2D), individual nodules from the same mice showed divergent chromosomal CNV patterns (Figure S3A, Supporting Information) and minimal shared SNVs (Figure S8A–D, Supporting Information), indicating independent origins and parallel evolution of AAI‐induced lesions, consistent with chemical carcinogen‐induced tumorigenesis.^[^ ^35^ ^]^

SciClone analysis of variant allele frequencies (VAFs)^[^ ^36^ ^]^ revealed 2–4 subclones in nine of eleven tumor nodules and two of six adjacent non‐tumorous tissues (SH44N and SH49N from 6‐month‐old mice) (Figure S9A,B, Supporting Information).

Given the limited shared mutations across tissues, we comprehensively analyzed raw WES sequencing reads to identify all nonsynonymous mutations. The characteristic *Ctnnb1* splicing mutation was the most frequent alteration, detected in ten of eleven tumors and four of six non‐tumorous tissues from 4‐ to 8‐month‐old AAI‐treated *Arid1a^LKO^* mice (Figure S10A, Supporting Information). Notably, its mutation frequency increased during tumorigenesis in three liver tissues (Figure S10B, Supporting Information), suggesting it drives clonal expansion and malignant transformation in *Arid1a*‐deficient livers. Evolutionary trajectory analysis revealed that the *Ctnnb1* mutation predominated in most malignant subclones, while other driver mutations (*Fat4*, *Herc1*, *Muc4*, and *Wnk2*) appeared sporadically in SH12T1 and SH44T1 subclones (Figure S10C, Supporting Information).

Collectively, our findings demonstrate polyclonal tumor origins in chemical carcinogen‐induced tumorigenesis, with *Ctnnb1* splicing‐mutant clones driving clonal expansion and malignant transformation in AAI‐treated *Arid1a^LKO^* mice.

### 2.5 Single‐Nucleus RNA‐seq (snRNA‐seq) Reveals Altered Cell Types and Communications During Liver Tumorigenesis

**Figure 3:**
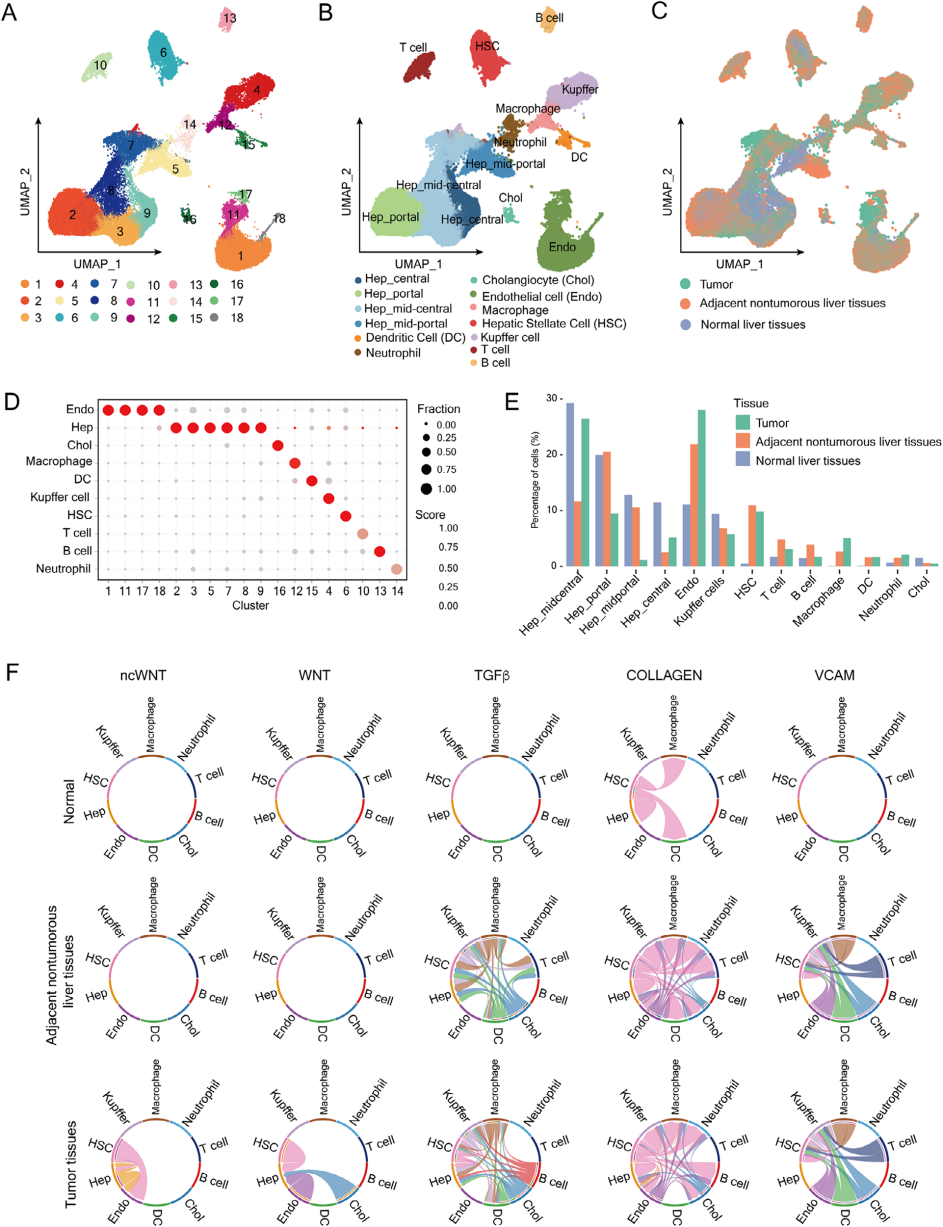
SnRNA‐seq reveals altered cell composition and interactions in AAI‐treated *Arid1a*‐deficient livers. A–C) UMAP plots (Seurat) show cell clusters (A), annotated cell types (B), and tissue origins (C). D) Cell‐lineage marker scores and expression fractions across clusters calculated by Seurat. E) Distribution of cell types and subtypes across tissues, shown as percentages. F) Cell interaction activities of key signaling pathways (Cellchat) in liver tissues from WT mice (top), adjacent non‐tumorous liver tissues (middle), and tumors (bottom) of AAI‐treated *Arid1a^LKO^* mice.

To investigate changes in cell types and states during liver tumorigenesis, we performed snRNA‐seq on paired tumors and adjacent non‐tumorous liver tissues from two 8‐month‐old AAI‐treated *Arid1a^LKO^* mice with *Ctnnb1* splicing mutation (SH12N vs SH12T4 and SH30N vs SH30T1). After quality control and doublet removal, 44794 nuclei were analyzed, detecting 24403 genes (average 8694 unique molecular identifiers (UMIs); median 1997 genes/nucleus; Table S3, Supporting Information). We integrated our data with published snRNA‐seq datasets from normal mouse livers as references,^[^ ^37^, ^38^ ^]^ obtaining 54004 nuclei clustered into 18 groups via uniform manifold approximation and projection (UMAP) (**Figure** **3**A). Using inferCNV (v 1.16.0), we found that cells from tumor tissues (AAI‐treated *Arid1a^LKO^*) showed higher CNVs compared to those from normal (wild‐type) and adjacent non‐tumorous liver tissues (AAI‐treated *Arid1a^LKO^*), indicating that the majority of cells were malignant (Figure S11A, Supporting Information). Based on lineage‐specific markers (Table S3, Supporting Information), these clusters were classified into ten cell types: hepatocytes (Hep, divided into portal, mid‐portal, mid‐central, central subclusters), cholangiocytes (Chol), endothelial cells (Endo), hepatic stellate cells (HSCs), monocyte‐derived macrophages, Kupffer cells (Kupffer), dendritic cells (DCs), neutrophils, T cells, and B cells (Figure 3B–D).^[^ ^38^ ^]^

Notably, the proportions of immune cells (macrophages, Kupffer cells, DCs, neutrophils, T cells, B cells), endothelial cells, and HSCs increased significantly in both tumors and adjacent non‐tumorous tissues, suggesting AAI‐induced immune and non‐immune responses reshaped the tumor microenvironment.^[^ ^39^, ^40^, ^41^ ^]^ Conversely, the percentage of central hepatocytes decreased in both tumor and adjacent non‐tumorous tissues compared to normal livers (Figure 3E).

CellphoneDB^[^ ^42^ ^]^ analysis revealed enhanced cell‐cell interactions in tumors and adjacent non‐tumorous liver tissues from AAI‐treated *Arid1a^LKO^* mice compared to normal liver tissues (Figure S11B, Supporting Information). Further analysis using the Cellchat algorithm^[^ ^43^ ^]^ identified upregulated pathways, including Wnt, non‐canonical Wnt (ncWnt), Tgfβ, Collagen, Vcam, Fn1, Galectin, Laminin, Mif, Visfatin, Vtn, and Complement, relative to normal livers (Figure 3F; Figure S11C, Supporting Information).

Together, these findings highlight aberrant cell types, states, and cell‐cell interactions in *Arid1a*‐deficient liver under AAI stimulation, potentially driving inflammation, fibrogenesis, and tumor progression.

### 2.6 Malignant Origin of Cells Inferred from Single‐Cell Analysis

**Figure 4:**
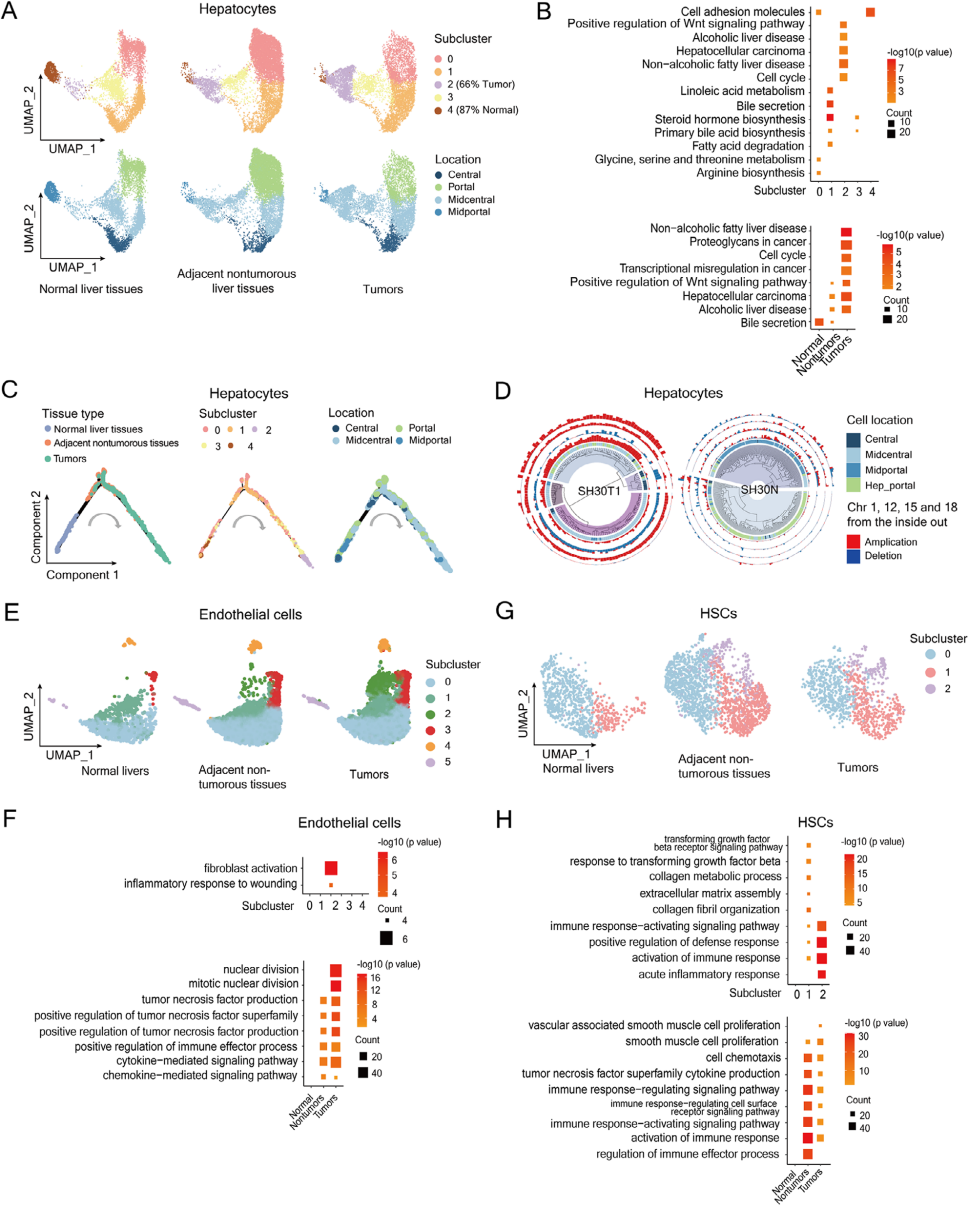
Subcluster analysis of hepatocytes, endothelial cells, and hepatic stellate cells. A) UMAP plots show hepatocyte subclusters (top) and their lobule locations (bottom) in normal liver tissues of WT mice (left), adjacent non‐tumorous liver tissues (middle), and tumors of AAI‐treated *Arid1a^LKO^* mice (right). B) KEGG analysis of enriched signaling pathways in hepatocyte subclusters (top) and tissues (bottom) from (A). C) Pseudotime trajectory analysis of hepatocytes based on tissue origin, subcluster, and lobule location. D) CNVs and clonal inference in 1000 hepatocytes from tumor (SH30T1, left) and adjacent non‐tumorous tissue (SH30N, right) of AAI‐treated *Arid1a^LKO^* mouse. Circle diagrams (inside to outside): cell clones, cell types, and chromosomal CNVs. E) UMAP plots of endothelial cell subclusters in normal liver tissues of WT mice (left), adjacent non‐tumorous liver tissues (middle), and tumors (right) of AAI‐treated *Arid1a^LKO^* mice. F) GO analysis of pathways enriched in endothelial cell subclusters (top) and tissue origins (bottom) from (E). G) UMAP plots of hepatic stellate cell (HSC) subclusters in normal liver tissues of WT mice (left), adjacent non‐tumorous liver tissues (middle), and liver tumors (right) of AAI‐treated *Arid1a^LKO^* mice. H) GO analysis of pathways enriched in HSCs based on subclusters (top) and tissue origin (bottom) indicated from (G).

To investigate the malignant origin of cells in AAI‐induced liver cancer in *Arid1a*‐deficient mice, we identified five hepatocyte subclusters using snRNA‐seq analysis. Notably, subcluster 4 (primarily mid‐portal hepatocytes) was enriched in normal livers and decreased in adjacent non‐tumorous tissues and tumors, while subcluster 2 (mainly mid‐central hepatocytes) increased in adjacent non‐tumorous tissues and tumors (**Figure** **4**A). Kyoto Encyclopedia of Genes and Genomes (KEGG) analysis showed subcluster 2 was enriched in pathways related to cell cycle, liver disease, HCC, and WNT signaling (Figure 4B, top). Furthermore, based on the tissue origin, pathways associated with liver diseases, HCC, cell cycle, proteoglycans, transcriptional dysregulation, and WNT signaling were predominantly enriched in tumors (Figure 4B, bottom).

Pseudo‐time trajectory analysis revealed a transformation pathway from normal cells to tumors, suggesting malignancy likely originated from subclusters 0 or 1 (portal or mid‐portal hepatocytes) in non‐tumorous livers, differentiating into subclusters 2 and 3 (mid‐central hepatocytes) enriched in tumors (Figure 4C).

CNVs inferred from snRNA‐seq of hepatocytes in SH12T4 and SH30T1 tumors were generally consistent with WGS results (Figure 4D; Figures S3A and S12A, Supporting Information). Genomic DNA amplification of chromosomes 1, 15, and 18 in SH30T1 was common in malignant hepatocytes and also detected in some hepatocytes from the adjacent non‐tumorous liver SH30N. Malignant hepatocytes in SH30T1 were broadly divided into two main clusters (Figure 4D). CNVs in SH12T4 were relatively rare, though some hepatocytes from non‐tumorous liver tissue SH12N exhibited similar chromosomal aberrations as tumors SH12T1, SH12T3, and SH12T4 (Figures S3A and S12A, Supporting Information).

These findings suggest that malignant transformation may arise from mid‐central (zone 2) hepatocytes in non‐tumorous livers.

### 2.7 Tumor Microenvironment at Single‐Cell Resolution

To analyze the tumor microenvironment, we performed subcluster analysis on the main cell types in the liver besides hepatocytes. Among these cells, neutrophils, macrophages, Kupffer cells, DCs, T cells, and B cells increased in both adjacent non‐tumorous and tumor tissues compared to normal livers (Figure 4E,G; Figure S12B, Supporting Information). As the important nonparenchymal cells in the liver, subclusters of endothelial cells and HSCs differentially enriched in normal, adjacent non‐tumorous, or tumor livers. Subcluster 2 of endothelial cells was specifically enriched in tumors, while subcluster 3 comprised cells from both tumor and adjacent non‐tumorous tissues (Figure 4E). Pathways related to inflammatory response to wound healing and fibroblast activation were enriched in endothelial subcluster 2 (Figure 4F, top). GO analysis revealed enrichment of tumor necrosis factor (TNF) production, immune effectors, and cytokine/chemokine‐mediated signaling in both tumors and adjacent non‐tumor tissues (Figure 4F, bottom).

Subclusters 1 and 2 of HSCs increased significantly in both tumors and adjacent non‐tumor tissues (Figure 4G). Subcluster 1 of HSCs was enriched in TGFβ pathway, collagen metabolism, extracellular matrix, and immune activation, while subcluster 2 was associated with immune activation and inflammatory response (Figure 4H, top). GO analysis revealed elevated expression of genes involved in vascular smooth muscle cell proliferation, TNF superfamily cytokine production, and immune response in HSCs from both tumors and adjacent non‐tumorous tissues (Figure 4H, bottom).

In summary, our results highlight aberrant activation of endothelial cells and HSCs in adjacent non‐tumorous and tumor tissues of livers from AAI‐treated *Arid1a^LKO^* mice, which may contribute to the liver inflammation, fibrosis, and tumorigenesis.

### 2.8 *Arid1a* Deficiency Represses Critical Genes Related to NER

**Figure 5:**
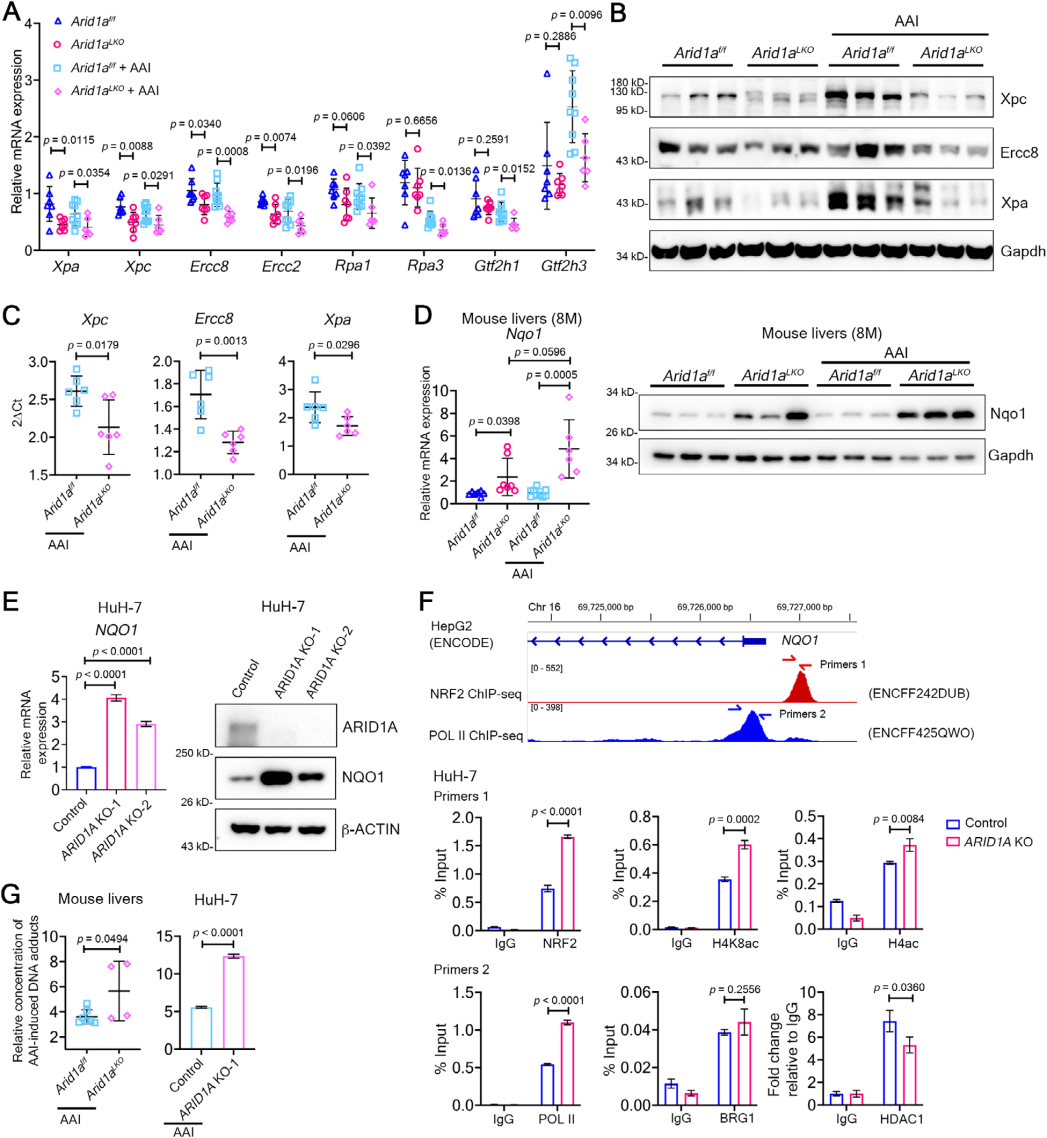
*Arid1a* deficiency impairs nucleotide excision repair. A) RT‐qPCR of key NER genes in non‐tumorous liver tissues from *Arid1a^f/f^* (n = 7), *Arid1a^LKO^* (n = 7), AAI‐treated *Arid1a^f/f^* (n = 9), and AAI‐treated *Arid1a^LKO^* (n = 6) mice. Data are mean ± s.d. from three individual experiments. *P* values: two‐tailed Student's *t*‐test. B) Western blotting of non‐tumorous liver tissues from 8‐month‐old mice using indicated antibodies. Gapdh served as a loading control. C) DNase I sensitivity assessed by real‐time PCR using 2^∆Ct^ in liver tissues from AAI‐treated 8‐month‐old mice of *Arid1a^f/f^* (n = 6) and *Arid1a^LKO^* (n = 6). Primers targeted regions indicated in Figure S14A (Supporting Information). Data are mean ± s.d. *P* values were calculated using a two‐tailed Student's *t*‐test. D) RT‐qPCR of *Nqo1* mRNA (left) in the non‐tumorous liver tissues from 8‐month‐old mice of *Arid1a^f/f^* (n = 7), *Arid1a^LKO^* (n = 7), AAI‐treated *Arid1a^f/f^* (n = 9), and AAI‐treated *Arid1a^LKO^* (n = 6). Data are mean ± s.d. from indicated mice. *P* values (left): two‐tailed Student's *t*‐test. Western blotting (right) of non‐tumorous liver tissues from 8‐month‐old mice of the indicated groups using corresponding antibodies. Gapdh served as a loading control. E) RT‐qPCR of *NQO1* mRNA in HuH‐7 cells (left) and Western blotting (right) from 8‐month‐old mice. Data are mean ± s.d. from three individual experiments. *P* values (left): two‐tailed Student's *t*‐test. β‐ACTIN was served as a loading control. F) ChIP‐seq data of NRF2 and POL II in HepG2 cells (top; ENCODE). Arrows: primer locations for PCR. ChIP‐qPCR assays for NRF2, BRG1, H4K8ac, H4ac, HDAC1, and POL II at the *NQO1* promoter in HuH‐7 cells. Data are mean ± s.d. from three individual experiments. *P* values: two‐tailed Student's *t*‐test. G) UPLC‐TQ/MS detection of AAI‐induced DNA adducts in liver tissues from AAI‐treated *Arid1a^f/f^* (n = 7) and *Arid1a^LKO^* (n = 4) mice (left), and in HuH‐7 cells (right). Data are mean ± s.d. *P* values: two‐tailed Student's *t*‐test.

In our study, AAI treatment increased TMB in *Arid1a*‐deficient adjacent non‐tumorous tissues (Figure S13A, Supporting Information). Since AAI‐induced DNA adducts are primarily repaired by the NER system,^[^ ^20^ ^]^ including transcription‐coupled NER (TC‐NER) and global genomic NER (GG‐NER),^[^ ^44^ ^]^ we hypothesized that *ARID1A* loss might downregulate NER‐related genes. Analysis of RNA‐seq data from TCGA HCC cohorts revealed a positive correlation between *ARID1A* expression and transcription of some NER‐related genes (Figure S13B, Supporting Information). In *Arid1a*‐deficient mice, transcription of *Xpa*, *Xpc*, and *Ercc2/8* was significantly downregulated regardless of AAI stimulation, while *Gtf2h1/3* and *Rpa1/3* were reduced only with AAI treatment (**Figure** **5**A). Consistent with these findings, protein levels of Xpa, Xpc, and Ercc8 were also reduced in *Arid1a*‐deficient livers (Figure 5B).

To investigate the mechanism underlying transcriptional repression of these NER‐related genes, we analyzed chromatin accessibility using DNase‐seq datasets of mouse livers from the Encyclopedia of DNA elements (ENCODE, accession number ENCFF100XUL, ENCFF178LXB, and ENCFF445QMM). This revealed DNase I hypersensitive sites at the promoters of *Xpa*, *Xpc*, and *Ercc8* (Figure S14A, Supporting Information). DNase I treatment showed reduced chromatin accessibility at these promoters in non‐tumorous liver tissues from AAI‐treated *Arid1a^LKO^* mice compared to *Arid1a^f/f^* controls, indicating that *Arid1a* deficiency suppresses NER‐related gene expression by limiting chromatin accessibility at their promoters (Figure 5C).

Notably, we did not identify a clear DNase I hypersensitive site around the specific splicing region of *Ctnnb1* (Chr9:120950520) using the abovementioned ENCODE DNase‐seq data (Figure S14B, left, Supporting Information), and *Arid1a* loss did not alter DNase I sensitivity at this site in our mouse liver tissues (Figure S14B, right, Supporting Information), suggesting that the AAI‐induced *Ctnnb1* splicing site mutation in *Arid1a*‐deficient mice is not driven by chromatin remodeling at this locus.

### 2.9 *Arid1a* Deficiency Enhances Transcription of *Nqo1* Responsible for AA Bioactivation

Nqo1 is the primary enzyme for AAI bioactivation^[^ ^45^ ^]^ and DNA adduct formation in vivo.^[^ ^46^ ^]^ Here, we found that *Arid1a* loss significantly increased *Nqo1* transcription and protein expression in 4‐, 6‐, and 8‐month‐old mice, regardless of AAI treatment (Figure 5D; Figure S14C, Supporting Information). Immunohistochemistry confirmed elevated Nqo1 expression in *Arid1a^LKO^* livers compared to *Arid1a^f/f^* mice (Figure S14D, Supporting Information). Knockout of *ARID1A* in the human HCC HuH‐7 cells using CRISPR/Cas9 also upregulated *NQO1* mRNA and protein levels (Figure 5E).

We found that *Arid1a* deficiency did not alter the chromatin accessibility at the *Nqo1* promoter (Figure S14E, Supporting Information), nor did BRG1/BRM inhibitors (BRM014, ACBI1, and AU‐15330) affect NQO1 expression (Figure S14F, Supporting Information). As *NQO1* expression is regulated by the transcription factor NRF2,^[^ ^47^ ^]^ we performed ChIP assays and observed significantly increased NRF2 occupancy on the *NQO1* promoter in the absence of *ARID1A* (Figure 5F). Although the BAF complex primarily activates genes by regulating chromatin accessibility near enhancers, it can also repress transcription by recruiting histone deacetylation complexes.^[^ ^48^, ^49^, ^50^ ^]^ Consistent with this, we found increased RNA‐polymerase II binding adjacent to the NRF2 binding site on the *NQO1* promoter in *ARID1A*‐deficient cells (Figure 5F). Furthermore, reduced HDAC1 binding and increased histone H4 acetylation (lysines 5, 8, 12, and 16) near the *NQO1* promoter indicated decreased recruitment of histone deacetylation complexes in the absence of *ARID1A* (Figure 5F).

We also found that acute AAI treatment significantly increased AAI‐induced DNA adduct level in *ARID1A* knockout HuH‐7 cells and *Arid1a^LKO^* mouse livers (Figure 5G), consistent with NQO1 upregulation, suggesting that *ARID1A* loss increases susceptibility to AAI‐induced DNA damage and liver tumorigenesis.

Moreover, we found that overexpression of *XPC* or *XPA* in *ARID1A*‐knockout HuH‐7 cells could alleviate AAI‐induced DNA damage (Figure S15A,B, Supporting Information). Whereas reducing the level of NQO1 using CRISPR/Cas9 system inhibited cell proliferation in both control and *ARID1A*‐knockout HuH‐7 cells (Figure S15C,D, Supporting Information). Additionally, the reduced NQO1 also significantly mitigated DNA damage in *ARID1A*‐knockout cells as exposed to AAI for 48 h (Figure S15E, Supporting Information).

To further explore whether *Arid1a* loss alters global chromatin accessibility, we performed ATAC‐seq assay using adjacent non‐tumorous liver tissues from 8‐month‐old AAI‐treated mice of *Arid1a^f/f^* (n = 6) and *Arid1a^LKO^* (n = 6). We found that *Arid1a* deficiency resulted in 26549 increased sites and 13401 decreased sites (*p* < 0.05) (Figure S16A, Supporting Information). Our results suggest that loss of *Arid1a* changes the global chromatin accessibility, which is consistent with the previous report.^[^ ^51^ ^]^ GO analysis of the enriched ATAC‐seq peaks in *Arid1a^f/f^* mice was related to metabolism, including fatty acid, small molecule, steroid and lipid metabolism, cell response to xenobiotics, DNA damage, and repair (Figure S16B, top, Supporting Information). These decreased chromatin accessibility due to *Arid1a* loss may contribute to the impaired metabolic function of the liver and DNA damage repair system, which may explain the susceptibility of *Arid1a*‐deficient mice or cells to the DNA damage reagents. Furthermore, GO analysis reveals that the enriched ATAC‐seq peaks in *Arid1a^LKO^* mice were related to activation of immune systems, Wnt signaling, and positive regulation of cell growth (Figure S16B, bottom, Supporting Information), which may indicate the activated cell response to AAI‐induced damage. The enriched ATAC‐seq peaks related to Wnt signaling pathway and cell growth in *Arid1a^LKO^* mice are consistent with the activated mutation of *Ctnnb1*. Moreover, we have analyzed ATAC‐seq data performed in hepatocytes isolated from liver tissues of wild‐type and *Arid1a*‐knockout mice from GEO database with the accession number GSE111499.^[^ ^51^ ^]^ Consistent with our results, GO analysis of the enriched ATAC‐seq peaks in wild‐type hepatocytes were related to the function of liver metabolism, DNA damage response, and repair systems (Figure S16C, top, Supporting Information), whereas the enriched ATAC‐seq peaks in *Arid1a*‐knockout hepatocytes were related to cell adhesion and Wnt signaling pathway (Figure S16C, bottom, Supporting Information). These results indicate that *Arid1a* deficiency alters the chromatin accessibility globally and may affect these signaling pathways related to metabolism, DNA damage response and repair, immune responses, Wnt signaling pathway, and so on.

### 2.10 *ARID1A* Loss and *CTNNB1* Activation Synergistically Promote Tumor Development

**Figure 6:**
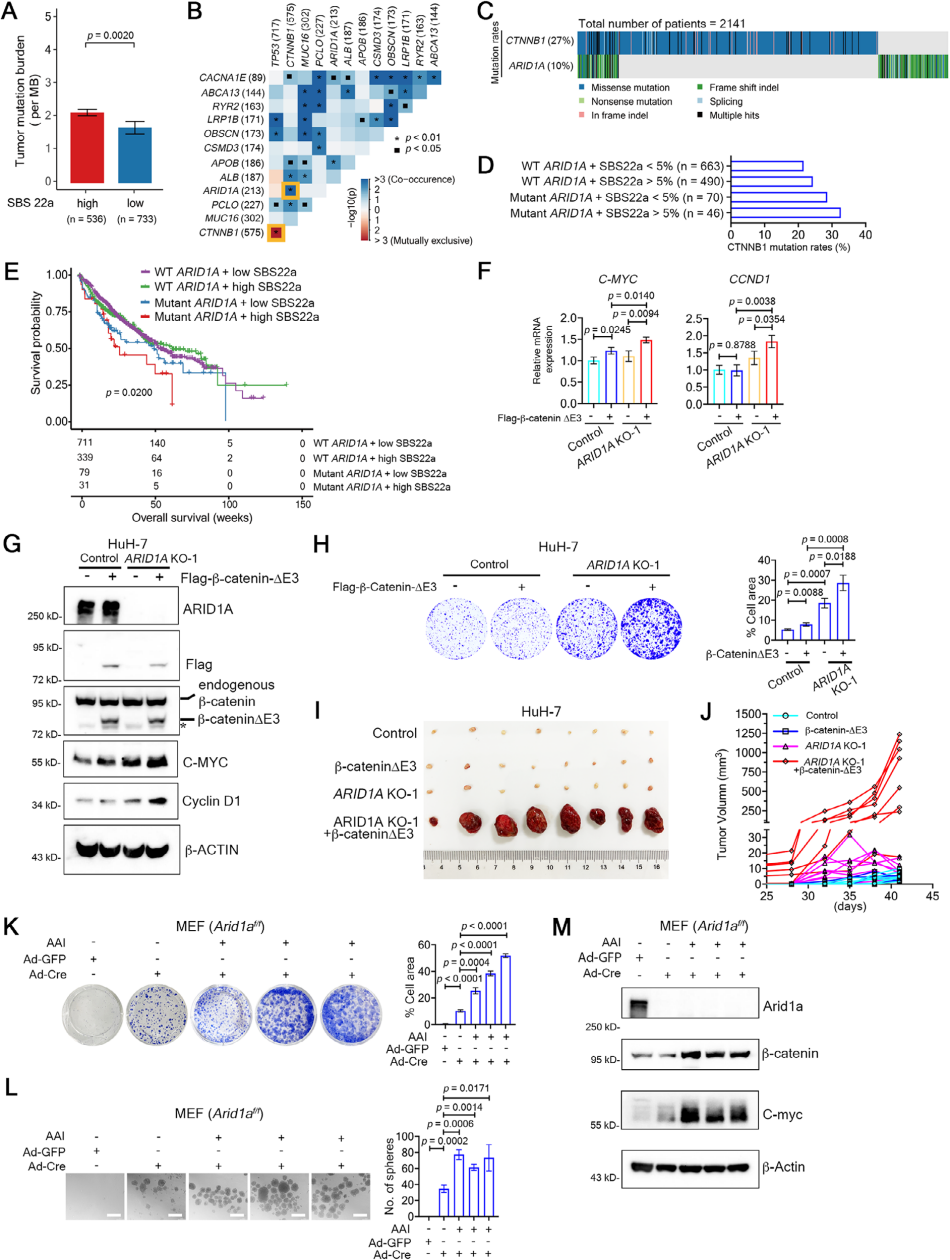
*ARID1A* loss and *CTNNB1* activation synergistically promote tumor development. A) TMB in HCC patients with high (upper quartile, n = 536) and low (lower quartile, n = 733) AA‐induced mutational signature (SBS22a). Data are represented as means ± SEM. *P* value: two‐tailed Student's *t*‐test. B) Driver gene co‐mutations and mutual exclusivity. Numbers: patients with mutations. *P* values: Fisher's exact test. C) Distribution of *CTNNB1* and *ARID1A* co‐mutations in HCC patients from the TCGA, ICGC, and published datasets. D) *CTNNB1* mutation rates in HCC patients from (C), categorized by *ARID1A* mutations and COSMIC SBS22a levels. E) Survival curves of HCC patients stratified by *ARID1A* mutations and SBS22a levels. *P* value: Log‐rank test. F) RT‐qPCR of *C‐MYC* and *CCND1* mRNA in HuH‐7 cells (control and *ARID1A* KO) infected with lentivirus expressing Flag‐β‐catenin‐ΔE3. Data are mean ± s.d. from three replicates. *P* values: two‐tailed Student's *t*‐test. G) Western blotting of HuH‐7 cells from (F). β‐ACTIN served as a loading control. *, unspecific band. H) Cell colony formation assay in HuH‐7 cells from (G) (left). Cell colonies were stained with crystal violet, and their area was calculated using Image J (right panel). Data are mean ± s.d. from three replicates. *P* values: two‐tailed Student's *t*‐test. I) Xenograft tumors in nude mice (n = 8 per group) using HuH‐7 cells from (F). J) Tumor volumes measured every 3 days after palpable tumors appeared in nude mice (n = 8 per group). K) Cell colony formation assay in MEFs (*Arid1a^f/f^*) mice treated with AAI, Ad‐GFP, and Ad‐Cre. Cell colonies stained with crystal violet (left), and their areas were quantified using Image J (n = 3) (right). Data are mean ± s.d. from three replicates. *P* values: two‐tailed Student's *t*‐test. L) Spheroid formation assay assessing self‐renewal of MEFs (*Arid1a^f/f^*) from (K) (n = 3) (left). Sphere numbers quantified (right). Data are mean ± s.d. from three replicates. *P* values: two‐tailed Student's *t*‐test. Scale bar, 100 µm. M) Western blotting of MEFs (*Arid1a^f/f^*) using the indicated antibodies. β‐Actin served as a loading control.

To investigate whether *ARID1A* loss and β‐catenin activation synergistically drive liver cancer, we analyzed the co‐occurrence of *ARID1A* and *CTNNB1* mutations in AA‐exposed human HCC samples. Using COSMIC SBS patterns, we reconstructed somatic mutational signatures in 2141 human HCC samples from TCGA, ICGC, and published datasets,^[^ ^25^, ^26^, ^27^, ^28^, ^29^, ^30^, ^31^, ^32^, ^33^ ^]^ categorizing them by SBS22a signature composition. As expected, HCCs with SBS22a signature in the upper quartile had significantly higher TMB than those in the lowest quartile (2.09/Mb vs 1.57/Mb, *p* = 0.002) (**Figure** **6**A). Notably, among 213 *ARID1A*‐mutated and 575 *CTNNB1*‐mutated HCC patients, 79 (11%) exhibited co‐mutations (*p* < 0.01) (Figure 6B,C), while *CTNNB1* mutations were mutually exclusive with *TP53* mutations (n = 717) (Figure 6B). Patients with *ARID1A* mutations and SBS22a signature > 5% had the highest *CTNNB1* mutation rate (Figure 6D). Prognostic analysis revealed that patients with *ARID1A* mutations and SBS22a signature in the upper quartile had the shortest overall survival (Figure 6E), as did those with *ARID1A* and *CTNNB1* co‐mutations (Figure S17A, Supporting Information). These findings suggest that AA exposure drives co‐occurrence of *ARID1A* loss‐of‐function and *CTNNB1* gain‐of‐function mutations, synergistically promoting tumorigenesis.

To validate this, we transfected *ARID1A* knockout HuH‐7 cells with a vector expressing β‐catenin lacking exon 3 (Flag‐β‐catenin‐∆E3). Combining *ARID1A* loss and active β‐catenin significantly upregulated β‐catenin target genes *C‐MYC* and *CCND1* (Figure 6F,G) and enhanced cell colony formation (Figure 6H). In xenografts, β‐catenin‐∆E3 dramatically accelerated tumor growth in *ARID1A*‐deficient cells (Figure 6I,J).

We further tested this synergy in *Arid1a^f/f^* mouse embryonic fibroblasts (MEFs). Adenovirus expressing Cre recombinase (Ad‐Cre) induced *Arid1a* knockout in MEFs (MEFs*^Arid1a KO^*), while GFP‐expressing adenovirus (Ad‐GFP) served as control. Under our conditions, MEFs*^Arid1a KO^* survived and proliferated at lower densities, and all three independent AAI‐treated MEFs*^Arid1a KO^* cell lines grew faster (Figure 6K) and exhibited higher self‐renewal ability (Figure 6L), compared to wild‐type MEFs and untreated MEFs*^Arid1a KO^*. Interestingly, AAI treatment increased β‐catenin and its downstream target C‐myc protein levels in MEFs*^Arid1a KO^* (Figure 6M), despite the absence of *Ctnnb1* splicing site mutation (data not shown). Hydrodynamic injection of β‐catenin‐∆E3 into *Arid1a^LKO^* mice significantly increased the liver/body weight ratio, total tumor nodules, and large tumors (>3 mm). However, β‐catenin‐∆E3 alone failed to induce liver tumors in control *Arid1a^f/f^* mice (Figure S17B, Supporting Information), consistent with a previous report.^[^ ^52^ ^]^

In summary, *ARID1A* loss and *CTNNB1* activation synergistically promote liver cancer by enhancing β‐catenin signaling and tumorigenic potential, driven by AA‐induced mutational co‐occurrence.

### 2.11 β‐Catenin Inhibition Reduces Liver Tumorigenesis in AAI‐treated *Arid1a*‐Deficient Mice

**Figure 7:**
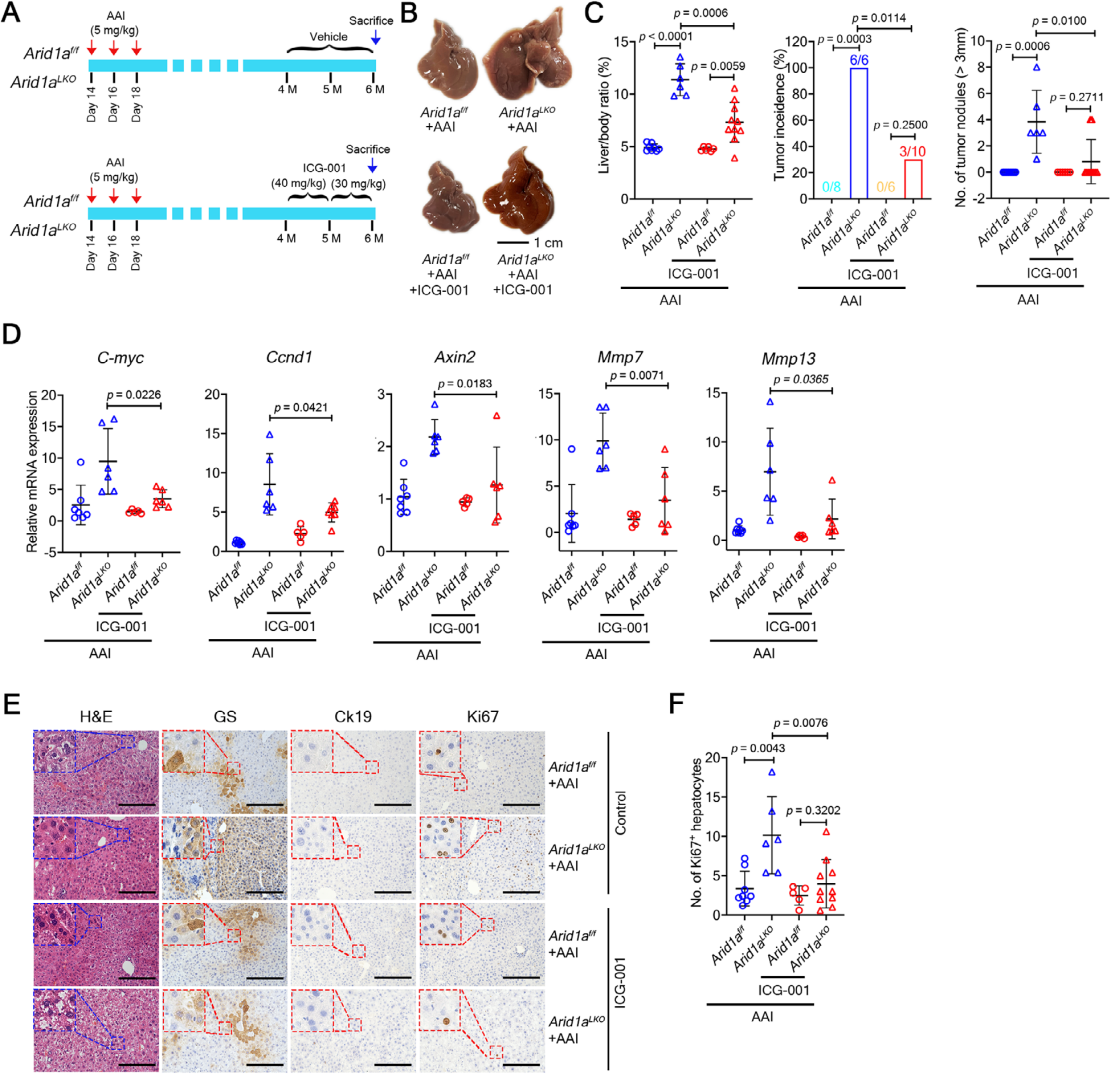
β‐catenin/CBP interaction antagonist reduces liver tumorigenesis in AAI‐treated *Arid1a*‐deficient mice. A) Schematic of mouse models treated with β‐catenin/CBP interaction antagonist ICG‐001. B) Representative liver images from mice in (A). Scale bar, 1 cm. C) Liver/body ratio, tumor incidence, and number of tumor nodules (>3 mm) in AAI‐treated *Arid1a^f/f^* (n = 8), AAI‐treated *Arid1a^LKO^* (n = 6), AAI and ICG‐001‐treated *Arid1a^f/f^* (n = 6), AAI and ICG‐001‐treated *Arid1a^LKO^* mice (n = 10). Numbers above the histograms (middle): tumor‐bearing mice/total mice. Data are represented as means ± s.d. (left and right panels). *P* values: two‐tailed Student's *t*‐test (left and right panels); Fisher's exact test (middle). D) RT‐qPCR of *C‐myc*, *Ccnd1*, *Axin2*, *Mmp7*, *and Mmp13* in the non‐tumorous liver tissues from AAI‐treated *Arid1a^f/f^* (n = 7), AAI‐treated *Arid1a^LKO^* (n = 6), AAI and ICG‐001‐treated *Arid1a^f/f^* (n = 5), and AAI and ICG‐001‐treated *Arid1a^LKO^* (n = 6) mice. Data are represented as means ± s.d. *P* values: two‐tailed Student's *t*‐test. E) Representative immunohistochemistry images of liver tissue sections from indicated groups. Scale bar, 200 µm. F) Average number of Ki67‐positive hepatocytes per field in adjacent non‐tumorous liver tissues from AAI‐treated *Arid1a^f/f^* (n = 8), AAI‐treated *Arid1a^LKO^* (n = 6), AAI and ICG‐001‐treated *Arid1a^f/f^* (n = 6), and AAI and ICG‐001‐treated *Arid1a^LKO^* (n = 10). *P* values: two‐tailed Student's *t*‐test.

Our findings suggest that activated β‐catenin is crucial for HCC initiation in *ARID1A*‐deficient mice. To test this hypothesis, AAI‐treated *Arid1a^LKO^* mice were given either vehicle or ICG‐001 (**Figure** **7**A), a small molecule that specifically disrupts β‐catenin/CBP interaction and thereby inhibits β‐catenin/TCF target gene transcription.^[^ ^53^ ^]^ After two months of treatment, ICG‐001 significantly reduced liver/body weight ratio, tumor incidence, and the number of large tumor nodules (> 3 mm) in AAI‐treated *Arid1a^LKO^* mice compared to vehicle‐treated controls (Figure 7B,C). ICG‐001 also significantly attenuated the elevated expression of β‐catenin target genes *C‐myc*, *Ccnd1*, *Axin2*, *Mmp7*, and *Mmp13* but not *Lgr5* in non‐tumorous livers of AAI‐treated *Arid1a^LKO^* mice (Figure 7D; Figure S18, Supporting Information). Histological analysis revealed that ICG‐001 markedly suppressed cell proliferation, as indicated by reduced Ki67 staining (Figure 7E,F).

## 3 Discussion

Our study revealed that the genotoxic carcinogen AA accelerates liver tumorigenesis in the context of *ARID1A* deficiency, accompanied by a characteristic *Ctnnb1* mutation and alterations in the liver microenvironment. Mechanically, *ARID1A* deficiency repressed key genes involved in NER by reducing chromatin accessibility at their promoters. Loss of *ARID1A* upregulated *NQO1*, a critical enzyme for AA bioactivation, by reducing HDAC1 recruitment and increasing histone acetylation and NRF2 binding to the *NQO1* promoter.

*ARID1A*, encoding the most frequently mutated subunit of the SWI/SNF complex across cancer types, is frequently mutated in normal and non‐tumorous tissues, including morphologically normal liver and chronic liver diseases. In liver cancer, *ARID1A* mutations occur in all three primary types (HCC, ICC, and cHCC‐ICC) and are associated with clinical characteristics such as tumor size,^[^ ^54^ ^]^ metastasis, and poor prognosis.^[^ ^55^ ^]^ As many mutations lead to *ARID1A* loss of function, ARID1A is generally considered to be a tumor suppressor. These inactivating mutations are also found in non‐tumorous liver tissues, potentially increasing hepatic clonal fitness and expansion and promoting regeneration in chronic liver disease without initiating tumorigenesis.^[^ ^8^ ^]^ Thus, *ARID1A* inactivating mutations may be necessary but not always sufficient for initiating tumorigenesis.^[^ ^56^ ^]^

Environmental carcinogens may activate cells harboring *ARID1A* mutations through mutagenic and non‐mutagenic mechanisms to promote early tumorigenesis. This led us to hypothesize that specific environmental factors, such as AA, could contribute to tumorigenesis in the context of *ARID1A* deficiency. Given the frequent presence of the AA‐induced mutational signature SBS22a in HCC patients and the widespread use of AA‐containing herbal medicine in Asia, we investigated whether AA could initiate and promote liver cancer in the context of *ARID1A* loss.

**Figure 8:**
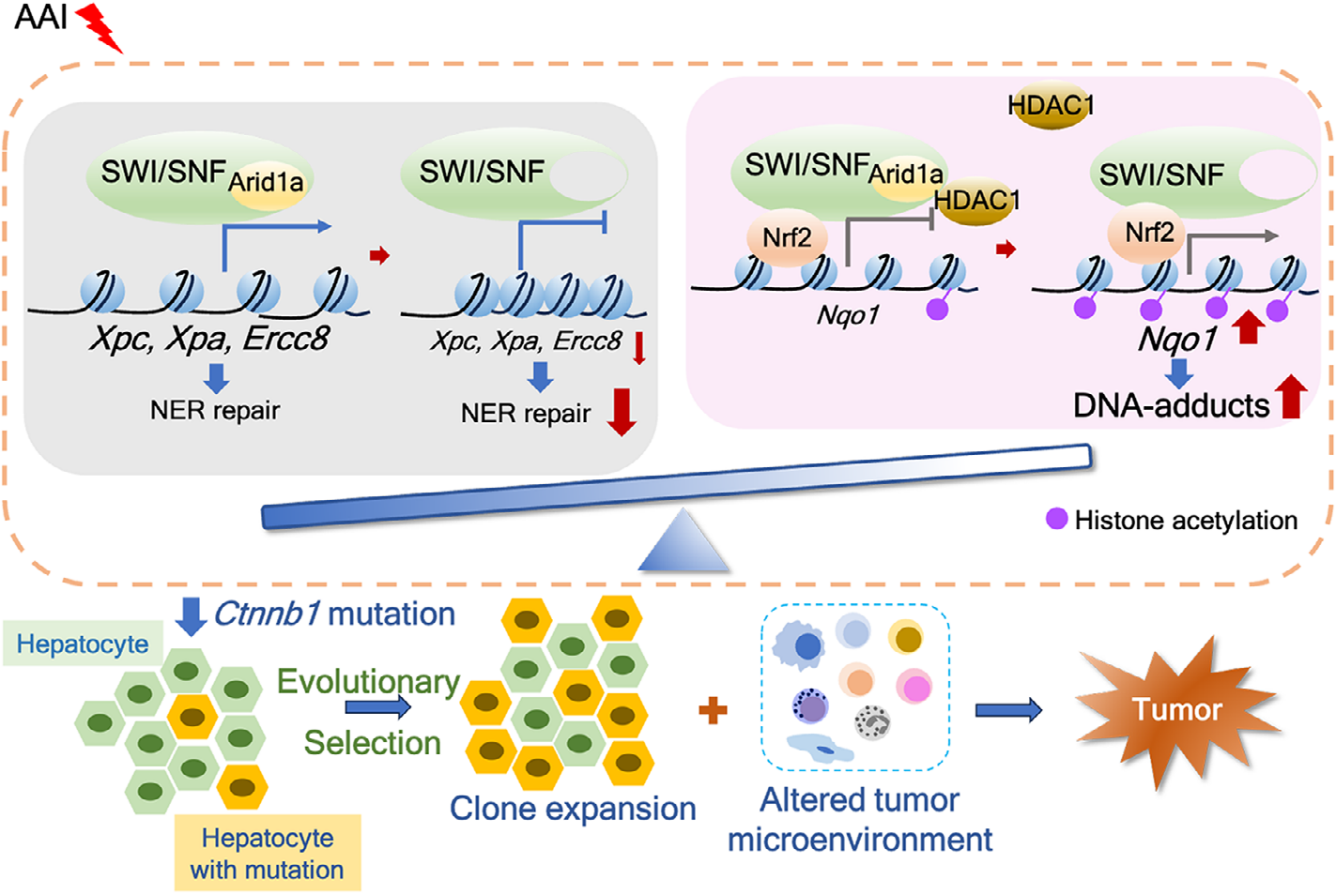
Schematic of working model of AAI‐triggered liver tumorigenesis in *Arid1a*‐deficient context.

Significantly, our study revealed that *Arid1a* deficiency reduces NER efficiency, increases AAI‐induced DNA adduct formation, alters the liver microenvironment, and ultimately results in elevated mutation burden in *Arid1a* knockout mouse livers under AAI treatment. Mechanistically, we demonstrated that *Arid1a* deficiency impairs transcription of key NER genes, such as *Xpa*, *Xpc*, and *Ercc8*, involved in removing AAI‐induced DNA adducts by reducing chromatin accessibility at their promoters. Moreover, *ARID1A* deficiency significantly upregulates *NQO1*, encoding the main metabolic activation enzyme of AA,^[^ ^45^ ^]^ likely due to reduced HDAC recruitment, increased histone H4 acetylation, and enhanced NRF2 and RNA polymerase II binding to the *NQO1* promoter. This dual effect—increased AAI‐induced DNA adducts and impaired DNA damage repair—creates a permissive environment for tumorigenesis (**Figure** **8**).

Interestingly, the accumulation of somatic genetic mutations provides a substrate for evolutionary selection during liver tumorigenesis. Specifically, in the absence of *Arid1a*, exposure to AAI induces a specific mutation in *Ctnnb1*, producing active β‐catenin lacking exon 3. Clonal architecture analysis revealed that the AAI‐induced *Ctnnb1* mutation conferred a growth advantage to *Arid1a*‐deficient cells under evolutionary pressure, supported by intrinsic genetic and epigenetic mechanisms and extrinsic microenvironmental changes. SnRNA‐seq showed that AAI treatment reshaped the immune microenvironment and cell‐cell communications in the livers of AAI‐treated *Arid1a^LKO^* mice, increasing inflammation and fibrosis and creating favorable conditions for the growth of *Arid1a*‐deficient cells with active β‐catenin signaling. One previous report revealed that the detoxification pathway regulator NR1L3 agonist induced *Tert*, *Myc*, and *Yap1* amplification, *Pten* deletion, and *Ctnnb1* mutation (S33A) in *Arid1a*‐deficient liver tumors. They identified *Ctnnb1* mutation (S33A) in one out of five tumors, which indicated that in their models, deficiency of *Arid1a* could cooperate with different oncogene activation, including *Ctnnb1* and tumor suppressor gene inactivation to promote tumor formation.^[^ ^13^ ^]^ In contrast, in our mouse models, AAI induces a high *Ctnnb1* mutation rate (9 out of 11) in liver tumor tissues from *Arid1a^LKO^* mice with AAI‐specific mutational signature, which resulted in exon 3 skipping of *Ctnnb1*. These results suggested that *Ctnnb1* mutations could be the primary synergistic oncogene, cooperating with *Arid1a* deficiency to promotes tumor formation. Moreover, our results indicated that the loss of *Arid1a* could not only result in weakened DNA damage repair but also upregulate in vivo metabolic activation enzyme such as NQO1, which led to a higher mutational burden induced by AAI in *Arid1a*‐deficient tissues. Under the process of cell clonal evolution in *Arid1a*‐deficient liver tissues, cells with *Ctnnb1* mutations may further expand and promote tumor formation.

Active β‐catenin signaling presents a candidate therapeutic target for these patients with ARID1A mutations. We demonstrated that the β‐catenin/CBP interaction antagonist ICG‐001 reduced the liver tumor incidence in AAI‐treated *Arid1a^LKO^* mice. Although we did not find the identical *CTNNB1* mutation site in HCC patients as the AAI‐induced *Ctnnb1* mutation in *Arid1a^LKO^* mouse livers due to species‐specific DNA sequence differences, *CTNNB1* mutations in exon 3 are prevalent in HCC patients. Patients with *ARID1A* mutation and SBS22a signature > 5% had the highest *CTNNB1* mutation rate and the worst prognosis. Thus, targeting the β‐catenin signaling may offer a potential prevention and therapeutic strategy for patients with *ARID1A* mutations, particularly those exposed to chemical carcinogen like AAs.

Our work revolves around the long‐standing paradox of how *ARID1A* mutations in non‐malignant tissues collaborate with environmental carcinogens to initiate cancer. By revealing that the *AIRD1A* loss simultaneously enhances genotoxic stress (via *NQO1*‐mediated bioactivation) and cripples DNA repair (via NER suppression), we establish a unified “two‐hit” model for mutagen‐driven tumorigenesis. The discovery of recurrent *Ctnnb1* slicing mutations and therapeutic efficacy of β‐catenin inhibition provides mechanistic insights and translational strategies for preventing malignant transformation in *ARID1A*‐deficient, carcinogen‐exposed tissues.

## 4 Experimental Section

### Animal Studies

Liver‐specific *Arid1a*‐knockout (*Arid1a^LKO^*) mice were generated by crossing *Arid1a^f/f^* mice (provided by Zhong Wang, Cardiovascular Research Center, Massachusetts General Hospital, Harvard Medical School)^[^ ^57^ ^]^ with Albumin‐Cre mice (C57BL/6 background; Jackson Laboratory).^[^ ^58^ ^]^ For AAI treatment, 14‐day‐old mice received intraperitoneal (i.p.) injections of 5 mg kg^−1^ AAI (dissolved in PBS, 0.5 mg mL^−1^) every other day for three doses. The β‐catenin/CBP interaction antagonist ICG‐001 (30 or 40 mg kg^−1^) in vehicle (2% dimethylsulfoxide, 50% PEG 300, 5% Tween 80) was administered i.p. every other day, three days/week, starting at the indicated times. Mice were euthanized at specific time points, and livers, spleens, and tails were collected.

For the xenografts, 5‐week‐old male BALB/c mice (SLAC Laboratory Animal, Shanghai, China) were maintained under pathogen‐free conditions. HuH‐7 cells (1.2 × 10^6^), either wild‐type or *ARID1A*‐knockout, infected with lentivirus expressing control or Flag‐tagged β‐catenin‐∆E3, were resuspended in 100 µL of a 1:1 mixture of Cultrex Basement Membrane Extract (R&D Systems, Catalog #3632‐005‐02) and PBS, then injected subcutaneously into the flanks. Tumor size was measured after the palpable tumors appeared, and tumor volume was calculated as Length × Width^2^/2. All mice were euthanized 41 days post‐inoculation, and tumors were collected. The maximum tumor size did not exceed 1500 mm^3^ at the end of the experiments.

Six‐ to eight‐week‐old *Arid1a^f/f^* or *Arid1a^LKO^* mice received hydrodynamic tail‐vein injections. A Sleeping Beauty transposon construct was generated by cloning Flag‐tagged human β‐catenin lacking exon 3 into pSBbi‐GN vector (Addgene #60517).^[^ ^59^ ^]^ A mixture containing 10 µg pSBbi‐GN‐Flag‐β‐catenin‐∆E3 and 0.5 µg sleeping beauty transposase plasmid pCMV(CAT)T7‐SB100 (Addgene #34879) was diluted in 2 mL sterile 0.9% NaCl, filtered through 0.22 µm membrane, and injected into the tail vein within 10 seconds. Mice were sacrificed at 12–15 months of age.

Only male mice were used in this study. All the animals were maintained under specific pathogen free (SPF) condition. All animal experimental protocols were reviewed and approved by the Institutional Animal Care and Use Committee (IACUC) of Shanghai Jiao Tong University (O_A2021121). All the experimental procedures were performed following the approved guidelines.

### Cell Culture

The HCC cell line HuH‐7 (RRID: CVCL_0336) and kidney cell line HEK293T (RRID: CVCL_0063) were obtained from the Institute of Biochemistry and Cell Biology, Chinese Academy of Sciences. Both cell lines underwent pre‐delivery authentication via STR profiling and microbial contamination screening (including mycoplasma, bacterial, fungal, and viral testing). Cells were expanded and cryopreserved at passage 3 for later experiments. MEFs were isolated from *Arid1a^f/f^* embryos at embryonic day 13.5 (E13.5) using an established protocol. *Arid1a* knockout was induced in low‐passage MEFs (P1‐P3) via Ad‐Cre. All cells were maintained in DMEM supplemented with 10% fetal bovine serum (FBS) and 100 µg mL^−1^ penicillin‐streptomycin at 37 °C in a humidified incubator with 5% CO_2_.

### Antibodies and Reagents

The antibodies used were as follows: anti‐Arid1a (Cell Signaling Technology, #12354; Abcam, ab182560), anti‐Ck19 (Abcam, ab52625), anti‐GS (Abcam, ab73593), anti‐Ki67 (Cell Signaling Technology, #12202), anti‐β‐Catenin (Cell Signaling Technology, #9582), anti‐Xpc (Abclonal, A8354), anti‐Xpa (Proteintech, 16462‐1‐AP), anti‐Ercc8 (Proteintech, 10818‐1‐AP), anti‐Brg1 (Abcam, ab110641), anti‐Nqo1 (Abmart, T56710F), anti‐Nrf2 (Cell Signaling Technology, #12721), anti‐HDAC1 (Abcam, ab280198), anti‐histone H4 (acetyl Lys8) (Abcam, ab15823), anti‐acetyl‐histone H4 (Millipore, 06–598), anti‐RNA polymerase II (Millipore, 05–623) and anti‐Gapdh (Proteintech, 60004‐1‐Ig).

The reagents used were as follows: AAI (Sigma, 5512), ICG‐001 (Selleck, S2662), ACBI1 (MCE, HY‐128359), AU‐15330 (Selleck, E1103), BRM014 (MCE, HY‐119374), and proteinase inhibitor (TargetMol, C0001).

### Recombinant Adenovirus and Lentivirus Preparation

Ad‐GFP or Ad‐Cre was prepared as previously described.^[^ ^58^ ^]^ The sgRNA targeting *ARID1A* (sg*ARID1A*) or NQO1 (sg*NQO1*) were cloned into pLentiCRISPRv2 (Addgene, #52961) vector. Flag‐tagged human β‐catenin cDNA lacking exon 3 of *CTNNB1* (Flag‐β‐catenin‐∆E3) was subcloned into lentivirus expressing vector. Lentiviral particles were produced by transfecting HEK293T cells with the lentiviral vector, envelope plasmid pMD2.G, and packaging plasmid psPAX2 using Lipofectamine 2000 (Invitrogen, 11668019). The sgRNA target sequences used are provided in Table S5 (Supporting Information).

### Vector Construction

Human XPA (NM_000380.4) was subcloned from pOTB7‐XPA vector and constructed into pcDNA3.1 vector with a Flag tag. Human XPC (NM_004628.5) was cloned and constructed into pcDNA3.1 vector with a Flag tag. Vectors were transfected into cells using Lipofectamine 2000 following the instruction of manufacturer.

### Construction of Stable Cell Lines

MEFs isolated from *Arid1a^f/f^* mice (MEF*^Arid1af/f^*) were infected with Ad‐GFP or Ad‐Cre overnight, followed by 48‐hour treatment with 1.25 µm AAI. Cells were then seeded at very low density in 96‐well plates to ensure single‐cell isolation. Colonies were expanded and passaged over ten times for further experiments.

For *ARID1A* knockout in HuH‐7 cells using CRISPR/Cas9 system, cells were infected and selected with 2 µg mL^−1^ puromycin for 48 hours, then plated at low density until colonies formed. *ARID1A* knockout efficiency was confirmed by Western blotting.

### Histologic Analysis

Histologic analysis was performed as previously described.^[^ ^58^ ^]^ Briefly, tissues were fixed in 4% paraformaldehyde and paraffin‐embedded. Tissue sections were deparaffinized, rehydrated, and stained with H&E. For immunohistochemistry, sections were heated in 10 mm citric acid buffer (pH 6.0) or 10 mm Tris‐EDTA (pH 9.0) with 0.5% Tween‐20 for 30 minutes for antigen retrieval. After blocking with horse serum, primary antibodies were incubated overnight at 4 °C in a humidified chamber, followed by secondary antibody incubation and immunoperoxidase labeling using Vectastain ABC kit (Vector Laboratories, PK‐4000), with hematoxylin counterstaining.

### RT‐Qpcr

Total RNAs were isolated from mouse tissues or cells using TRIzol reagent (ThermoFisher Scientific, 15596018CN), and cDNAs were reverse‐transcribed following the manufacturer's instructions. Real‐time PCR was performed using SYBR Green master mix (Takara, RR420) on LightCycler96 system (Roche). The primer sequences for RT‐PCR were listed in Table S5 (Supporting Information).

### Western Blotting Assay

Tissues were homogenized and cells were lysed in lysis buffer (50 mm Tris‐HCl, pH 7.4, 40 mm NaCl, 1 mm EDTA, 1% Triton X‐100, 1.5 mm Na_3_VO_4_, 50 mm NaF, 10 mm sodium pyrophosphate, 10 mm sodium β‐glycerophosphate) supplemented with protease inhibitor. Equal amounts of total protein were denatured, separated by SDS‐polyacrylamide gel electrophoresis, and transferred onto nitrocellulose membranes. Membranes were incubated with specific primary antibodies overnight at 4 °C, followed by incubation with corresponding secondary antibodies. Protein bands were visualized using ECL reagent on AI800 system (GE).

### ChIP Assay

ChIP was performed as described.^[^ ^60^ ^]^ Briefly, 9 × 10^6^ HuH‐7 cells were crosslinked with 1% formaldehyde for 10 min at room temperature, quenched with 125 mm glycine, and washed twice with 1 × PBS. Cell nuclei were extracted using lysis buffer 1 (50 mm HEPES, pH 7.5, 140 mm NaCl, 1 mm EDTA, 10% glycerol, 0.5% Nonidet P‐40, and 0.25% Triton X‐100) and lysis buffer 2 (10 mm Tris‐HCl, pH 8.0, 200 mm NaCl, and 1 mm EDTA). The cell nuclei were resuspended with lysis buffer 3 (10 mm Tris‐HCl, pH 8.0, 100 mm NaCl, 1 mm EDTA, and 0.1% sodium deoxycholate) and sonicated. After centrifugation, the lysates were incubated with protein A/G beads pre‐bound to IgG or specific antibodies at 4 °C overnight. Beads were washed seven times with RIPA buffer (50 mm HEPES, pH 7.6, 500 mm LiCl, 1 mm EDTA, 1% Nonidet P‐40, and 0.7% sodium deoxycholate) and once with TE buffer (10 mm Tris‐HCl, pH 8.0, 1 mm EDTA) containing 50 mm NaCl. The precipitated DNA was eluted with elution buffer (50 mm Tris‐HCl, pH 8.0, 10 mm EDTA, 1% SDS) at 65 °C for 20 min and reverse‐crosslinked at 65 °C overnight. The immunoprecipitated DNA was extracted with PCR purification kit (Qiagen, 28104) and analyzed by real‐time PCR with primers specific to target genes (Table S5, Supporting Information). The occupancy data were expressed as the ratio of the Ct value of immunoprecipitated DNA to that of the input samples (1%) or as fold change relative to IgG.

### Colony Formation Assay

Cells were seeded in 6‐well plates (MEFs: 3 × 10^4^ cells/well; HuH‐7, 4.5 × 10^3^ cells/well) and cultured for 7–10 days until colonies formed. Then colonies were rinsed with 1 × PBS, fixed with methanol for 20 minutes, and stained with 0.5% crystal violet in 20% methanol for 20 minutes.

### Spheroid Colony Formation Assay

MEFs were seeded in 24‐well ultralow attachment plates (Corning, 3473) at a density of 1 × 10^5^ cells/well with DMEM/F12 supplemented with 20 ng mL^−1^ basic fibroblast growth factor, 20 ng mL^−1^ epidermal growth factor, 1% B27, and 100 µg mL^−1^ penicillin‐streptomycin. Cells were maintained in a humidified incubator at 37 °C with 5% CO_2_. Apparent spheroid colonies appeared after 10–15 days.

### Detection of Ctnnb1 Mutation

Genomic DNA flanking the intron 2/exon 3 splice acceptor site in *Ctnnb1* was amplified from mouse tissues and subjected to Sanger sequencing.

RNA from mouse tissue samples was reverse‐transcribed to cDNA. PCR amplification across exons 2–4 of *Ctnnb1* was followed by Sanger sequencing. Primers are listed in Table S5 (Supporting Information).

### DNase I Hypersensitivity Analysis

Frozen liver tissue (30 mg) was thawed, minced on ice, and resuspended in lysis buffer (20 mm Tris‐HCL pH 7.5, 60 mm KCl, 5 mm spermidine, 0.15 mm spermine, 1 mm DTT, 50 mm EDTA, 1 mm EGTA). After incubation on ice for 10 minutes, the tissue was homogenized using Dounce homogenizer, filtered through 40 µm strainers, and centrifuged at 3000 rpm. Nuclei were washed twice with washing buffer (10 mm Tris‐HCL pH 7.4, 10 mm NaCl, 3 mm MgCl_2_), resuspended using DNase I digestion buffer, and treated with 0 U or 1 U of DNase I (NEB, M0303) at 37 °C for 15 minutes. Samples were digested with 0.2 mg mL^−1^ proteinase K at 55 °C overnight and 0.2 mg mL^−1^ RNase A at 37 °C for 1 hour. DNA was purified by phenol‐chloroform extraction. Primers for qPCR are listed in Table S5 (Supporting Information).

### Detection of AAI‐Derived DNA Adducts in Mouse Liver Tissues

Six‐week‐old male *Arid1a^f/f^* and *Arid1a^LKO^* mice received intraperitoneal injection of 5 mg kg^−1^ AAI for three consecutive days. Liver tissues were harvested 48 h post‐final injection. Wild‐type or *ARID1A* knockout HuH‐7 cells (1 × 10^7^) were treated with 12.5 µm AAI for 48 h. The liver tissues/cells were digested in lysis buffer (50 mm Tris‐HCl (pH 8.0), 100 mm EDTA (pH 8.0), 100 mm NaCl, 1% SDS) with proteinase K (0.2 mg mL^−1^) overnight at 55 °C, followed by incubation with RNase A (0.2 mg mL^−1^) at 37 °C for 2 hours.^[^ ^23^, ^61^ ^]^ DNA was extracted via phenol/chloroform purification. For adduct analysis, DNA (150 µg) per sample was diluted in 5 mm Tris‐HCl (pH 7.1) and 10 mm MgCl_2_, and sequentially digested with DNase I (Sangon Biotech, B002004), nuclease P1 (NEB, M0660S), alkaline phosphatase (Sangon Biotech, B006130), and phosphodiesterase I (Sangon Biotech, B003926). Proteins were precipitated with two volumes of chilled methanol. The supernatants were vacuum‐dried and reconstituted in H_2_O/Methanol (1:1, v/v).^[^ ^62^ ^]^ The reference standard of AAI‐derived DNA adduct 7‐(deoxyadenosin‐N6‐yl) aristolactam (dA‐AL‐I) was synthesized by incubating 4 mm deoxyadenosine (dA, Sangon Biotech, A620043), 0.15 µm AAI, and 20 mg Zn dust in 50 mm potassium phosphate (pH 5.8) at 37 °C overnight in the dark.^[^ ^63^ ^]^ Adducts were concentrated using Oasis PRiME HLB extraction cartridges (Waters, 186008057). dA‐AL‐I adducts were quantified by ultra‐performance liquid chromatography coupled with triple quadrupole mass spectrometry (UPLC‐TQ/MS; Waters Acquity UPLC H‐Class/Xevo TQ‐TS).^[^ ^61^ ^]^ A calibration curve was generated by plotting the abundance of the dA‐AL‐I at *m/z* 543.16‐427.1 against the relative concentration of the synthetic dA‐AL‐I standard. Linear regression analysis of the calibration curve was used to determine the relative concentration of dA‐AL‐I in each sample based on the abundance detected by UPLC‐TQ/MS.

### Statistical Analysis

Experiments included at least three replicates. Data presentation and sample size for each experiment were demonstrated in the figure legends and Supporting Information. *P* values were calculated as indicated in figure legends and Supporting Information, with *p* < 0.05 considered statistically significant. Statistical analysis was performed by Prism, SPSS, and R (v4.3.0).

Detailed methodologies for WES, WGS, bulk RNA‐seq, snRNA‐seq, ATAC‐seq, and data analysis are provided in the section of Supplementary Materials and Methods in Supporting Information.

## Conflict of Interest

The authors declare no conflict of interest.

## Author Contributions

L.W. and S.‐H.B. contributed equally to this work. Z.G.H. and L.W. conceived the project, designed the studies, and supervised the studies. L.W., S.‐H.B., S.‐J.S., X.‐L.Z., X.‐Y.S., Z.‐N.L., X.‐F.C., and X.‐L.Z. performed experiments and analyzed the data. S.‐H.B. analyzed the next‐generation sequencing data in this study. L.W. and S.‐H.B. interpreted the data and wrote the original draft. L.W. and Z.‐G.H. critically revised the manuscript; Z.‐G.H. and L.W. acquired the fundings supporting this study.

## Supporting information



Supporting Information

Supporting Information

Supporting Information

Supporting Information

Supporting Information

Supporting Information

## Data Availability

The data that support the findings of this study are available in the supplementary material of this article.
