# Supplementary material for: Arid1a Deficiency Drives Aristolochic Acid‐Induced Liver Tumorigenesis through Ctnnb1 Mutation and Defective Nucleotide Excision Repair
Source: Adv Sci (Weinh). 2025 Oct 24;13(3):e13981. doi: 10.1002/advs.202513981 (PMC12806303; doi:10.1002/advs.202513981)
Supplement: Supplementary file 1 — Supporting Information [file ADVS-13-e13981-s006.docx]

***Arid1a* Deficiency Drives Aristolochic Acid-Induced Liver Tumorigenesis through *Ctnnb1* Mutation and Defective Nucleotide Excision Repair**

Lan Wang^*^, Shi-Hao Bai, Shu-Jin Song, Xiao-Li Zhang, Xue-Ying Shang, Zhao-Ning Lu, Xiao-Fang Cui, Xin-Le Zhu, Ze-Guang Han^*^

L. Wang, S.-H. Bai, S.-J. Song, X.-L. Zhang, X.-Y. Shang, Z.-N. Lu, X.-F. Cui, X.-L. Zhu, Z.-G. Han

Key Laboratory of Systems Biomedicine (Ministry of Education) and State Key Laboratory of Medical Genomics, Shanghai Center for Systems Biomedicine, Shanghai Jiao Tong University, Shanghai 200240, China.

Lan Wang and Shi-Hao Bai contribute equally to this work.

*Correspondence:

Lan Wang, email: [lanwang@sjtu.edu.cn](mailto:lanwang@sjtu.edu.cn); Ze-Guang Han, email: [hanzg@sjtu.edu.cn](mailto:hanzg@sjtu.edu.cn).

**Table of contents**

1. Supplementary materials and methods………………………………………………………...4

1.1 Whole-exome sequencing (WES) and data analysis………………………………………4

1.2 Whole-genome sequencing (WGS) and data analysis……………………………………..5

1.3 Copy number variation (CNV) analysis…………………………………………………...6

1.4 Tumor clonal and subclonal analysis………………………………………………………6

1.5 Bulk RNA-seq and data analysis………………………………………………………..…7

1.6 Single-nucleus RNA-seq (snRNA-seq) and data analysis………………………………...8

1.7 The assay for transposase-accessible chromatin using sequencing (ATAC-seq)………..10

1.8 Alkaline comet assay…………………………………………………………………….11

1.9 Data availability………………………………………………………………………….12

1.10 Statistical analysis and visualization…………………………………………………....13

2. Supplementary Figures………………………………………………………………………14

Figure S1, Supporting Information…………………………………………………………..14

Figure S2, Supporting Information…………………………………………………………..15

Figure S3, Supporting Information…………………………………………………………..17

Figure S4, Supporting Information…………………………………………………………..19

Figure S5, Supporting Information…………………………………………………………..20

Figure S6, Supporting Information…………………………………………………………..21

Figure S7, Supporting Information…………………………………………………………..23

Figure S8, Supporting Information…………………………………………………………..24

Figure S9, Supporting Information…………………………………………………………..26

Figure S10, Supporting Information………………………………………………………. 28

Figure S11, Supporting Information………………………………………………………..30

Figure S12, Supporting Information………………………………………………………..32

Figure S13, Supporting Information………………………………………………………..34

Figure S14, Supporting Information………………………………………………………..36

Figure S15, Supporting Information………………………………………………………..38

Figure S16, Supporting Information………………………………………………………..40

Figure S17, Supporting Information………………………………………………………..42

Figure S18, Supporting Information………………………………………………………..43

Figure S19, Supporting Information………………………………………………………..44

Figure S20, Supporting Information………………………………………………………..45

Figure S21, Supporting Information………………………………………………………..47

Figure S22, Supporting Information………………………………………………………..48

Figure S23, Supporting Information………………………………………………………..49

Figure S24, Supporting Information………………………………………………………..50

Figure S25, Supporting Information………………………………………………………..51

Figure S26, Supporting Information………………………………………………………..52

Figure S27, Supporting Information………………………………………………………..53

Figure S28, Supporting Information………………………………………………………..54

3. Supplementary references…………………………………………………………………..56

**1. Supplementary Materials and Methods**

**1.1 WES and Data Analysis**

*Library Preparation and Sequencing*: DNA was extracted from liver tissues of tumors and adjacent non-tumorous tissues from 4-, 6-, 8-, 12-, 14-, and 15-month-old *Arid1a^f/f^* or *Arid1a^LKO^* mice treated with PBS, AAI, or DEN, using the phenol-chloroform method. DNA from spleens or tails of the corresponding mice served as controls to exclude germline mutations. DNA concentration was quantified by the Qubit 2.0 DNA Assay Kit (Invitrogen). Subsequently, DNA was randomly fragmented using a Covaris instrument. WES libraries were prepared and captured using the SureSelect Mouse All Exon V5/V6 kit (Agilent) according to the manufacturer’s instructions. The libraries were then sequenced on an Illumina HiSeq platform to generate 150 bp paired-end reads. WES was performed with an average depth of 295-fold (range: 242- to 468-fold) for liver tumors and adjacent non-tumorous tissues, and 79-fold (range: 62- to 109-fold) for spleen and tail samples.

*Somatic Mutation Detection and Annotation*: Following the removal of low-quality reads using Fastp (v0.23.1), WES reads were adapter-trimmed and aligned to the *Mus musculus* GRCm38 reference genome using BWA.^[1]^ SAMtools (1.16.1)^[2]^ and Picard (v2.26.11) were used to generate coordinate-sorted BAM files and remove PCR duplicate reads. Local realignment and base quality score recalibration of the BWA-aligned reads were performed using the Genome Analysis Toolkit (GATK, v4.2.0.0).^[3]^ Somatic variants in liver tumors and adjacent non-tumorous liver tissues were identified using MuTect2,^[4]^ with DNA from spleen or tail samples serving as matched normal controls. Variants recorded in the dbSNP database (dbSNP build 142)^[5]^ were excluded. To ensure high-quality somatic variants, the data were further filtered using the following criteria: a maximum sequencing depth of 500 ×; a minimum coverage of 30 × in tumors and adjacent non-tumorous tissues; a minimum coverage of 10 × in spleens or tails; at least 5 variant reads in tumors and adjacent non-tumorous tissues; no variant reads in spleens or tails.^[6]^ The filtered somatic mutations were functionally annotated using ANNOVAR^[7]^ with the RefGene database.

*Mutational Signature Analysis*: We analyzed three different variant classes—single base substitutions (SBS), doublet base substitutions (DBS) and small insertions and deletions (ID)—in 17 liver tumors, 9 adjacent non-tumorous liver tissues from *Arid1a^LKO^*, AAI-treated or DEN-treated *Arid1a^LKO^* mice using the R package MutationalPatterns (v3.10.0).^[8]^ For SBS, we generated 96 mutational contexts based on six substitution types (C > A, C > G, C > T, T > A, T >C, T > G) and their 16 possible flanking bases combinations. To infer *de novo* SBS signatures and determine the optimal number of factors, we applied non-negative matrix factorization (NMF) with 100 random resampling iterations, which identified two robust signatures. We calculated cosine similarities using MutationalPatterns to compare these *de novo* signatures with all 96 COSMIC signatures (mm10, version 3.4). Contribution scores were computed, retaining only COSMIC signatures with scores > 100 to avoid overfitting. COSMIC signature SBS22a emerged as the dominant, showing the highest cosine similarity in AAI-induced tumors and some adjacent non-tumorous tissues. To assign specific SNVs to signatures, we used Bayesian inference via the R package Palimpsest (v2.0.0),^[9]^ enabling assessment of signature contributions to individual genes. For DBS and ID signatures, we applied the same NMF-based *de novo* analysis approach as for SBS. Cosine similarities and contribution scores were calculated by comparing these signatures to published COSMIC signatures (mm10, v3.4) using MutationalPatterns.

**1.2 WGS and Data Analysis**

DNA was extracted from liver tissues (tumors and adjacent non-tumorous tissues) of 4-, 6-, 8-, 12-, 14-, and 15- month-old *Arid1a^f/f^* or *Arid1a^LKO^* mice treated with PBS or AAI. DNA from spleen or tail samples of each mouse served as matched germline controls to exclude germline CNVs. DNA concentration was quantified using Qubit 2.0 fluorometer, and sequencing libraries were prepared using the TruSeq DNA Sample Prep Kit (Illumina). Sequencing was performed on the Illumina HiSeq platform to generate 150 bp paired-end reads. For CNV detection, low-coverage WGS was performed with an approximate depth of 4 × for all samples.

**1.3 CNV Analysis**

Following quality control with Fastp (v0.23.1), processed reads were aligned to the *Mus musculus* GRCm38 reference genome using BWA (v0.7.17),^[1]^ consistent with the WES workflow. BAM files were sorted and indexed using SAMtools (v1.16.1). CNV analysis was performed using Control-FREEC (v11.6)^[10]^ with a bin size of 10,000 bp. The algorithm traversed chromosomes using a sliding window of 50,000 bp, advancing in 10,000 bp steps. Genomic regions with copy numbers significantly deviating from the diploid baseline (2 copies) in autosomes were identified as potential CNVs. DNA from spleen or tail samples served as matched germline controls to specifically detect somatic CNVs in tumors and adjacent non-tumorous tissues using identical parameters. For validation, CNVs in paired samples were independently verified using CNVkit (v0.9.7).^[11]^ Finally, a chromosome-scale heatmap visualizing genomic alterations was generated using CNVkit and the R statistical environment.

**1.4 Tumor Clonal and Subclonal Analysis**

To identify subclonal populations within individual tumors and adjacent non-tumorous tissues, we employed the R package SciClone (v1.1.1)^[12]^ using filtered somatic mutations and CNVs. Variant allele frequency (VAF) served as the primary metric for inferring subclonal clusters, while CNV information refined the model. A minimum sequencing depth of 70-fold at variant sites was required to ensure reliable VAF estimation.

Possible evolutionary models were generated and visualized using the R package ClonEvol (v0.99.11),^[13]^ which constructed fishplots based on VAF clusters identified by SciClone (Figure S10C, Supporting Information). Mutational types and signatures were visualized using ggplot2 and Adobe Illustrator (Figure S8, Supporting Information).

For tumor evolution and metastasis analysis, we analyzed WES data from liver tissues of 6- and 8-month-old AAI-treated *Arid1a^LKO^* mice harboring multiple sequenced tumors. To avoid potential loss of subclonal variants during filtering, we retained and examined all somatic mutation sites across all tumors and adjacent non-tumorous tissues. VAF was calculated as the proportion of mutation-supporting reads to total reads at each genomic position.

**1.5 Bulk RNA-seq and Data Analysis**

Total RNA was isolated from seven frozen liver tumor tissues and two adjacent non-tumorous tissues of 8-month-old *Arid1a^LKO^* mice using TRIzol reagent (Invitrogen). RNA integrity was assessed using an Agilent 2100 Bioanalyzer (Agilent Technologies), and only samples meeting quality thresholds (RNA integrity number [RIN] > 7, no detectable contamination, and sufficient in quantity) were used for library preparation. Transcriptome libraries were constructed using the NEBNext® Ultra RNA Library Prep Kit for Illumina (NEB) according to the manufacturer’s instructions. Libraries were sequenced on an Illumina HiSeq platform, generating 150 bp paired-end reads, yielding an average of 46 million reads per sample for tumor and adjacent non-tumorous tissues.

After removing rRNA reads, adaptor sequences, and low quality reads, filtered reads were aligned to *Mus musculus* genome (GRCm38 assembly with UCSC annotated transcripts) using Bowtie2 (v2.4.4) ^[14]^. Transcript quantification was performed against the Ensembl annotated reference genome (*Mus musculus*, GRCm38, release 94) using FeatureCounts (v2.0.3).^[15]^ Differential gene expression analysis was conducted with DESeq2 (v1.40.2),^[16]^ applying thresholds of fold change > 1.5 and *p* value < 0.05. Gene Ontology (GO)^[17]^ and Kyoto Encyclopedia of Genes and Genomes (KEGG)^[18]^ pathway analyses were performed using ClusterProfiler (v4.8.3).^[19]^ Gene set enrichment analysis (GSEA)^[20]^ was performed using gene set variation analysis (GSVA, v1.48.3)^[21]^ using ‘Hallmark gene sets’ from the Molecular Signature Database (MsigDB) (<http://software.broadinstitute.org/gsea/msigdb)>.

**1.6 snRNA-seq and data analysis**

*Nuclei Isolation from Frozen Tissues*: Approximately 50 mg of fresh-frozen liver tissue was thawed on ice and minced into small fragments using scalpels. Tissue fragments were homogenized in low-sucrose buffer (0.32 M sucrose, 10 mM Hepes pH 7.4, 5 mM CaCl_2_, 3 mM MgCl_2_, 0.1 mM EDTA, 1 mM DTT and 0.1 U/μl RNase inhibitor in nuclease-free water) using a Dounce homogenizer. The homogenate was filtered through a 40 μm strainer, and the crude nuclei were pelleted by centrifugation at 500 × g for 5 min at 4 °C, followed by two washes in low sucrose buffer. The crude cell nuclei were purified through a sucrose density cushion (1.2 M sucrose, 10 mM HEPES pH 7.4, 3 mM MgCl_2_ and 0.1 U/μl RNase inhibitor) by centrifugation at 3200 × g for 10 min at 4 °C. The pellet was resuspended in PBS with 1% BSA, stained with 7-AAD (1:100 dilution) for 10 minutes, and subjected to flow cytometry for debris removal. Purified nuclei was counted, and approximately 10,000 nuclei per sample were used for library preparation.

Libraries were prepared using the 10 × Genomic Chromium Single Cell 3’ v3 kit according to the manufacturer’s instruction and sequenced on a Novo-seq platform, generating 150 bp paired-end reads with an average depth > 34,000 reads per nucleus.

*SnRNA-seq Data Analysis*: After quality control, reads were aligned to the *Mus musculus* reference genome (GRCm38) using Cell Ranger (v6.0.1). Gene expression was quantified by counting reads spanning exons and introns to capture pre-mRNA and mature mRNA. Following initial Cell Ranger filtering, expression matrices were processed using Seurat (v4.3.0)^[22]^ with additional quality filters: (a) genes detected in ≥ 3 nuclei; (b) Nuclei expressing > 500 genes; (c) nuclei with < 10% mitochondrial unique molecular identifiers (UMIs). Quality metrics were assessed using scater (v1.28.0), and doublets were removed using DoubletFinder (v2.0.3).^[23]^ Datasets were integrated using Seurat’s IntegrateData function to correct batch effects. Differentially expressed genes between clusters and tissues were identified using Seurat’s FindMarkers/FindAllMarkers. Dimensionally reduction was performed via Principal component analysis (PCA) and nonlinear dimensionality reduction (UMAP).

*Unsupervised Clustering and Cell-Type Identification*: Clustering was performed at moderate resolution in Seurat. Cell identities were annotated using SingleR (v2.2.0)^[24]^ and scCATCH (v3.2.2)^[25]^ with manual verification against geometric means of canonical cell markers (Table S3, Supporting Information). Cell clusters co-expressing multiple lineage markers (indicative of residual doublets) were excluded. Remaining cells were re-clustered using optimized principle components. Hepatocyte subpopulations were mapped to spatial zonation signature using Seurat’s reference-based integration (FindTransferAnchors/TransferData) with dataset (GSE148339).^[26]^ Subcluster markers underwent GO/KEGG enrichment analysis.

*Cell-Cell Interaction Analysis*: CellphoneDB (v2.1.7)^[27]^ analyzed ligand-receptor interactions in 8,000 randomly sampled cells from normal, adjacent non-tumorous, and tumor tissues. Interaction strengths were quantified using build-in databases. Pathway-level communication networks were reconstructed using CellChat (v1.1.3)^[28]^ with curated signaling gene sets.

*Copy Number Inference from SnRNA-seq*: InferCNV (v1.16.0)^[29]^ inferred CNVs in 2,000 hepatocytes from four tissue samples (SH30N, SH30T1, SH12N and SH12T4). Normal liver profiles served as reference controls, with noise reduction via Hidden Markov Model smoothing. Lineage relationships were visualized using treeio (v1.24.3) and ggtree (v3.8.2).

*Pseudotime Analysis using Monocle*: Monocle2 (v2.28.0)^[30]^ constructed pseudotime trajectories for 6,000 randomly selected hepatocytes from adjacent non-tumorous and tumor tissues. Cells were ordered by differentially expressed genes along the trajectory and annotated by tissue origins.

**1.7 ATAC-seq**

Cell nuclei were isolated from frozen mouse liver tissues of 8-month-old AAI-treated *Arid1a^f/f^* (n = 6) and *Arid1a^LKO^* (n = 6) mice using the methods described in the DNase I hypersensitivity analysis in the section of Methods. The isolated cell nuclei were subjected to ATAC-seq analysis according to the previous report.^[31]^ Libraries were sequenced with a 150 bp paired-end sequencing strategy on an Illumina HiSeq X Ten platform. For ATAC-seq data analysis, we used Fastp (0.23.1) to remove adaptors. Then all the sequence reads for each sample were aligned to the *Mus musculus* GRCm38 reference genome using BWA (0.7.17). Duplicates were marked by Picard (v2.26.11). SAMtools (v1.16.1) was used to filter low-quality reads and mitochondrial DNA reads. Narrow peaks were called using MACS2 (v.2.2.7). The significantly different peaks between AAI-treated *Arid1a^f/f^* and *Arid1a^LKO^* mice were analyzed by DiffBind (v3.10.1) with *p* value < 0.05. The differential peaks were annotated by ChIPseeker R package (v1.36.1) with gene symbols and regions. GO pathway enrichment analysis were performed using ClusterProfiler (v.4.8.3). Packaged commands (bamCoverage, computeMatrix, plotProfile and plotHeatmap) from deepTools (v3.5.3) were used to generate bigwig files and heatmap of ATAC-seq results.

**1.8 Alkaline Comet Assay**

Control and *ARID1A*-knockout HuH-7 cells were treated with AFB1 (25 μM) or cisplatin (10 μM) for 24 hours and subjected to alkaline comet assay. For AAI treatment of control and *ARID1A*-knockout HuH-7 cells, cells were infected with lentivirus expressing *NQO1*-specific sgRNA (sg*NQO1*) for 24 hours, screened with puromycin (2 μM) for 48 hours, and then treated with AAI (12.5 μM) for 48 hours. For XPC or XPA overexpression assay, cells were transfected with control vectors, XPC or XPA expressing vectors. Twenty four hours after transfection, cells were treated with AAI (12.5 μM) for 48 hours and then subjected to alkaline comet assay. After placing the base agarose layer onto the slides, the cultured cells were digested into single cell using trypsin, mixed with 0.7% low-melt point agarose and plated on top of the basal agarose layer. Embedded cells were treated with lysis buffer (2.5 M NaCl, 100 mM EDTA, 10 mM Tris, 200 mM NaOH, 10% DMSO, 1% Triton X-100) for 1 h at 4 °C. After rinsed with PBS, the embedded cells on slides were immerged into pre-chilled alkaline solution (300 mM NaOH, 1mM EDTA, 1% DMSO, pH > 13) for 40 min. The slides were then horizontally electrophoresed for 30 min in the alkaline solution at 0.75 V/cm. The slides were neutralized by neutralization buffer (400 mM Tris, pH 7.5), rinsed with 70% ethanol twice, dried at 37 °C, stained by SYBR Green I and then visualized using fluorescent microscope. Quantification was performed using the OpenComet plugin with ImageJ. Data were represented as tail moment combining tail length and %DNA in the tail.

**1.9 Data availability**

Public WES and bulk RNA-seq datasets from human HCC samples were obtained from multiple sources: The Cancer Genome Atlas Program (TCGA), International Cancer Genome Consortium (ICGC) projects (LICA-CN, LICA-FR, LINC-JP, and LIRI-JP), cBioPortal (http://www.cbioportal.org), NODE database (accession number OEP000321; <https://www.biosino.org/node/>) and Supplementary Table S3 from the study published under PMID: 31887370.^[32-40]^. Additionally, snRNA-seq data from wild-type mouse liver tissues were downloaded from Gene Expression Omnibus (GEO) (accessions GSE136103 and GSE148339) and integrated with our snRNA-seq from adjacent non-tumorous and tumor tissues of AAI-treated *Arid1a*-deficient mice.^[26, 41]^ ATAC-seq data from wild-type and *Arid1a*-knockout mouse hepatocytes were downloaded from GEO database (GSE111499).^[42]^ Public sourced data: DNase-seq (2-month-old mouse livers; ENCODE: ENCFF100XUL, ENCFF178LXB and ENCFF445QMM), NRF2 and RNA polymerase II (POL II) ChIP-seq (HepG2; ENCODE: ENCFF242DUB, ENCFF425QWO). To ensure analytical consistency, all public data were processed using identical pipeline to our in-house next-generation sequencing data.

The raw sequencing data generated in this study were deposited in SRA: WES (PRJNA1101668), WGS (PRJNA1101665), bulk RNA-seq (PRJNA1101671), snRNA-seq (PRJNA1101675) and ATAC-seq (PRJNA1331690).

**1.10 Statistical Analysis and Data Visualization**

Experiments included at least three replicates. Statistical analyses were performed using SPSS and R (v4.3.0) with *p* values calculated as specified in figure legends. A *p*-value < 0.05 was considered statistically significant. Data visualization was implemented with ggplot2 (v3.5.1), pheatmap (v1.0.12) and GraphPad Prism.

Mutually exclusive or co-occurring genetic alterations were identified and tested using somaticInteractions function in maftools^[43]^ (Figure 6B), generating corresponding *p* values. Read density maps were created from bigwig files using the Integrative Genomic Viewer (IGV). Survival analyses were performed with the survival and survminer R packages, with significance determined by Log rank tests.

**2. Supplementary Figures**

**
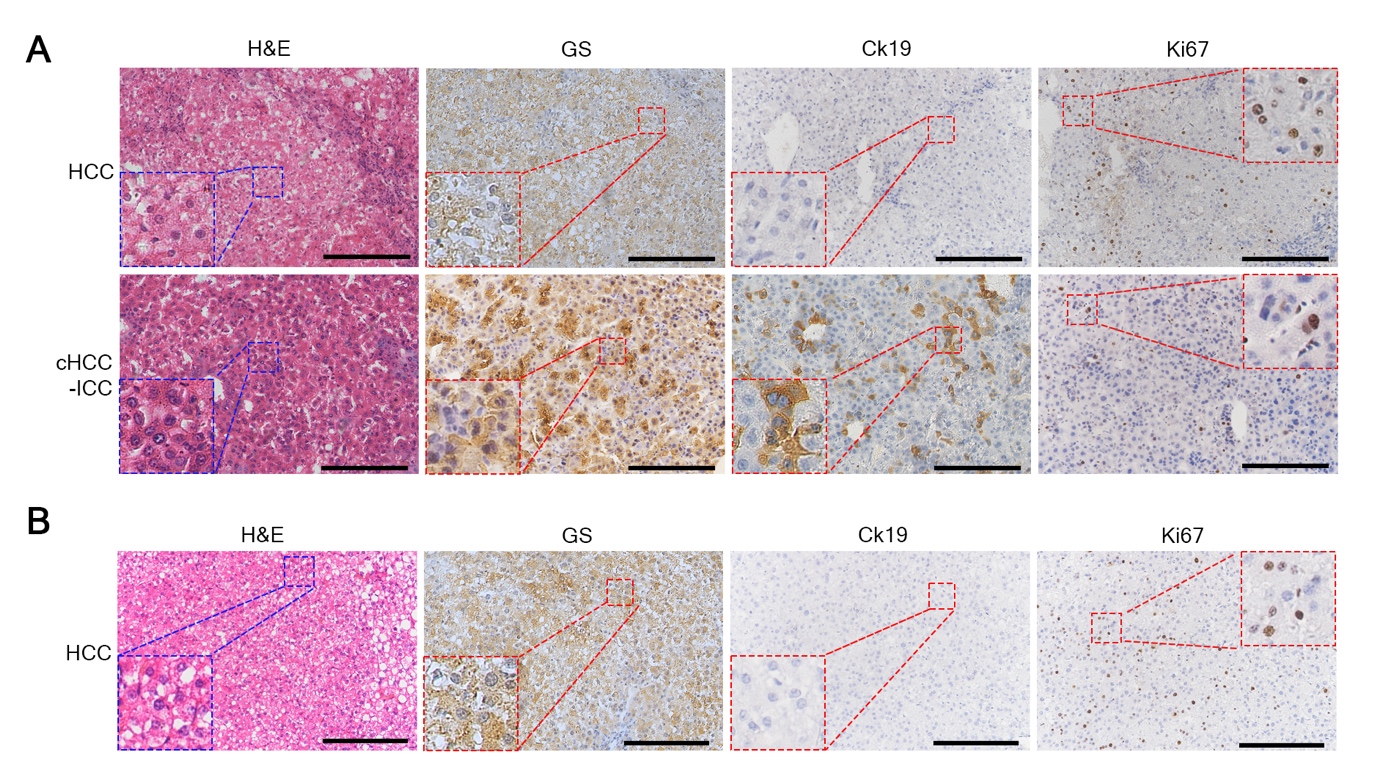
Figure S1, Supporting Information**

**Figure S1.** Representative hematoxylin and eosin staining and immunohistochemistry staining of liver sections. A-B) Staining was performed using the indicated antibodies on liver tissue sections from *Arid1a^LKO^* mice (A) and DEN-treated *Arid1a^LKO^* mice (B). Scale bar, 200 μm.

**Figure S2, Supporting Information**

**
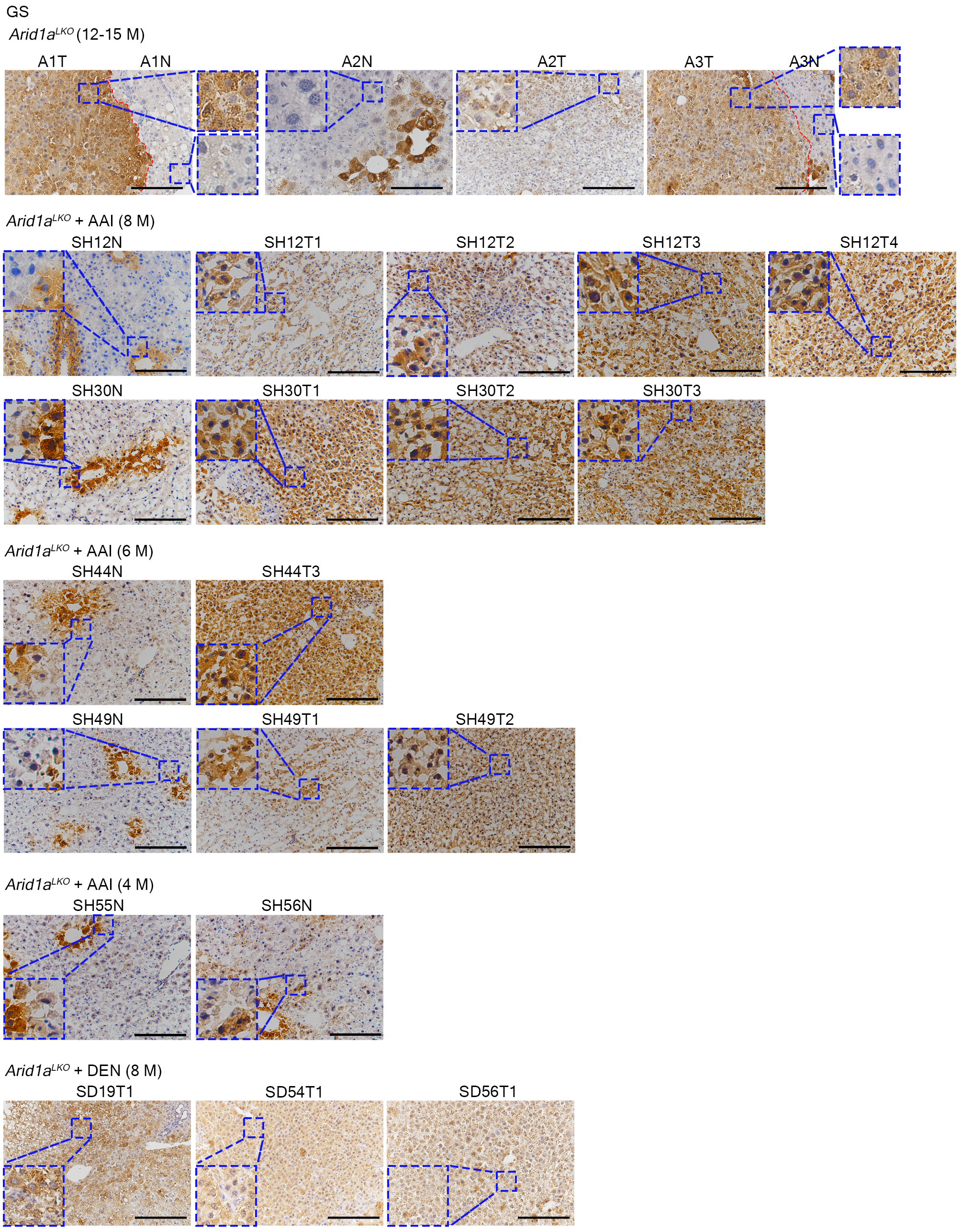
**

**Figure S2.** Immunohistochemistry of glutamine synthetase (GS) in mouse liver tissues used for next-generation sequencing. GS staining in liver sections from PBS-, AAI-, and DEN-treated *Arid1a^LKO^* mice. Scale bar, 200 μm.

**
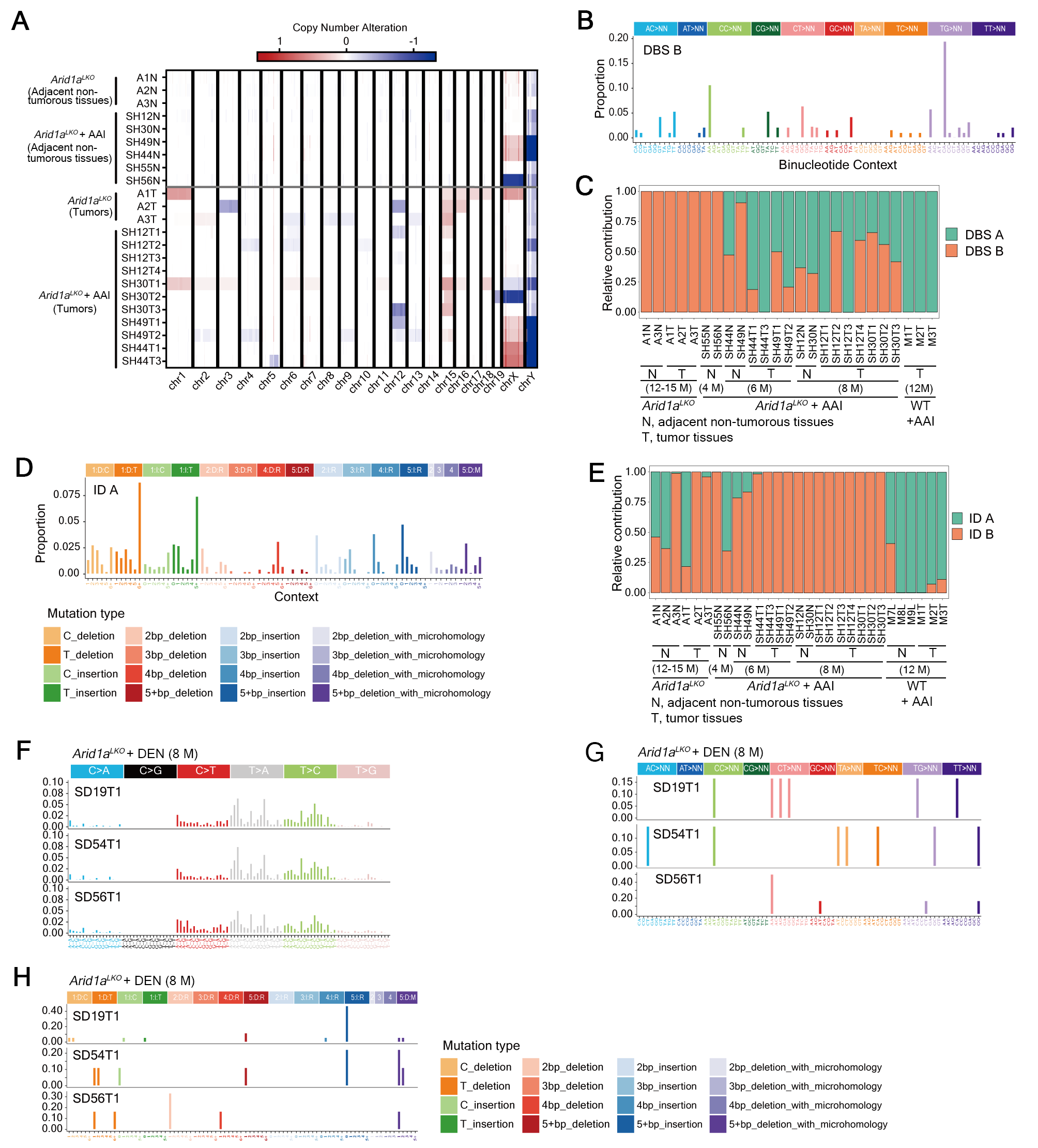
Figure S3, Supporting Information**

**Figure S3.** Genomic DNA CNVs and mutational signatures in AAI- and DEN-treated *Arid1a^LKO^* mice. A) Visualization of genomic DNA CNVs in liver tissues from *Arid1a^LKO^* and AAI-treated *Arid1a^LKO^* mice, analyzed using CNVkit software based on WGS data. B) *De novo* DBS B signature extraction from somatic mutations of *Arid1a^LKO^*, AAI-treated wild-type (WT) and AAI-treated *Arid1a^LKO^* mouse liver tissues. C) Proportion of *de novo* DBS A and B signatures in adjacent non-tumorous liver tissues (N) and tumors (T) from *Arid1a^LKO^*, AAI-treated WT and AAI-treated *Arid1a^LKO^* mouse. D) *De novo* ID A signature extraction from somatic mutations of *Arid1a^LKO^*, AAI-treated WT and AAI-treated *Arid1a^LKO^* liver tissues. E) Proportions of *de novo* ID A and B signatures in adjacent non-tumorous liver tissues (N) and tumors (T) from *Arid1a^LKO^*, AAI-treated WT and AAI-treated *Arid1a^LKO^* liver tissues. F-H) Analysis of SBS (F), DBS (G), and ID (H) mutational signatures in DEN-induced *Arid1a^LKO^* mice at 8 months of age.

**
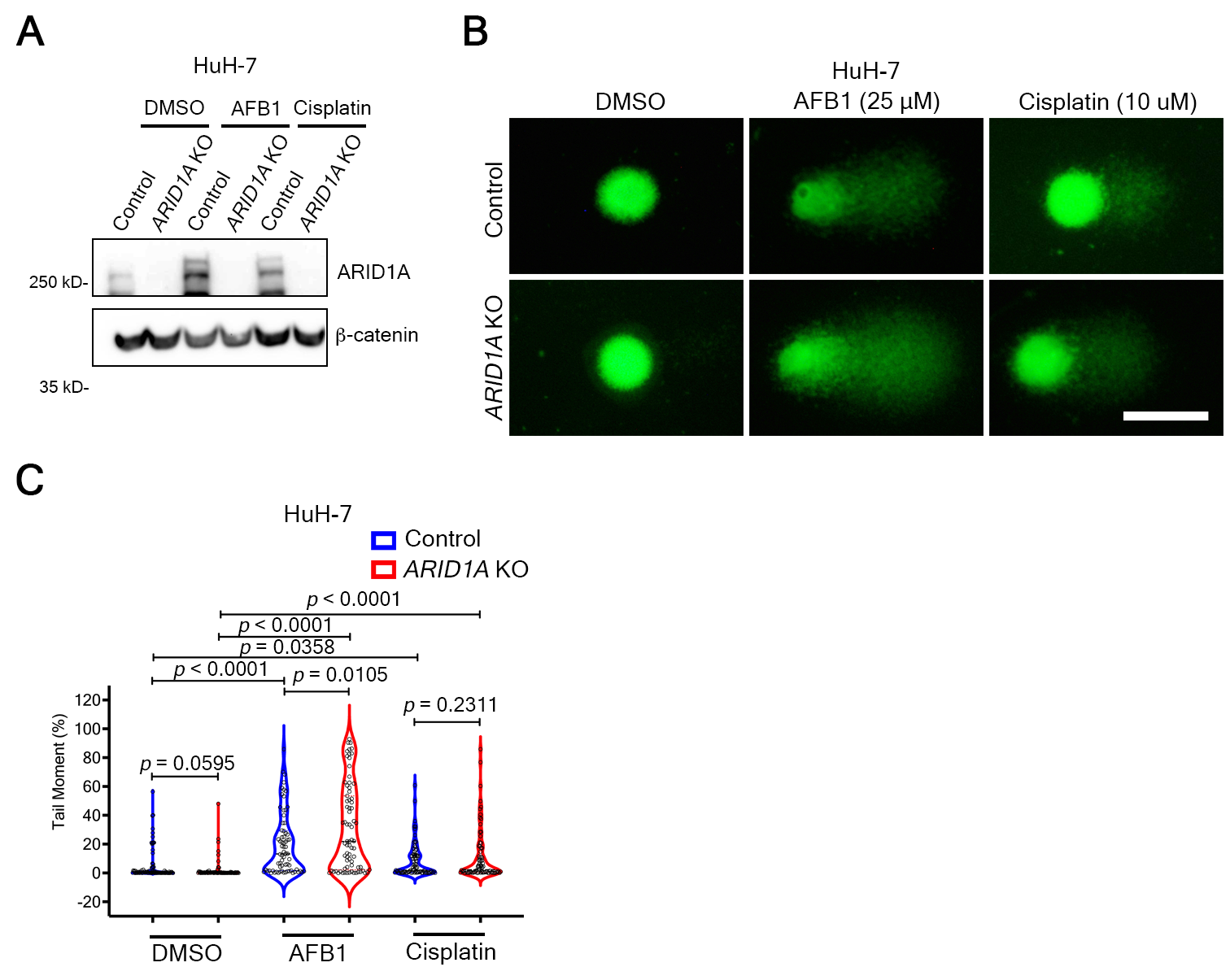
Figure S4, Supporting Information**

**Figure S4.** AFB1 and cisplatin induce DNA-damage in HuH-7. A) Western blotting of Control or *ARID1A*-knockout HuH-7 cells treated with DMSO, AFB1 (25 μM) or cisplatin (10 μM) for 24 h using indicated antibodies. B) Control or *ARID1A*-knockout HuH-7 cells in (A) were subjected to comet assays. Representative comet assays of HuH-7 cells. Original magnification, ×20. Scale bar, 50 μm. C) Violin plot of the quantification of the tail moment using OpenComet plugin of ImageJ. At least 50 cells was calculated for each group. Statistical significance was determined using two-sided unpaired Student *t*-test.

**Figure S5, Supporting Information**

**Figure S5.** Validation of AAI-induced *Ctnnb1* mutation in mouse liver tissues. A) PCR amplification of genomic DNA from spleens and liver tissues of AAI-treated *Arid1a^LKO^* mice using the primers from Figure 2I. B) Sanger sequencing results of PCR products from DNA extracted from spleens (SH12S, SH49S and SH44S), non-tumorous (SH12N) and tumor tissues (SH12T2, SH12T3, SH12T4, SH49T1, SH49T2 and SH44T3) of AAI-treated *Arid1a^LKO^* mice using the primers from Figure 2I. Red rectangles indicated the *Ctnnb1* mutation site induced by AAI. C) Representative alignment results of DNA sequences derived from the shorter PCR products shown in Figure 2K. **
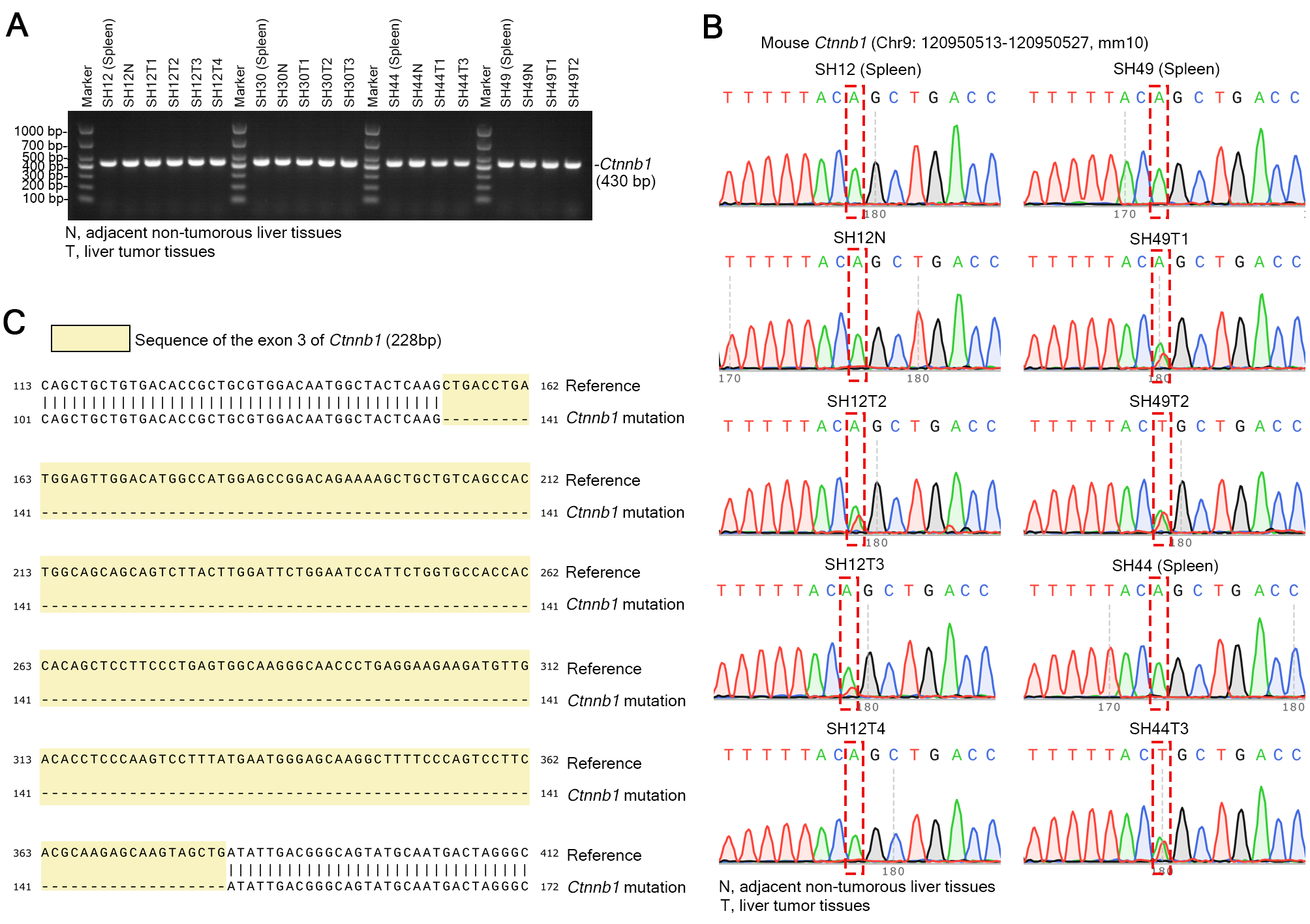
**

**
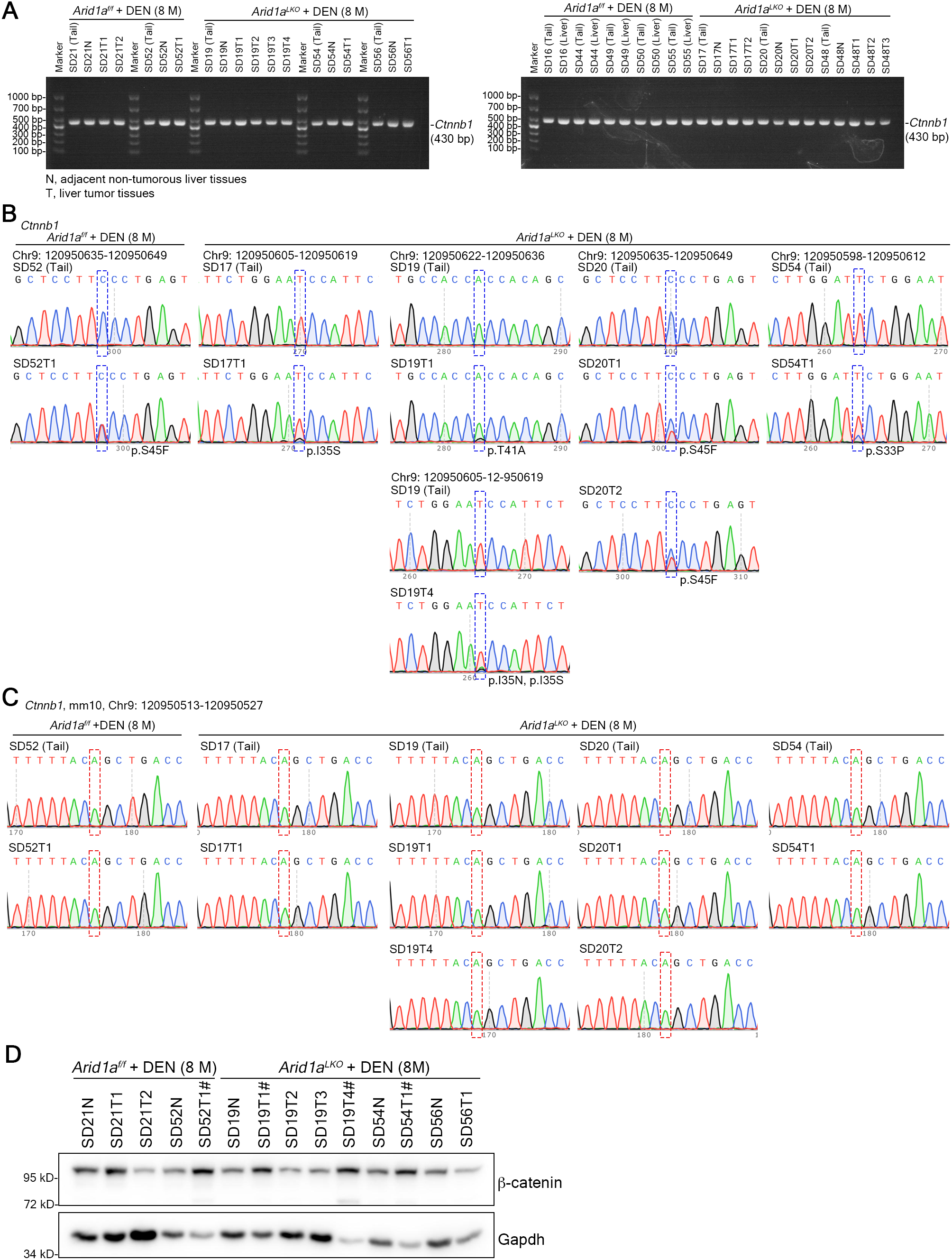
Figure S6, Supporting Information**

**Figure S6.** Validation of DEN-induced *Ctnnb1* mutations in mouse liver tissues. A) PCR amplification of genomic DNA from tails and liver tissues of DEN-treated *Arid1a^f/f^* or *Arid1a^LKO^* mice using the primers indicated in Figure 2I. Sanger sequencing was performed using all the amplified PCR products. B) Representative Sanger sequencing results of PCR products from (A). Blue rectangles indicate DEN-induced *Ctnnb1* mutations in tumors, while the same sites from mouse tails were used as references. C) Representative Sanger sequencing results of PCR products from (A). Red rectangles indicated the *Ctnnb1* mutation site specifically induced by AAI in *Arid1a^LKO^* mouse livers, which was absent in DEN-treated *Arid1a^f/f^* or *Arid1a^LKO^* mouse livers. D) Western blotting of adjacent non-tumorous tissues and tumors from DEN-treated *Arid1a^f/f^* and *Arid1a^LKO^* mice. Gapdh: loading control. #: samples with *Ctnnb1* mutations identified by Sanger sequencing in (B).

**
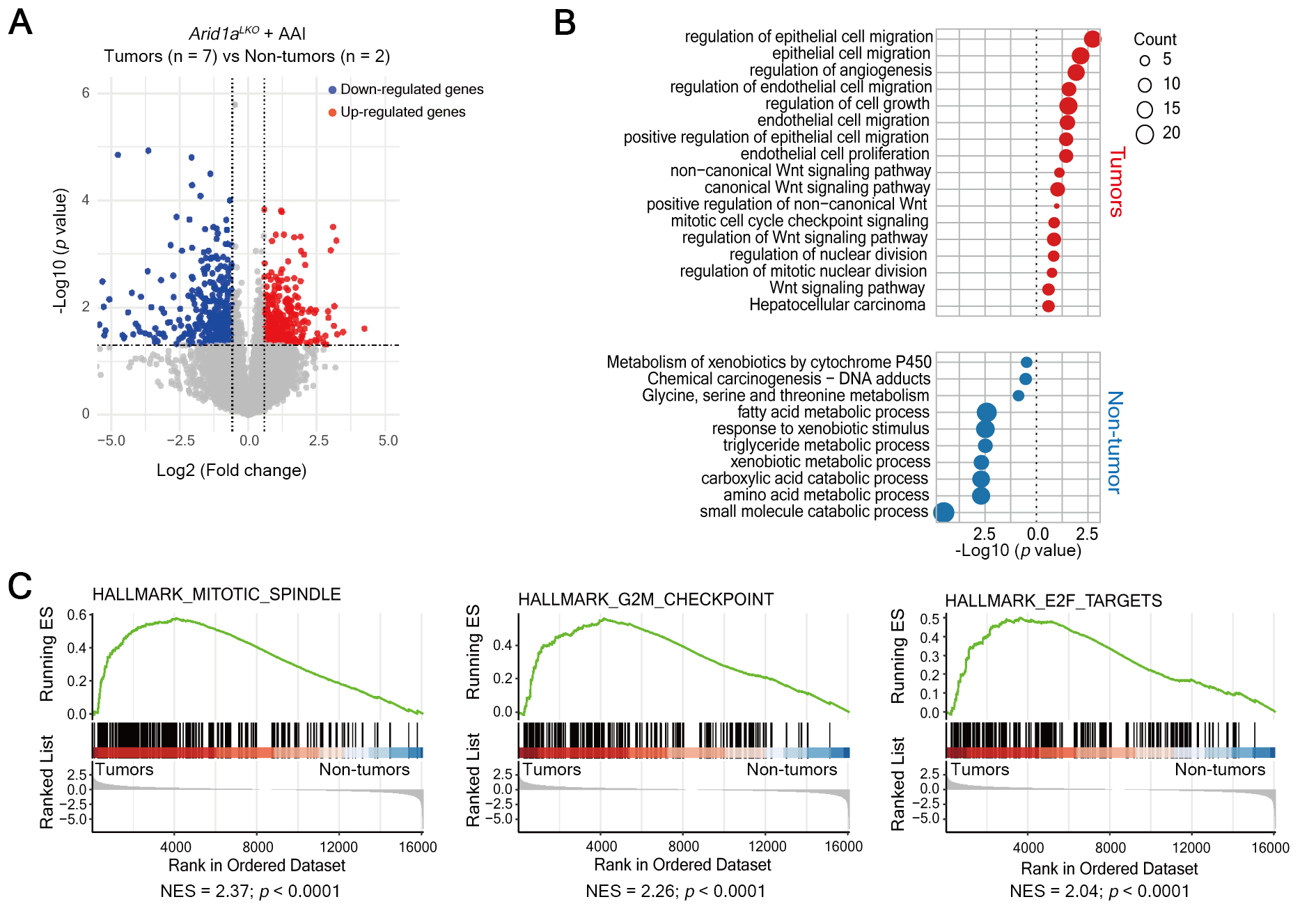
Figure S7, Supporting Information**

**Figure S7.** Analysis of differentially expressed genes between tumors and adjacent non-tumorous liver tissues. A) Volcano plot of differentially expressed genes between 7 tumor samples and 2 adjacent non-tumorous liver tissues from two AAI-stimulated *Arid1a^LKO^* mice (cutoff: fold change > 1.5 and *p* < 0.05). Blue or red dots represent the differentially expressed genes that were downregulated or upregulated in tumor tissues from AAI-stimulated *Arid1a^LKO^* mice based on bulk RNA-seq data. B) GO and KEGG analysis of enriched pathways in tumors (top) and adjacent non-tumorous tissues (bottom) based on bulk RNA-seq data in (A). C) GSEA analysis using Hallmark gene sets based on bulk RNA-seq data from (A).

**Figure S8, Supporting Information**

**
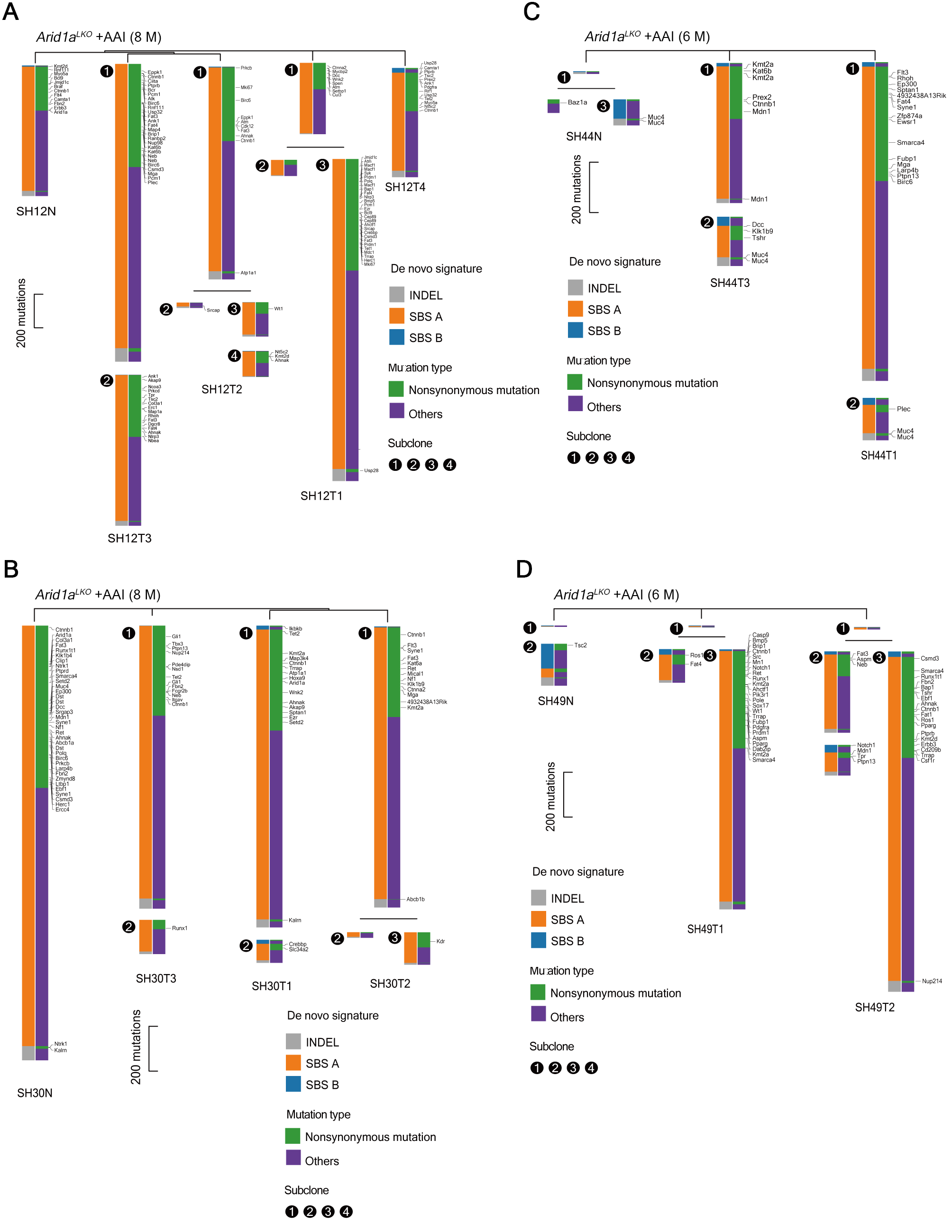
**

**Figure S8.** Clonal phylogenetic analysis of adjacent non-tumorous liver tissues and tumors. A-D) Subclone characterization by mutational signatures (left) and mutation types (right) in four AAI-treated *Arid1a^LKO^* mice. Driver mutations were labeled.

**Figure S9, Supporting Information**

**
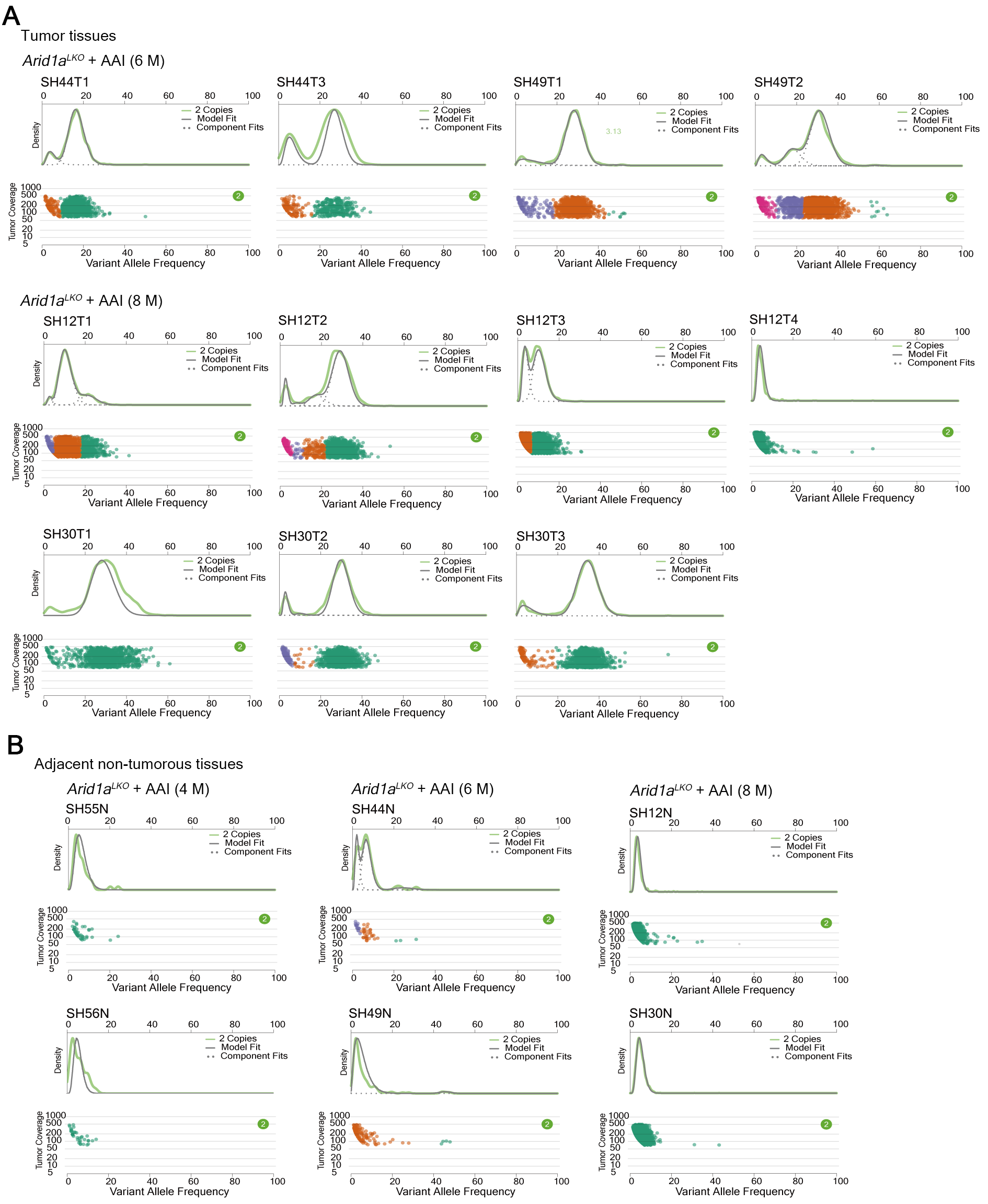
**

**Figure S9.** Clone composition analysis within tumor and adjacent non-tumorous tissues. A-B) Clone composition based on mutations was analyzed using SciClone in liver tumor tissues (A) and adjacent non-tumorous tissues (B) from AAI-treated *Arid1a^LKO^* mice sacrificed at 4- to 8-month-old.

**
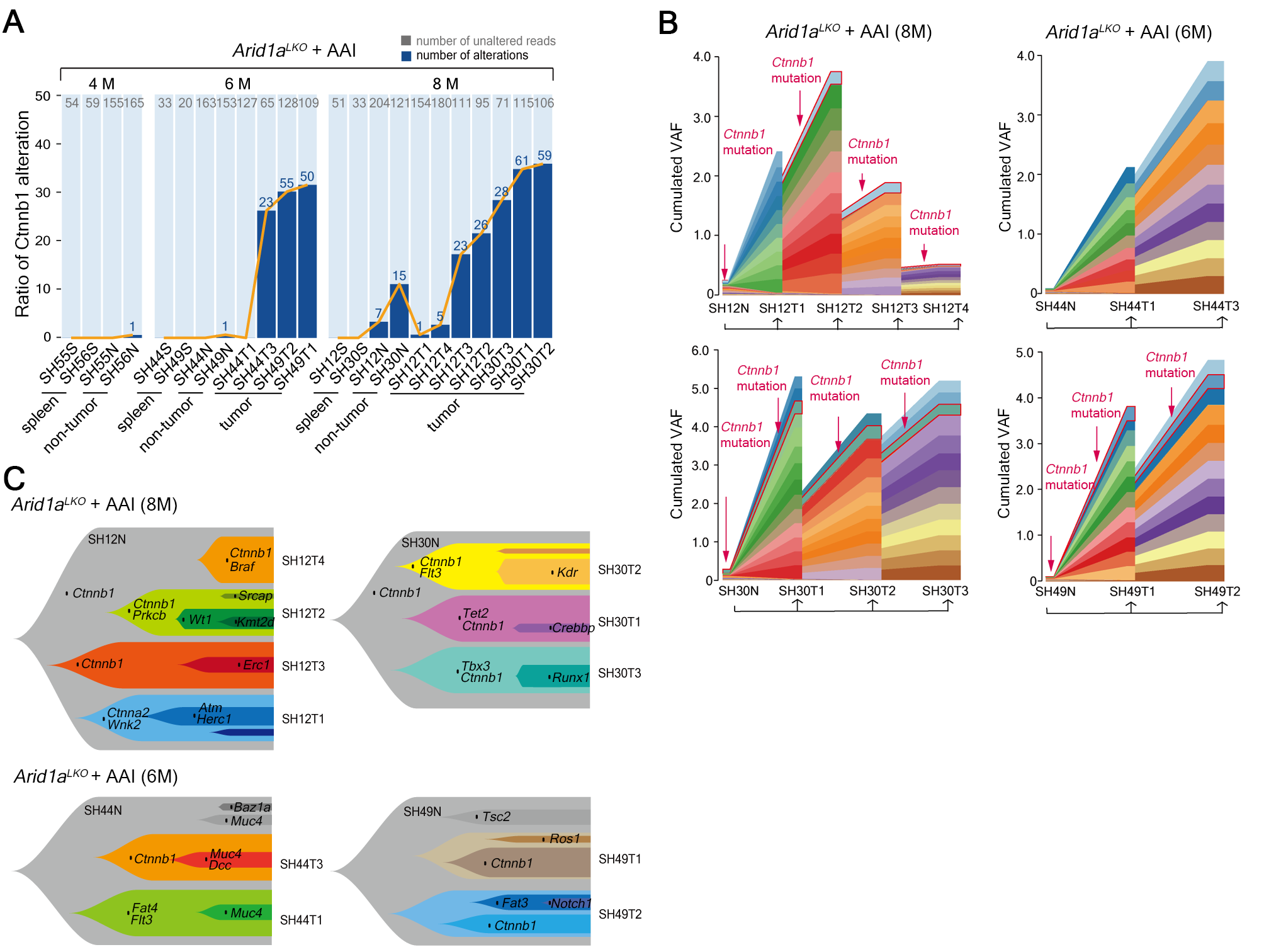
Figure S10, Supporting Information**

**Figure S10.** Clone evolution of *Ctnnb1* mutation during liver tumorigenesis in AAI-treated *Arid1a^LKO^* mice. A) Variant allele frequencies (VAFs) of *Ctnnb1* splicing site mutation (Chr9:120950520 A>T) in spleens, adjacent non-tumorous liver tissues, and tumors from AAI-treated *Arid1a^LKO^* mice. The number of original reads of *Ctnnb1* covering the specific site (Chr9: 120950520) was recalculated from raw WES data. Grey numbers: reference allele counts; dark blue numbers: variant allele counts. B) Rainbow plots showing VAFs of mutations in adjacent non-tumorous tissues that increased in tumor nodules from four 6- or 8-month-old AAI-treated *Arid1a^LKO^* mice. Each color represents a nonsynonymous mutation detected in adjacent non-tumorous tissues. Red outline: *Ctnnb1* mutation (Chr9:120950520 A>T). C) Abstract visualization of malignant clonal evolution and subclonal structures based on the somatic mutations in liver tissues from AAI-treated *Arid1a^LKO^* mice.

**Figure S11, Supporting Information**

**
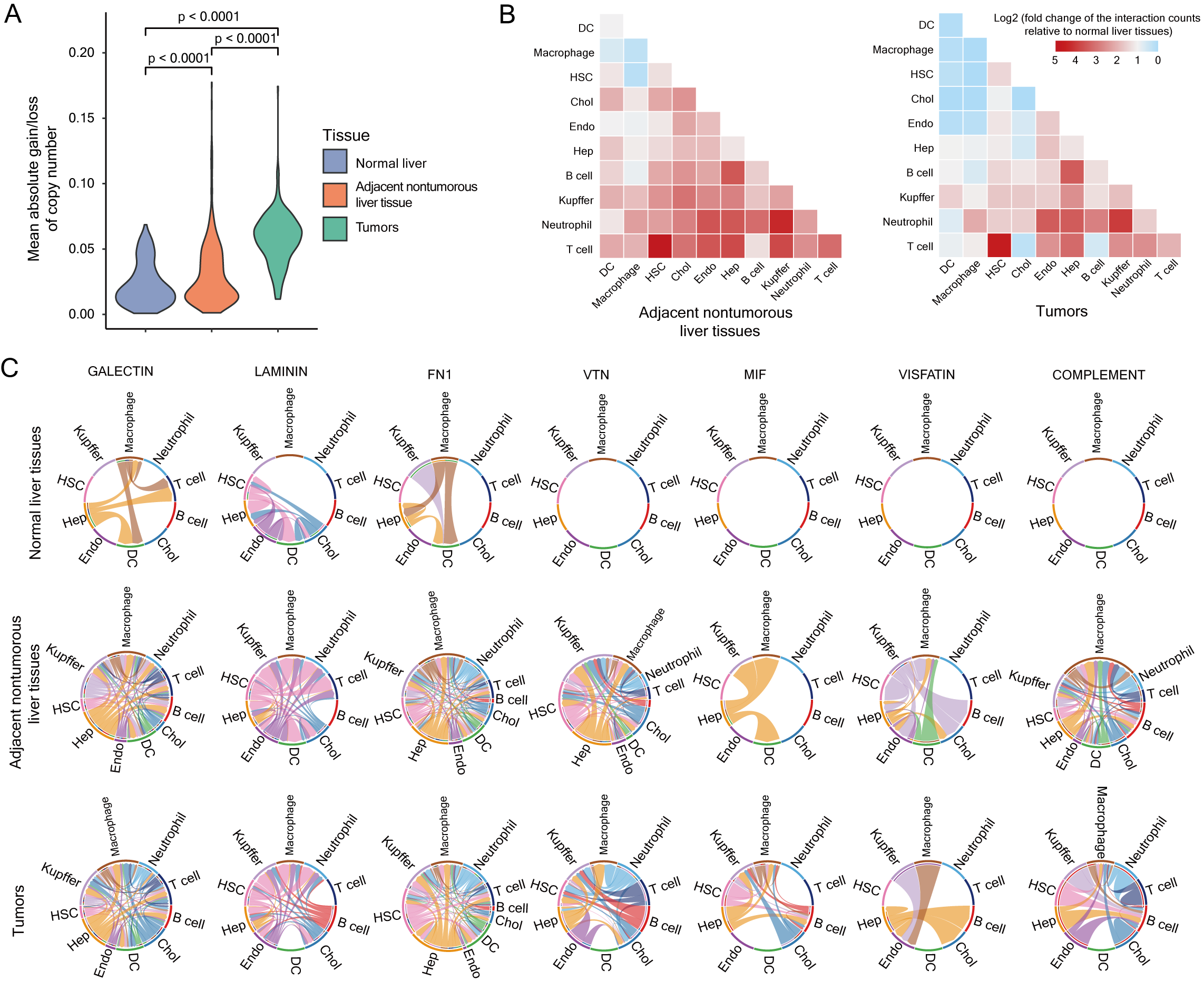
**

**Figure S11.** Cell-cell interaction analysis based on snRNA-seq data from mouse livers. A) CNV analysis using snRNA-seq data calculated by InferCNV in normal livers (WT mice), adjacent non-tumorous liver tissues (AAI-treated *Arid1a^LKO^* mice) and liver tumors (AAI-treated *Arid1a^LKO^* mice). *P* values: two-tailed Student’s *t*-test. B) Cell interaction activities in adjacent non-tumorous liver tissues (left) and tumors (right) of AAI-treated *Arid1a^LKO^* mice comparing to normal liver tissues of wild-type mice, analyzed using CellphoneDB software. C) Visualization of cell interaction activities of selected signaling pathways in normal liver tissues of wild-type mice (top), adjacent non-tumorous liver tissues (middle), and tumors (bottom) of AAI-treated *Arid1a^LKO^* mice, analyzed using Cellchat software.

**
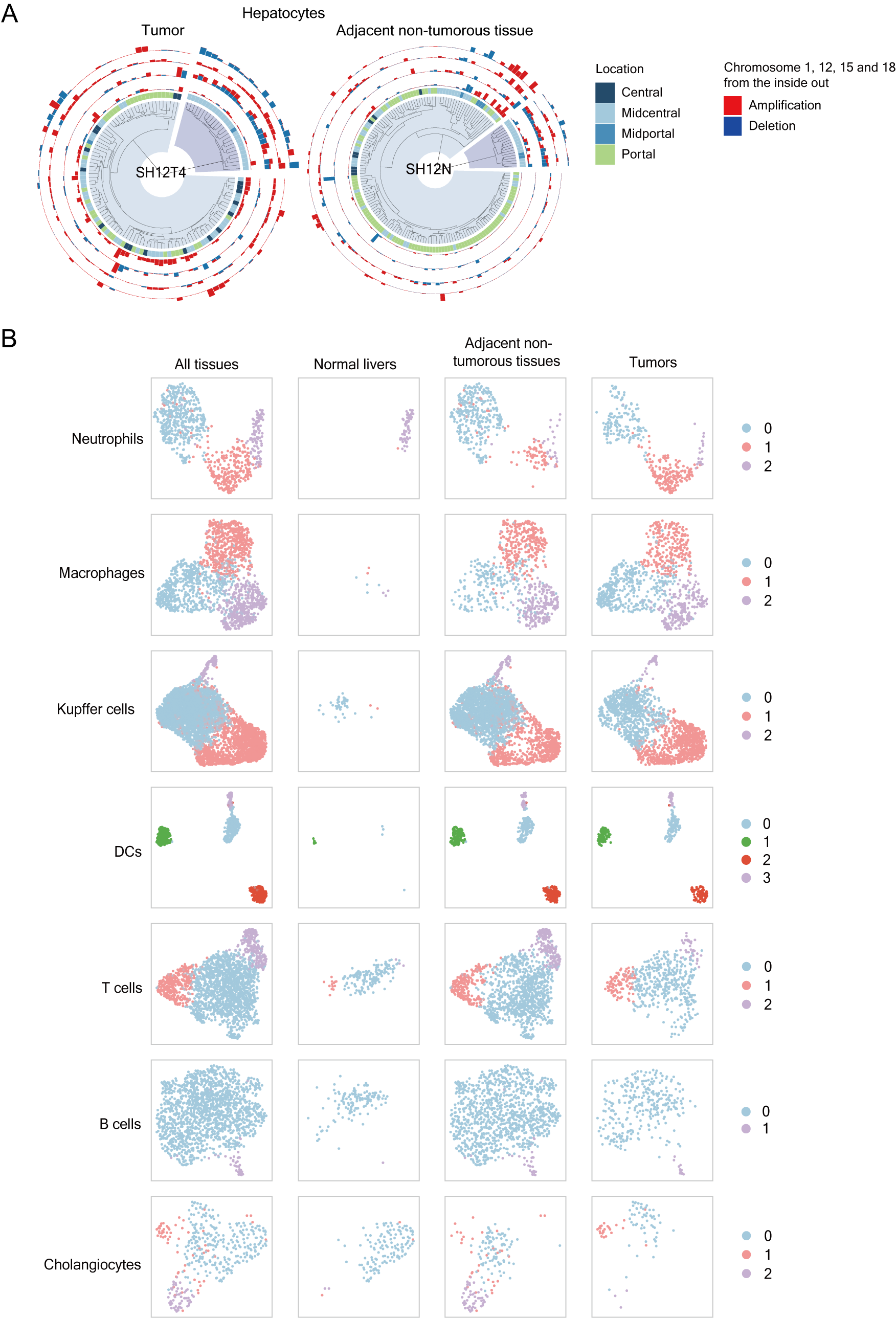
Figure S12, Supporting Information**

**Figure S12.** Integrated clonal and subcluster analysis. A) Genomic CNVs and clonal structure in hepatocytes from tumor (SH12T4, left) and adjacent non-tumorous tissue (SH12N, right) of an AAI-treated *Arid1a^LKO^* mouse. The circle diagrams, from inside to outside, represent clone clusters, cell location, and CNVs at the chromosomal level, respectively. B) UMAP plots of immune cell and cholangiocyte subclusters.

**
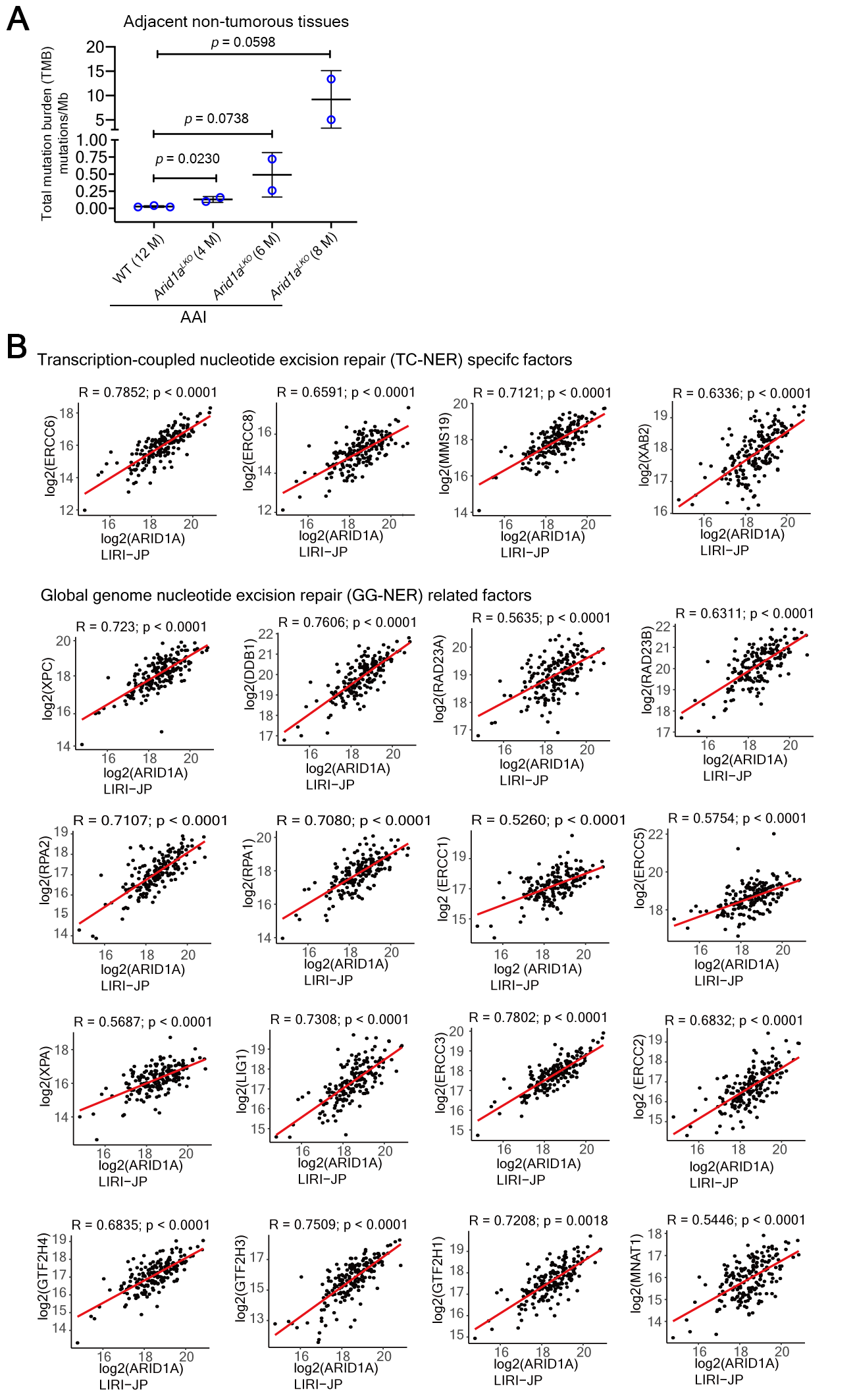
Figure S13, Supporting Information**

**Figure S13.** TMB analysis in mouse tissues and *ARID1A* expression correlation with critical NER genes in HCCs. A) TMB in adjacent non-tumorous livers from AAI-treated wild-type (n = 3) and *Arid1a^LKO^* (4 M, n = 2; 6 M, n = 2; 8 M, n = 2) mice. Data are represented as means ± s.d.. *P* values: two-tailed Student’s *t*-test. B) *ARID1A*-NER-related gene expression correlations in HCCs from TCGA database. R, Pearson correlation coefficient. *P* values of R: two-tailed Student’s *t*-test.

**Figure S14, Supporting Information**

**
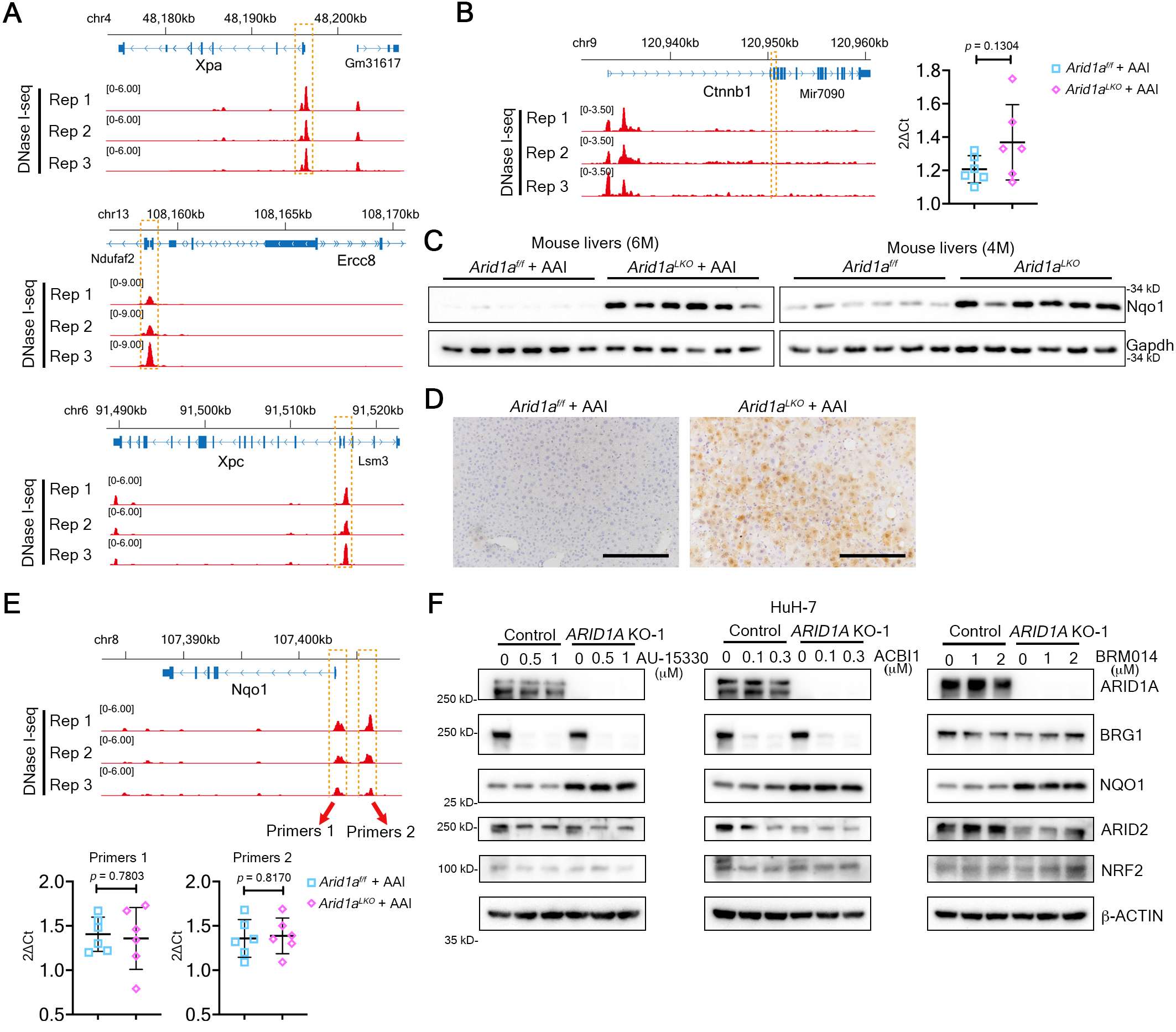
**

**Figure S14.** Analysis of chromatin accessibility of key genes and experimental validation. A) DNase-seq data from 2-month-old wild-type mouse livers were obtained from ENCODE (Rep 1, ENCFF100XUL; Rep 2, ENCFF178LXB; Rep 3, ENCFF445QMM). Orange rectangles highlight DNase I hypersensitive sites (DHSs) around the promoter regions of *Xpa* (top), *Ercc8* (middle) and *Xpc* (bottom). B) DNase-seq data of 2-month-old wild-type mice (as in A). The orange rectangle marks the *Ctnnb1* mutation site induced by AAI (left). DNase I sensitivity was evaluated using real-time PCR by 2^∆Ct^ (right) (n = 6 for each group). Data are represented as means ± s.d.. *P* values, two-tailed Student’s *t*-test. C) Western blotting of non-tumorous liver tissues from 6-month-old AAI-treated *Arid1a^f/f^* and *Arid1a^LKO^* mice (top) and 4-month-old *Arid1a^f/f^* and *Arid1a^LKO^* mice (bottom) using indicated antibodies. Gapdh served as a loading control. D) Representative immunohistochemistry images of liver tissue sections from 6-month-old AAI-treated *Arid1a^f/f^* and *Arid1a^LKO^* mice, stained with indicated antibodies (n ≥ 5). Scale bar, 200 μm. E) DNase-seq data from livers of 2-month-old wild-type mice (as in A). Orange rectangles indicate DHSs around *Nqo1* promoter (top). DNase I sensitivity was evaluated using real-time PCR by 2^∆Ct^ (bottom) (n = 6 for each group). Data are represented as means ± s.d.. *P* values, two-tailed Student’s *t*-test. F) Western blotting of HuH-7 cells treated for 20 hours with Brg1/Brm inhibitor AU-15330 (left), ACBI1 (middle), and BRM014 (right), using indicated antibodies. β-ACTIN served as loading control.

**Figure S15, Supporting Information**

**
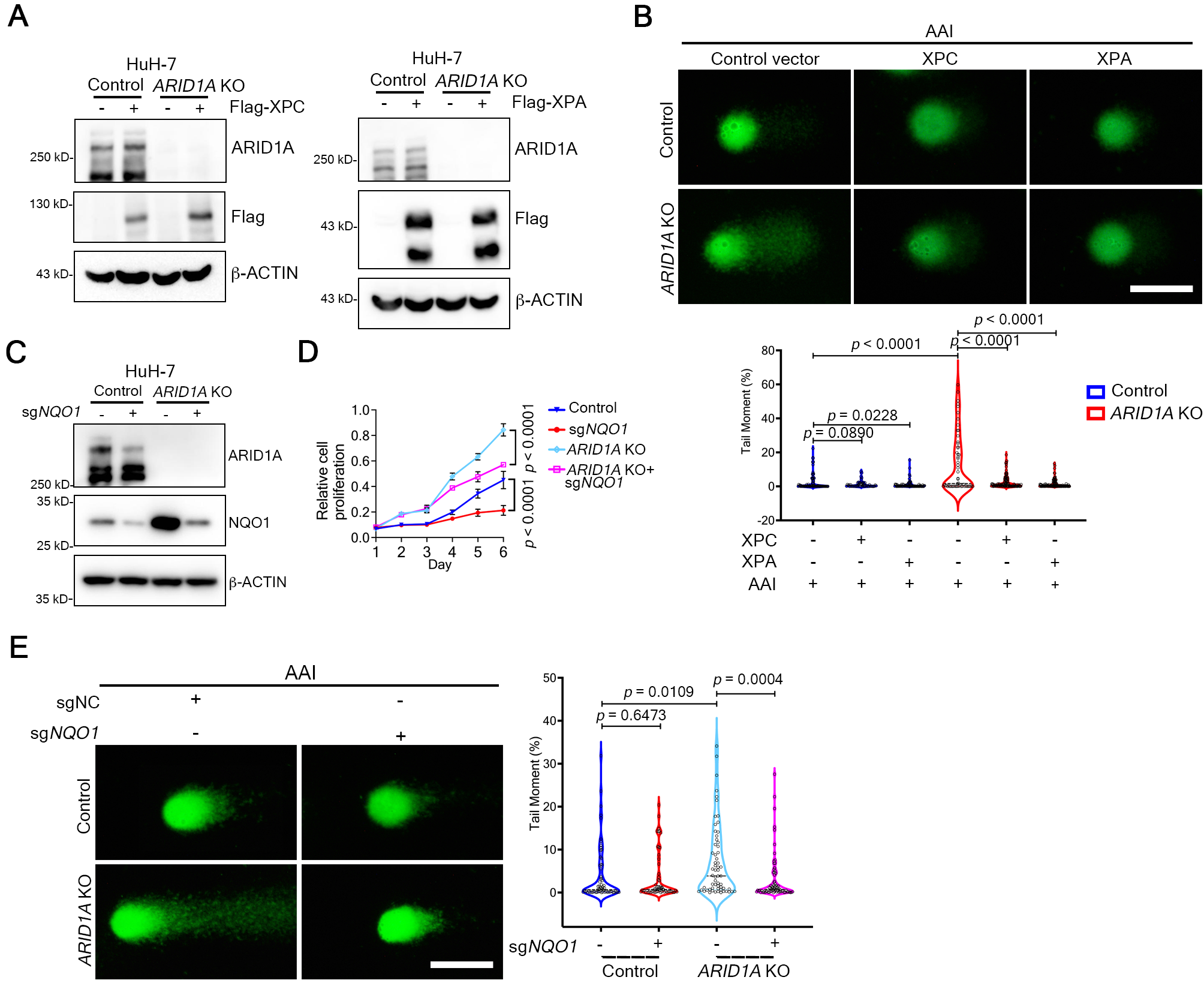
**

**Figure S15.** *XPC* and *XPA* overexpression or *NQO1* inhibition mitigate AAI-induced DNA damage in *ARID1A*-knockout HuH-7 cells. A) Western blotting of the expression of transfected Flag-tagged XPC (left) and XPA (right) in control and *ARID1A*-knockout HuH-7 cells. B) Representative comet assays of HuH-7 cells (top). Original magnification, ×20. Scale bar, 50 μm. Violin plot of the quantification of the tail moment using OpenComet plugin of ImageJ (bottom). At least 50 cells was calculated for each group. *P* values: two-tailed Student’s *t*-test. C) Western blotting of control and *ARID1A*-knockout HuH-7 cells using indicated antibodies. D) Cell proliferation detected using CCK8. Data were represented as means ± SEM. Replicates, n = 3. *P* values: two-way ANOVA. E) Cells in (C) were treated with AAI (12.5 μM) for 48 h and subjected to comet assays. Representative comet assays of HuH-7 cells (left). Original magnification, ×20. Scale bar, 50 μm. Violin plot of the quantification of the tail moment using OpenComet plugin of ImageJ (right). At least 50 cells was calculated for each group. *P* values: two-tailed Student’s *t*-test.

**Figure S16, Supporting Information**

**
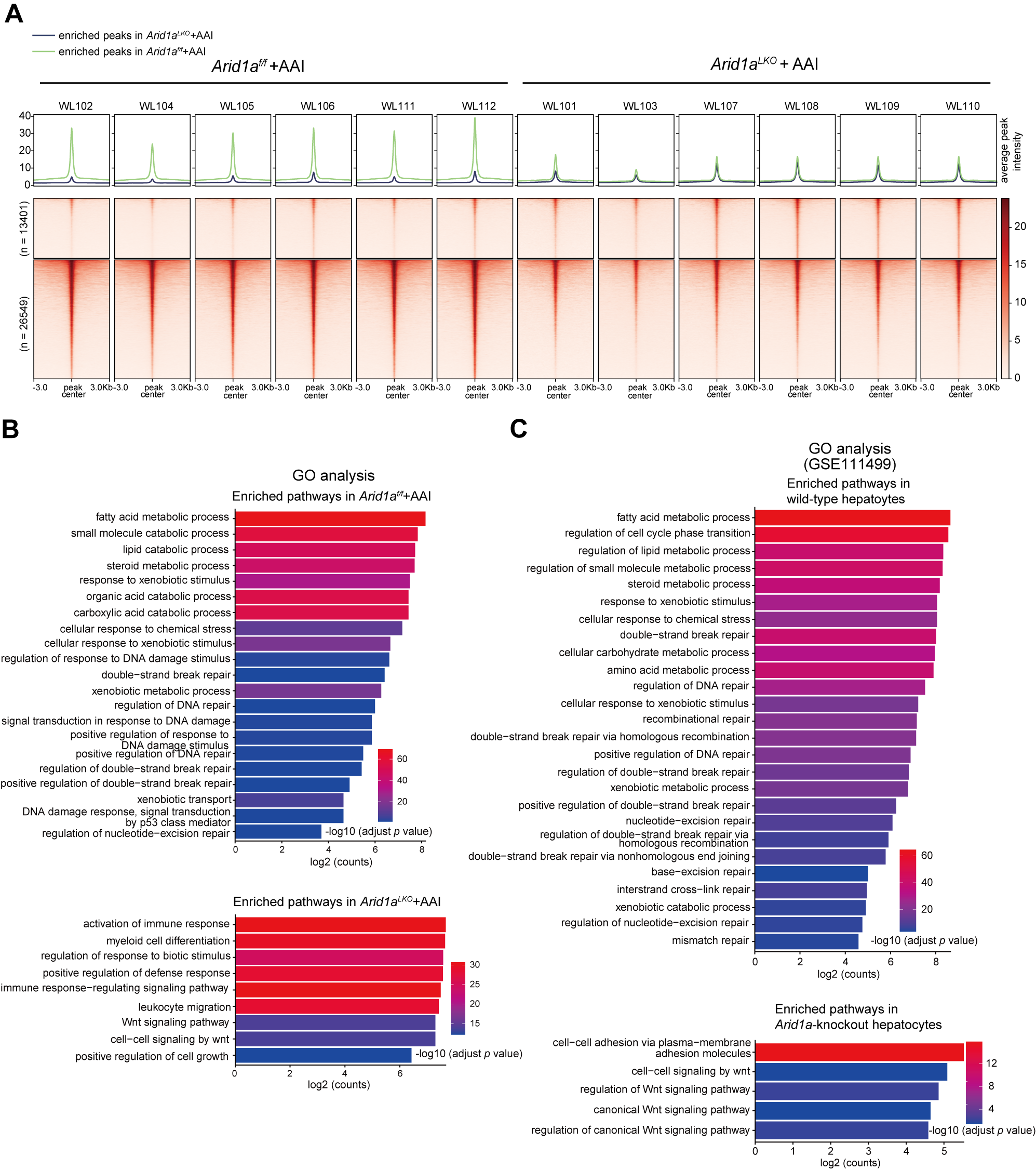
**

**Figure S16.** *Arid1a* deficiency alters chromatin accessibility. A) Differential ATAC-seq peaks in liver tissues from 8-month-old AAI-treated *Arid1a^f/f^* mice (n = 6) compared to *Arid1a^LKO^* (n = 6) (*p* < 0.05). B) GO analysis of ATAC-seq peaks enriched in livers form *Arid1a^f/f^* mice (top) and *Arid1a^LKO^* (bottom). C) ATAC-seq data of hepatocytes isolated from wild-type and *Arid1a*-knockout mice was downloaded from GEO database (GSE111499). GO analysis of ATAC-seq peaks enriched in hepatocytes form wild-type mice (top) and *Arid1a*-knockout mice (bottom).

**Figure S17, Supporting Information**

**
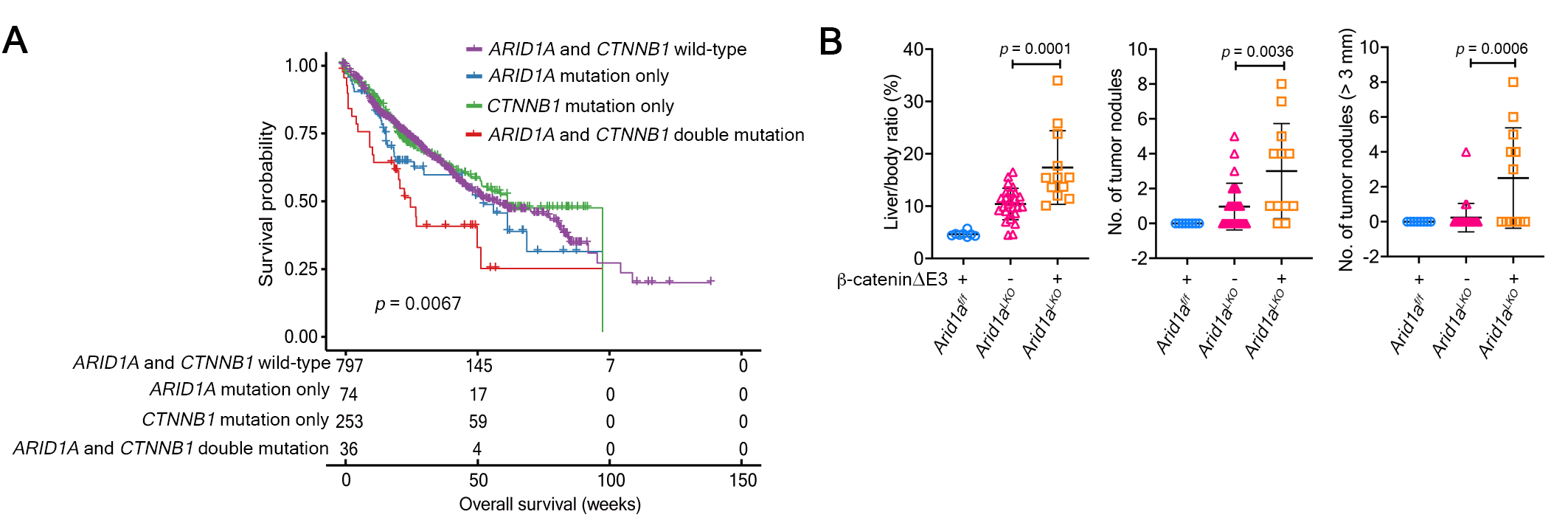
**

**Figure S17.** *Ctnnb1* mutation cooperates with *Arid1a* deficiency to promote tumor formation. A) Survival curves of HCC patients derived from TCGA, ICGC and published datasets, stratified by *ARID1A* and *CTNNB1* mutations. *P* value: Log rank test. B) Statistical analysis of liver/body weight ratio, total tumor nodules, and number of tumor nodules (> 3 mm) in different mouse groups with or without hydrodynamic injection of β-catenin-ΔE3 (*Arid1a^f/f^ +* β-catenin-ΔE3, n = 8; *Arid1a^LKO^* in Figure 1C, n = 26; *Arid1a^LKO^* + β-catenin-ΔE3, n = 12). Data are represented as means ± s.d.. *P* values: two-tailed Student’s *t*-test.

**Figure S18, Supporting Information**

**
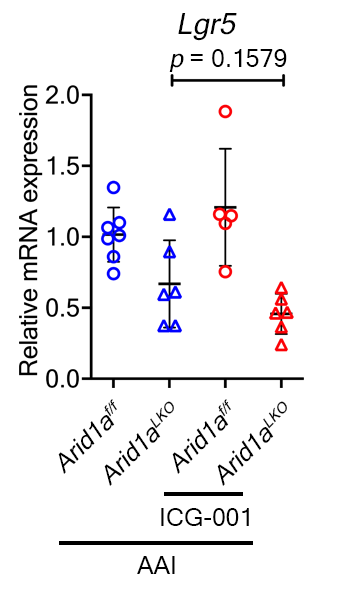
**

**Figure S18.** RT-PCR of *Lgr5* in the non-tumorous liver tissues from AAI-treated *Arid1a^f/f^* (n = 7), AAI-treated *Arid1a^LKO^* (n = 6), AAI and ICG-001-treated *Arid1a^f/f^* (n = 5), and AAI and ICG-001-treated *Arid1a^LKO^* (n = 6) mice. Data were represented as means ± s.d.. *P* values: two-tailed Student’s *t*-test.

**Figure S19, Supporting Information**

**
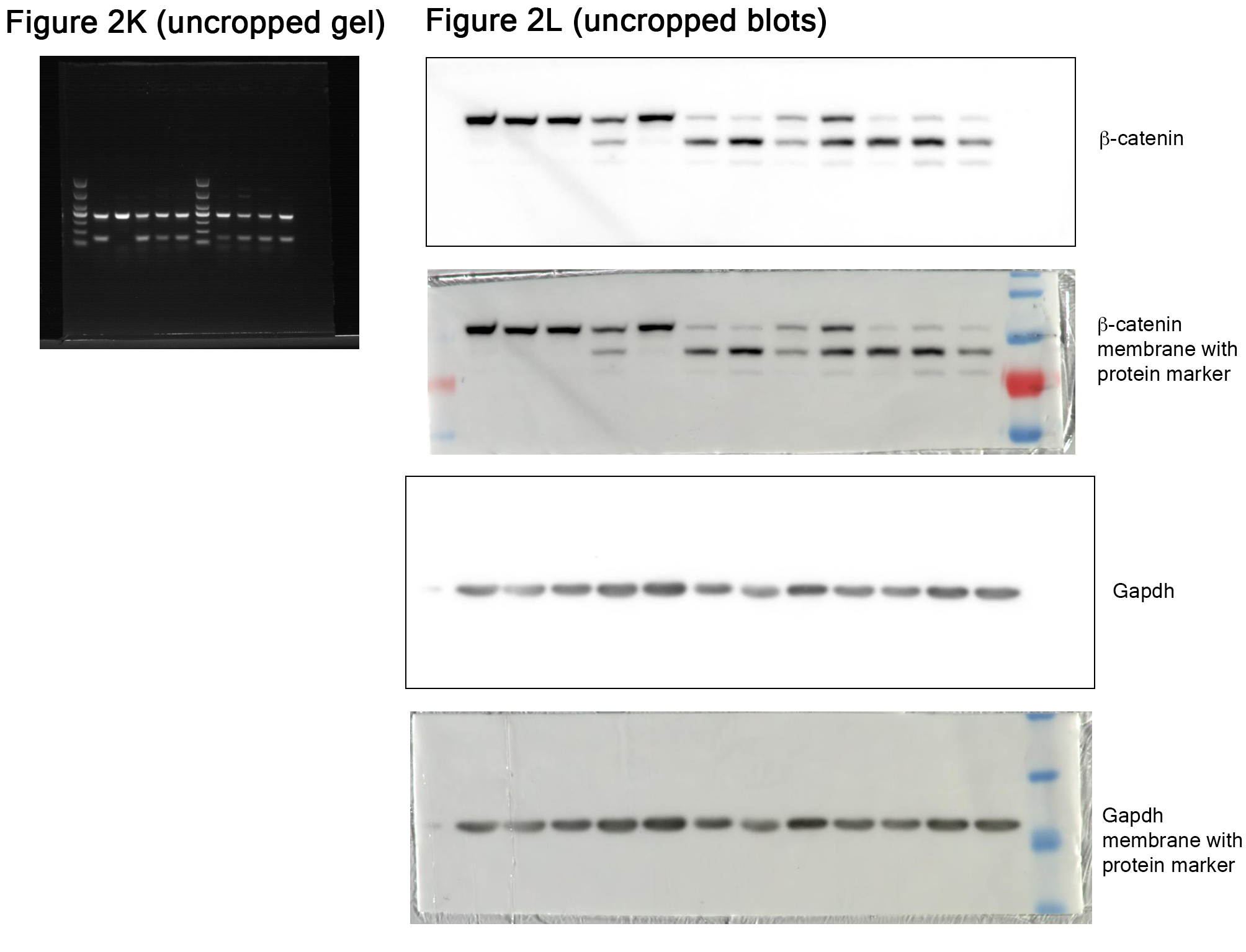
**

**Figure S19.** Uncropped gel for Figure 2K and uncropped western blot membranes for Figure 2L.

**
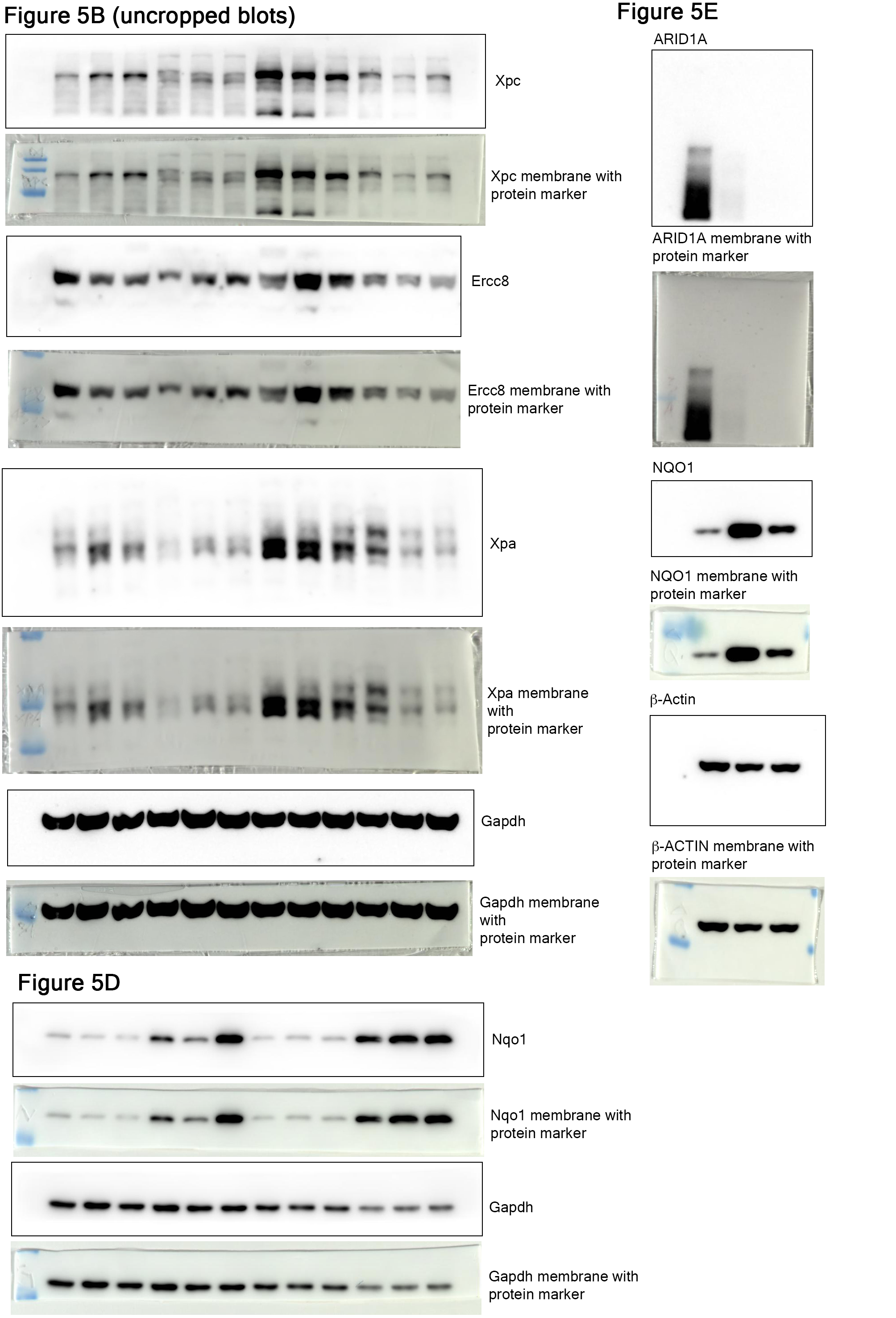
Figure S20, Supporting Information**

**Figure S20.** Uncropped western blot membranes for Figure 5B, 5D and 5E.

**
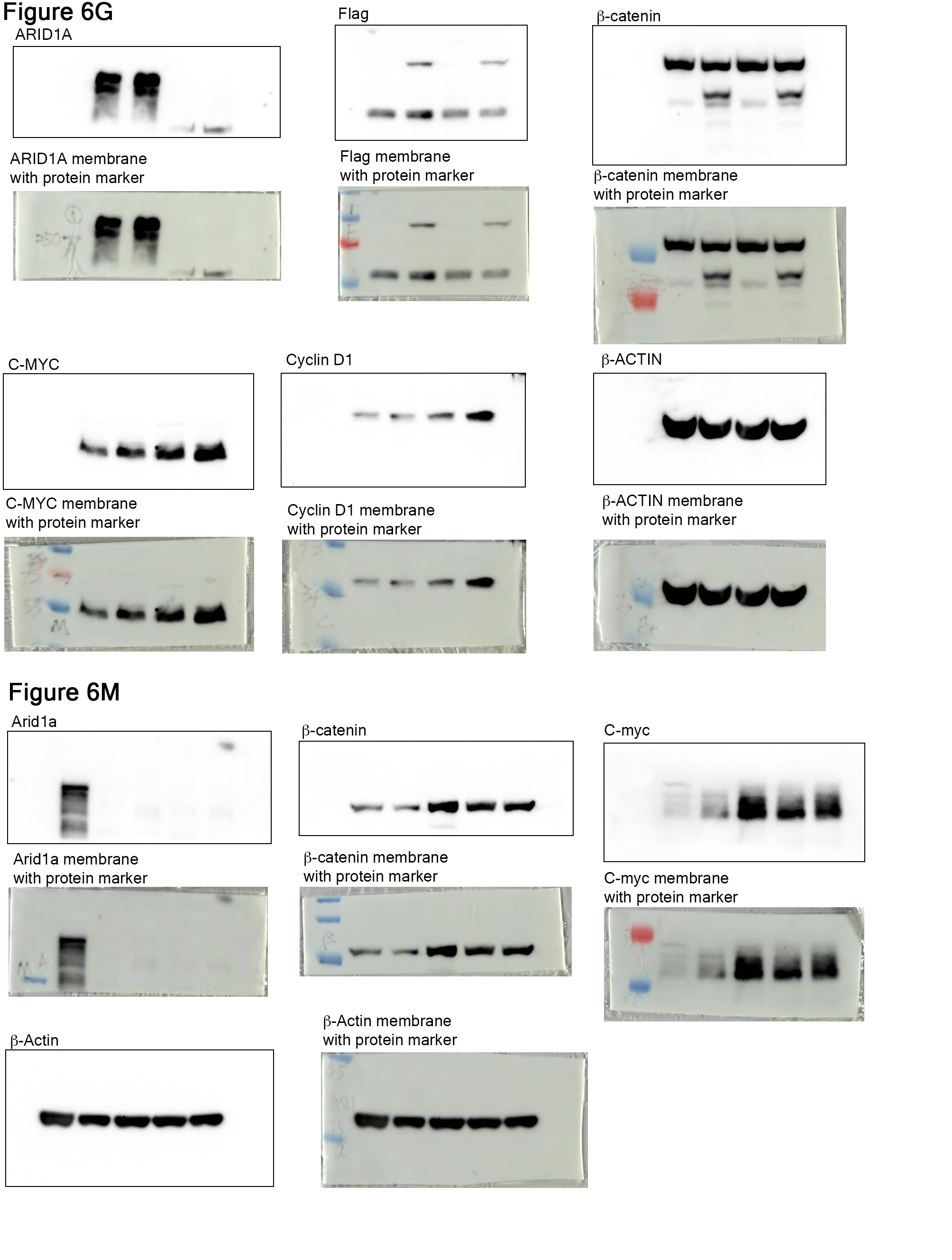
Figure S21, Supporting Information**

**Figure S21.** Uncropped western blot membranes for Figure 6G and 6M.

**
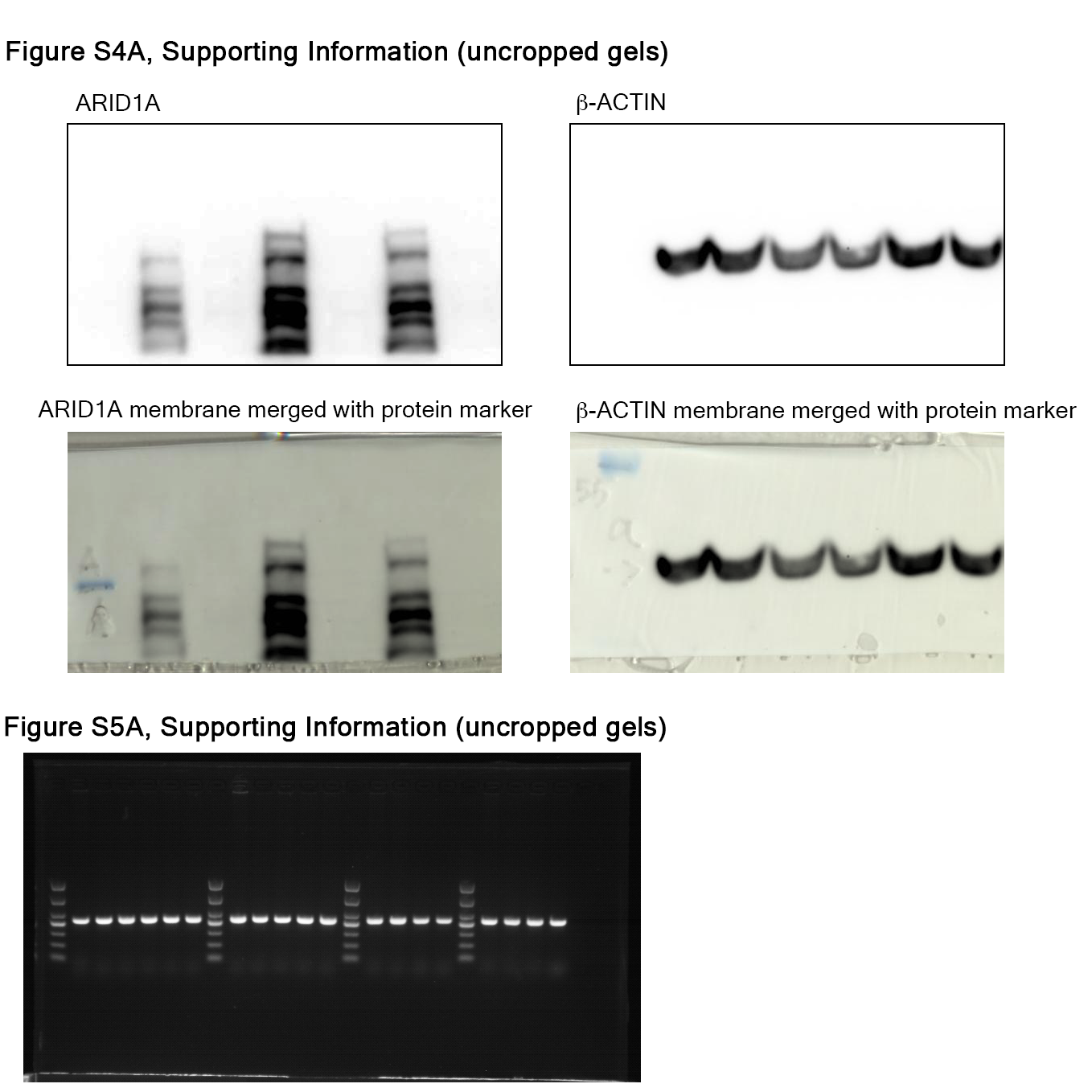
Figure S22, Supporting Information**

**Figure S22.** Uncropped gel for Figure S4A and S5A, Supporting Information.

**
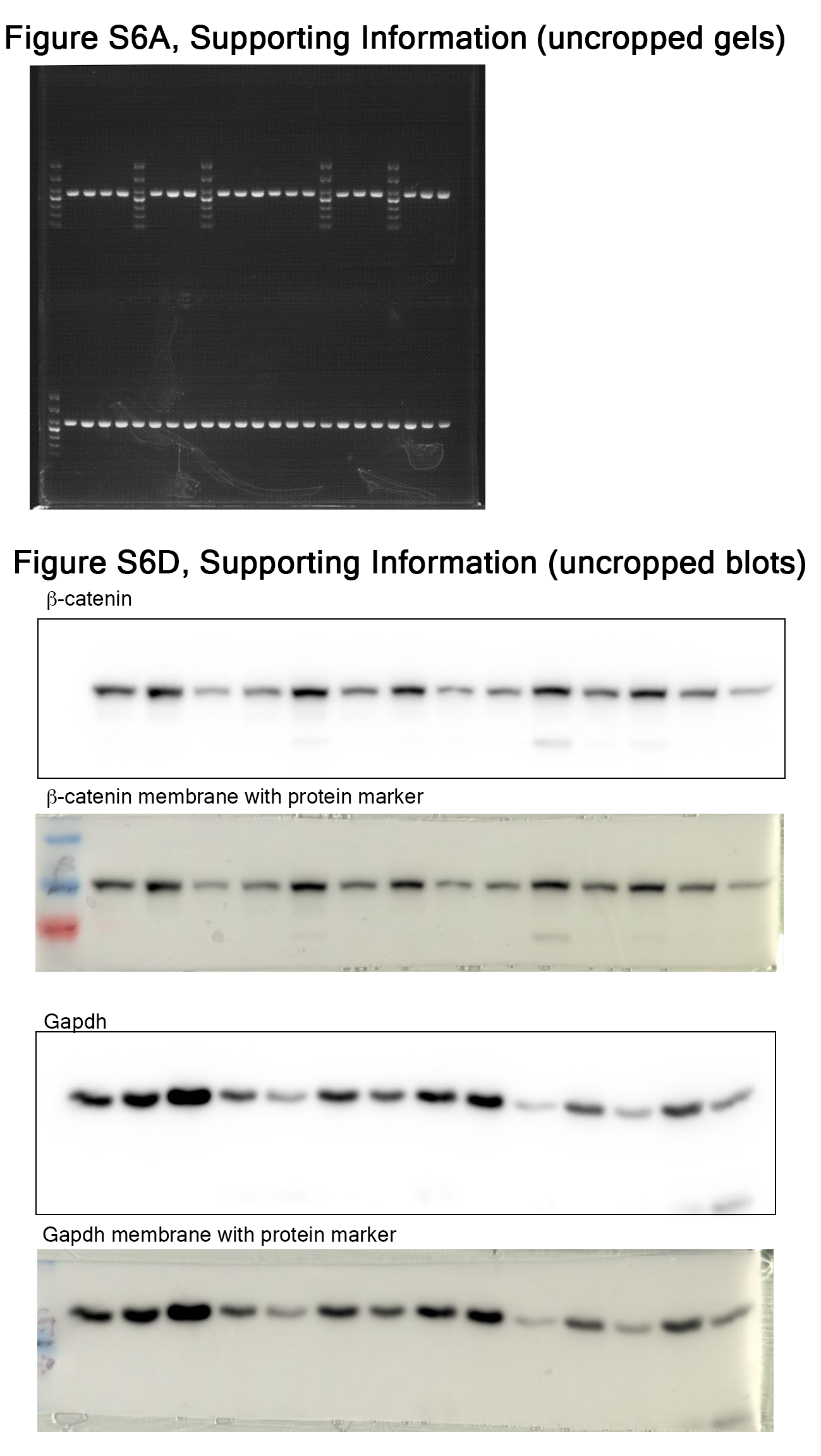
Figure S23, Supporting Information**

**Figure S23.** Uncropped gel for Figure S6A, Supporting Information and uncropped western blot membranes for Figure S6D, Supporting Information.

**
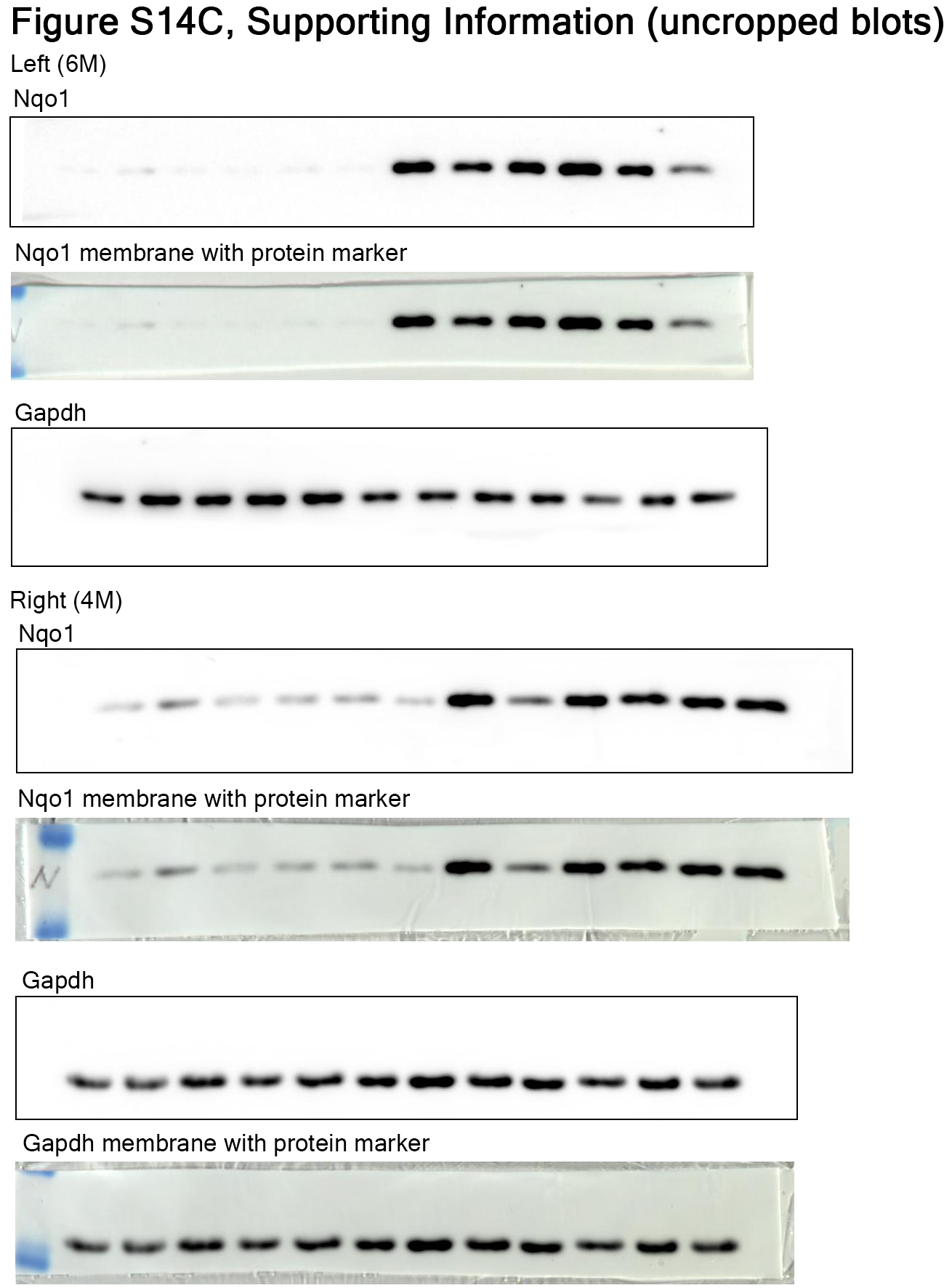
Figure S24, Supporting Information**

**Figure S24.** Uncropped western blot membranes for Figure S14C, Supporting Information.

**Figure S25, Supporting Information**

**
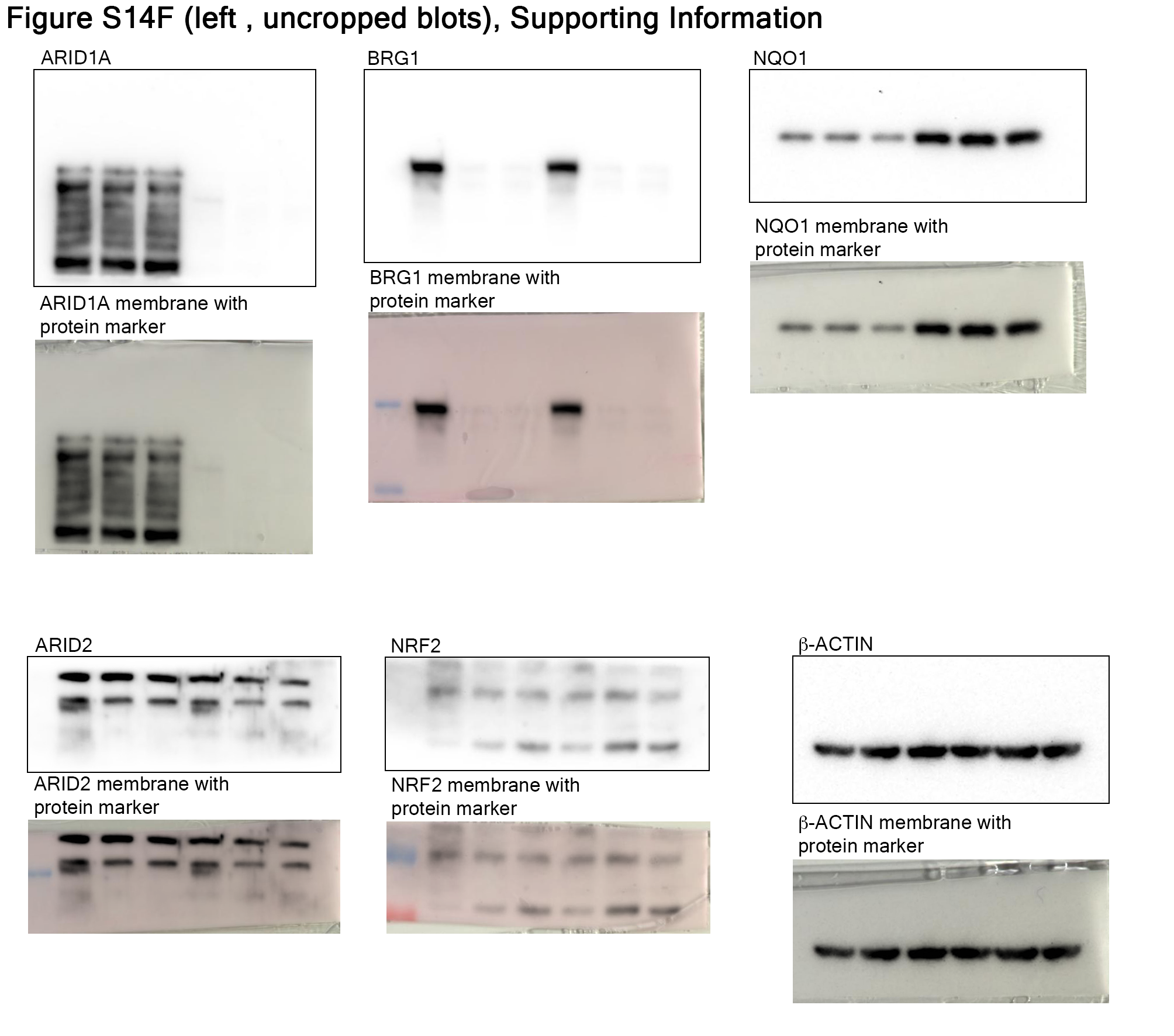
**

**Figure S25.** Uncropped western blot membranes for Figure S14F (left), Supporting Information.

**Figure S26, Supporting Information**

**Figure S26.** Uncropped western blot membranes for Figure S14F (middle), Supporting Information.**
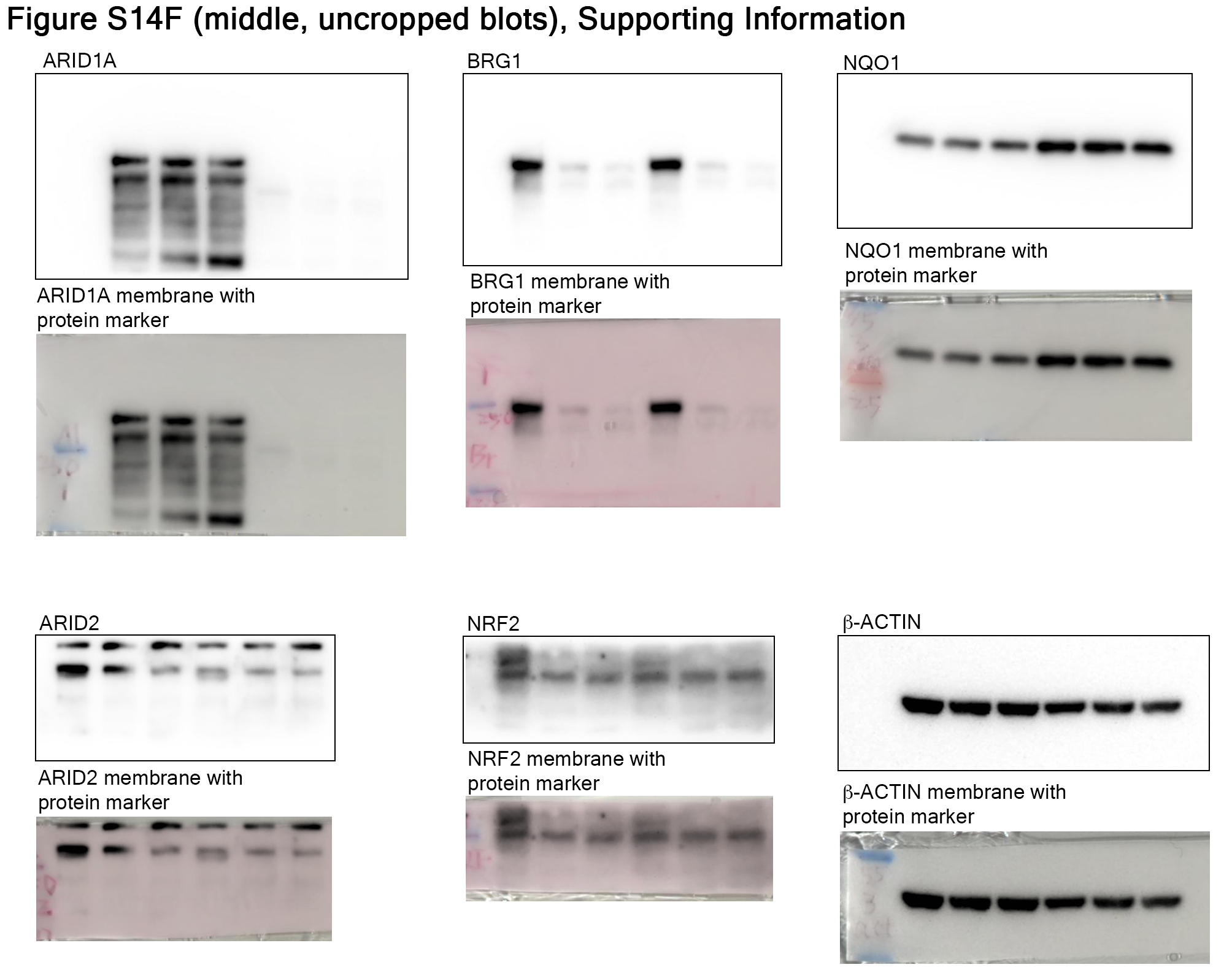
**

**Figure S27, Supporting Information**

**
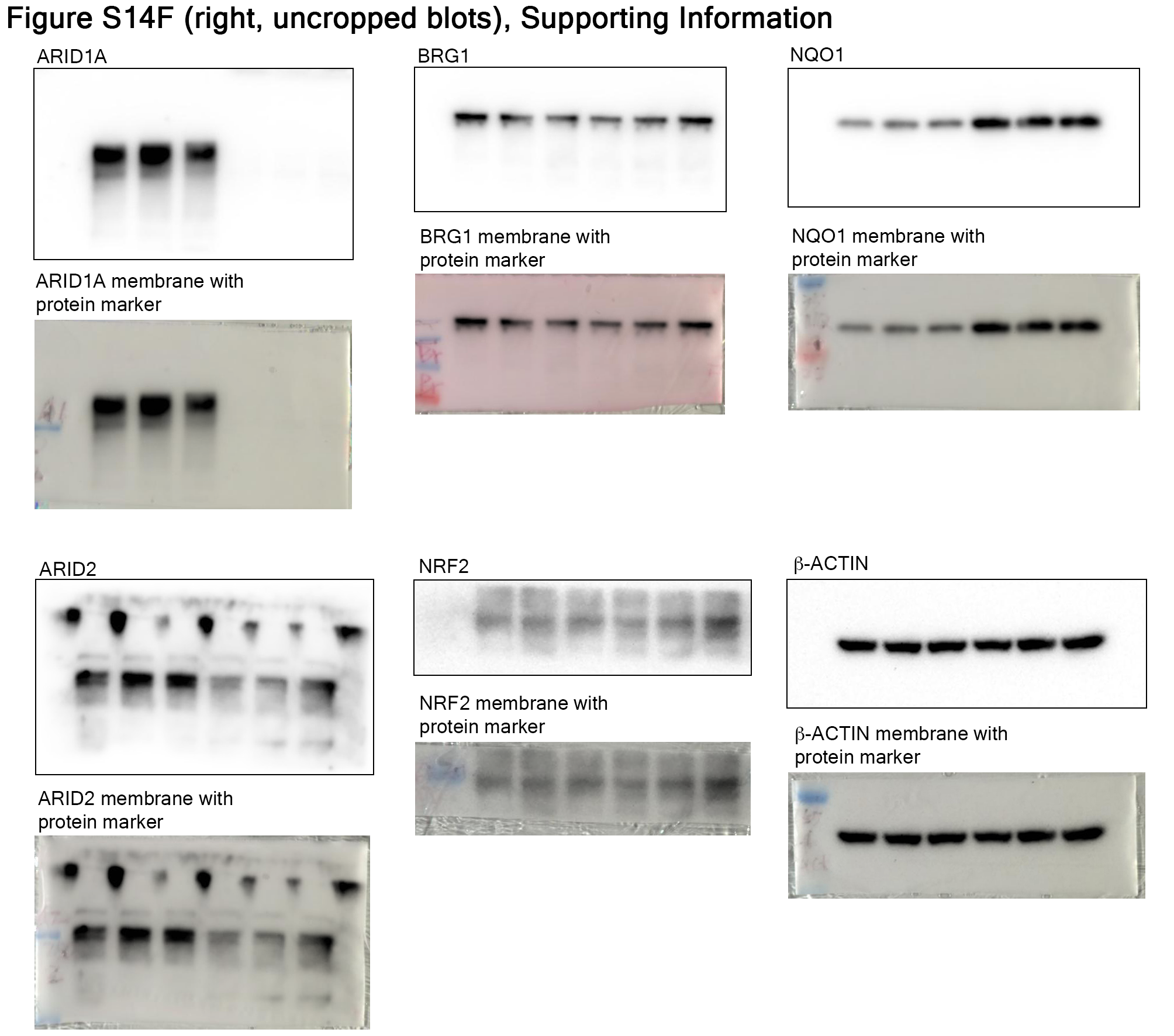
**

**Figure S27.** Uncropped western blot membranes for Figure S14F (right), Supporting Information.

**
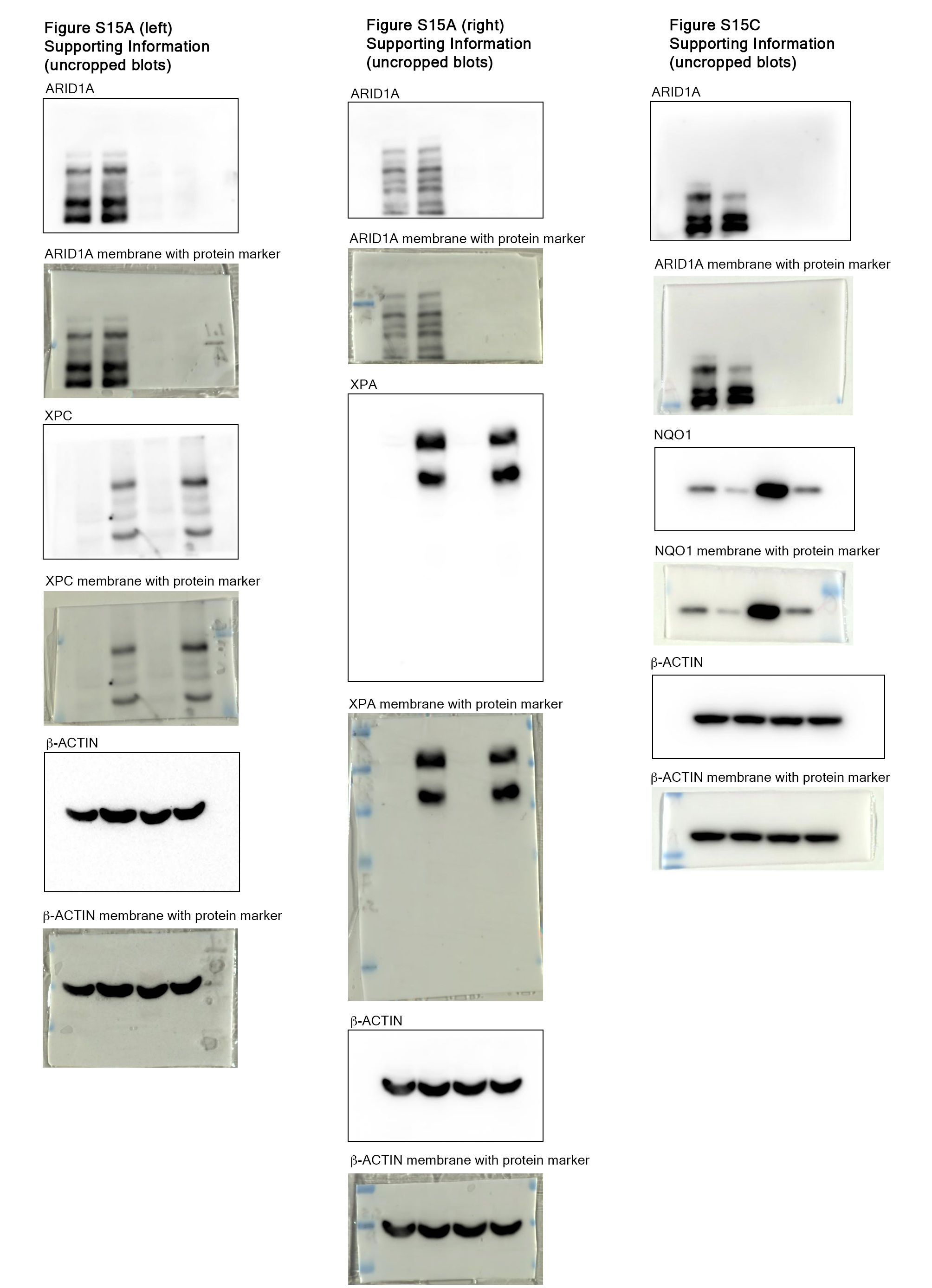
Figure S28, Supporting Information**

**Figure S28.** Uncropped western blot membranes for Figure S15A and S15C, Supporting Information.

**3. Supplementary references**

[1] H. Li, R. Durbin, “Fast and accurate short read alignment with Burrows-Wheeler transform,” *Bioinformatics.* **2009**, 25, 1754-1760.

[2] H. Li, B. Handsaker, A. Wysoker, et al., “The Sequence Alignment/Map format and SAMtools,” *Bioinformatics.* **2009**, 25, 2078-2079.

[3] A. McKenna, M. Hanna, E. Banks, et al., “The Genome Analysis Toolkit: a MapReduce framework for analyzing next-generation DNA sequencing data,” *Genome Res.* **2010**, 20, 1297-1303.

[4] K. Cibulskis, M. S. Lawrence, S. L. Carter, et al., “Sensitive detection of somatic point mutations in impure and heterogeneous cancer samples,” *Nat Biotechnol.* **2013**, 31, 213-219.

[5] S. T. Sherry, M. H. Ward, M. Kholodov, et al., “dbSNP: the NCBI database of genetic variation,” *Nucleic Acids Res.* **2001**, 29, 308-311.

[6] Z. N. Lu, Q. Luo, L. N. Zhao, et al., “The Mutational Features of Aristolochic Acid-Induced Mouse and Human Liver Cancers,” *Hepatology.* **2020**, 71, 929-942.

[7] K. Wang, M. Li, H. Hakonarson, “ANNOVAR: functional annotation of genetic variants from high-throughput sequencing data,” *Nucleic Acids Res.* **2010**, 38, e164.

[8] F. Manders, A. M. Brandsma, J. de Kanter, et al., “MutationalPatterns: the one stop shop for the analysis of mutational processes,” *BMC Genomics.* **2022**, 23, 134.

[9] J. Shinde, Q. Bayard, S. Imbeaud, et al., “Palimpsest: an R package for studying mutational and structural variant signatures along clonal evolution in cancer,” *Bioinformatics.* **2018**, 34, 3380-3381.

[10] V. Boeva, T. Popova, K. Bleakley, et al., “Control-FREEC: a tool for assessing copy number and allelic content using next-generation sequencing data,” *Bioinformatics.* **2012**, 28, 423-425.

[11] E. Talevich, A. H. Shain, T. Botton, B. C. Bastian, “CNVkit: Genome-Wide Copy Number Detection and Visualization from Targeted DNA Sequencing,” *PLoS Comput Biol.* **2016**, 12, e1004873.

[12] C. A. Miller, B. S. White, N. D. Dees, et al., “SciClone: inferring clonal architecture and tracking the spatial and temporal patterns of tumor evolution,” *PLoS Comput Biol.* **2014**, 10, e1003665.

[13] H. X. Dang, B. S. White, S. M. Foltz, et al., “ClonEvol: clonal ordering and visualization in cancer sequencing,” *Ann Oncol.* **2017**, 28, 3076-3082.

[14] B. Langmead, S. L. Salzberg, “Fast gapped-read alignment with Bowtie 2,” *Nat Methods.* **2012**, 9, 357-359.

[15] Y. Liao, G. K. Smyth, W. Shi, “featureCounts: an efficient general purpose program for assigning sequence reads to genomic features,” *Bioinformatics.* **2014**, 30, 923-930.

[16] S. Anders, W. Huber, “Differential expression analysis for sequence count data,” *Genome Biol.* **2010**, 11, R106.

[17] Z. Tian, C. Wang, M. Guo, X. Liu, Z. Teng, “An improved method for functional similarity analysis of genes based on Gene Ontology,” *BMC Syst Biol.* **2016**, 10, 119.

[18] M. Kanehisa, M. Araki, S. Goto, et al., “KEGG for linking genomes to life and the environment,” *Nucleic Acids Res.* **2008**, 36, D480-484.

[19] G. Yu, L. G. Wang, Y. Han, Q. Y. He, “clusterProfiler: an R package for comparing biological themes among gene clusters,” *OMICS.* **2012**, 16, 284-287.

[20] A. Subramanian, H. Kuehn, J. Gould, P. Tamayo, J. P. Mesirov, “GSEA-P: a desktop application for Gene Set Enrichment Analysis,” *Bioinformatics.* **2007**, 23, 3251-3253.

[21] S. Hanzelmann, R. Castelo, J. Guinney, “GSVA: gene set variation analysis for microarray and RNA-seq data,” *BMC Bioinformatics.* **2013**, 14, 7.

[22] A. Butler, P. Hoffman, P. Smibert, E. Papalexi, R. Satija, “Integrating single-cell transcriptomic data across different conditions, technologies, and species,” *Nat Biotechnol.* **2018**, 36, 411-420.

[23] C. S. McGinnis, L. M. Murrow, Z. J. Gartner, “DoubletFinder: Doublet Detection in Single-Cell RNA Sequencing Data Using Artificial Nearest Neighbors,” *Cell Syst.* **2019**, 8, 329-337 e324.

[24] D. Aran, A. P. Looney, L. Liu, et al., “Reference-based analysis of lung single-cell sequencing reveals a transitional profibrotic macrophage,” *Nat Immunol.* **2019**, 20, 163-172.

[25] X. Shao, J. Liao, X. Lu, et al., “scCATCH: Automatic Annotation on Cell Types of Clusters from Single-Cell RNA Sequencing Data,” *iScience.* **2020**, 23, 100882.

[26] R. Nault, K. A. Fader, S. Bhattacharya, T. R. Zacharewski, “Single-Nuclei RNA Sequencing Assessment of the Hepatic Effects of 2,3,7,8-Tetrachlorodibenzo-p-dioxin,” *Cell Mol Gastroenterol Hepatol.* **2021**, 11, 147-159.

[27] M. Efremova, M. Vento-Tormo, S. A. Teichmann, R. Vento-Tormo, “CellPhoneDB: inferring cell-cell communication from combined expression of multi-subunit ligand-receptor complexes,” *Nat Protoc.* **2020**, 15, 1484-1506.

[28] S. Jin, C. F. Guerrero-Juarez, L. Zhang, et al., “Inference and analysis of cell-cell communication using CellChat,” *Nat Commun.* **2021**, 12, 1088.

[29] I. Tirosh, B. Izar, S. M. Prakadan, et al., “Dissecting the multicellular ecosystem of metastatic melanoma by single-cell RNA-seq,” *Science.* **2016**, 352, 189-196.

[30] C. Trapnell, D. Cacchiarelli, J. Grimsby, et al., “The dynamics and regulators of cell fate decisions are revealed by pseudotemporal ordering of single cells,” *Nat Biotechnol.* **2014**, 32, 381-386.

[31] J. D. Buenrostro, P. G. Giresi, L. C. Zaba, H. Y. Chang, W. J. Greenleaf, “Transposition of native chromatin for fast and sensitive epigenomic profiling of open chromatin, DNA-binding proteins and nucleosome position,” *Nat Methods.* **2013**, 10, 1213-1218.

[32] L. Q. Dong, L. H. Peng, L. J. Ma, et al., “Heterogeneous immunogenomic features and distinct escape mechanisms in multifocal hepatocellular carcinoma,” *J Hepatol.* **2020**, 72, 896-908.

[33] Q. Gao, H. Zhu, L. Dong, et al., “Integrated Proteogenomic Characterization of HBV-Related Hepatocellular Carcinoma,” *Cell.* **2019**, 179, 561-577 e522.

[34] A. Fujimoto, Y. Totoki, T. Abe, et al., “Whole-genome sequencing of liver cancers identifies etiological influences on mutation patterns and recurrent mutations in chromatin regulators,” *Nat Genet.* **2012**, 44, 760-764.

[35] S. M. Ahn, S. J. Jang, J. H. Shim, et al., “Genomic portrait of resectable hepatocellular carcinomas: implications of RB1 and FGF19 aberrations for patient stratification,” *Hepatology.* **2014**, 60, 1972-1982.

[36] R. Xue, L. Chen, C. Zhang, et al., “Genomic and Transcriptomic Profiling of Combined Hepatocellular and Intrahepatic Cholangiocarcinoma Reveals Distinct Molecular Subtypes,” *Cancer Cell.* **2019**, 35, 932-947 e938.

[37] J. J. Harding, S. Nandakumar, J. Armenia, et al., “Prospective Genotyping of Hepatocellular Carcinoma: Clinical Implications of Next-Generation Sequencing for Matching Patients to Targeted and Immune Therapies,” *Clin Cancer Res.* **2019**, 25, 2116-2126.

[38] J. Zheng, E. Sadot, J. A. Vigidal, et al., “Characterization of hepatocellular adenoma and carcinoma using microRNA profiling and targeted gene sequencing,” *PLoS One.* **2018**, 13, e0200776.

[39] K. Schulze, S. Imbeaud, E. Letouze, et al., “Exome sequencing of hepatocellular carcinomas identifies new mutational signatures and potential therapeutic targets,” *Nat Genet.* **2015**, 47, 505-511.

[40] C. K. Y. Ng, E. Dazert, T. Boldanova, et al., “Integrative proteogenomic characterization of hepatocellular carcinoma across etiologies and stages,” *Nat Commun.* **2022**, 13, 2436.

[41] P. Ramachandran, R. Dobie, J. R. Wilson-Kanamori, et al., “Resolving the fibrotic niche of human liver cirrhosis at single-cell level,” *Nature.* **2019**, 575, 512-518.

[42] W. Li, L. Yang, Q. He, et al., “A Homeostatic Arid1a-Dependent Permissive Chromatin State Licenses Hepatocyte Responsiveness to Liver-Injury-Associated YAP Signaling,” *Cell Stem Cell.* **2019**, 25, 54-68 e55.

[43] A. Mayakonda, D. C. Lin, Y. Assenov, C. Plass, H. P. Koeffler, “Maftools: efficient and comprehensive analysis of somatic variants in cancer,” *Genome Res.* **2018**, 28, 1747-1756.
